# Structure-Based Predictive Models for Allosteric Hot Spots

**DOI:** 10.1371/journal.pcbi.1000531

**Published:** 2009-10-09

**Authors:** Omar N. A. Demerdash, Michael D. Daily, Julie C. Mitchell

**Affiliations:** 1 Biophysics Program, University of Wisconsin-Madison, Madison, Wisconsin, United States of America; 2 Medical Scientist Training Program, University of Wisconsin-Madison, Madison, Wisconsin, United States of America; 3 Department of Chemistry, University of Wisconsin-Madison, Madison, Wisconsin, United States of America; 4 Department of Biochemistry, University of Wisconsin-Madison, Madison, Wisconsin, United States of America; 5 Department of Mathematics, University of Wisconsin-Madison, Madison, Wisconsin, United States of America; National Cancer Institute, United States of America and Tel Aviv University, Israel

## Abstract

In allostery, a binding event at one site in a protein modulates the behavior of a distant site. Identifying residues that relay the signal between sites remains a challenge. We have developed predictive models using support-vector machines, a widely used machine-learning method. The training data set consisted of residues classified as either hotspots or non-hotspots based on experimental characterization of point mutations from a diverse set of allosteric proteins. Each residue had an associated set of calculated features. Two sets of features were used, one consisting of dynamical, structural, network, and informatic measures, and another of structural measures defined by Daily and Gray [1]. The resulting models performed well on an independent data set consisting of hotspots and non-hotspots from five allosteric proteins. For the independent data set, our top 10 models using Feature Set 1 recalled 68–81% of known hotspots, and among total hotspot predictions, 58–67% were actual hotspots. Hence, these models have *precision* P = 58–67% and *recall* R = 68–81%. The corresponding models for Feature Set 2 had P = 55–59% and R = 81–92%. We combined the features from each set that produced models with optimal predictive performance. The top 10 models using this hybrid feature set had R = 73–81% and P = 64–71%, the best overall performance of any of the sets of models. Our methods identified hotspots in structural regions of known allosteric significance. Moreover, our predicted hotspots form a network of contiguous residues in the interior of the structures, in agreement with previous work. In conclusion, we have developed models that discriminate between known allosteric hotspots and non-hotspots with high accuracy and sensitivity. Moreover, the pattern of predicted hotspots corresponds to known functional motifs implicated in allostery, and is consistent with previous work describing sparse networks of allosterically important residues.

## Author Summary

Allostery is the process whereby a molecule binds to one site in a protein and alters the function of a distant site. This phenomenon is ubiquitous, as proteins frequently must adapt their behavior to changes in the cellular milieu. The mechanism(s) underlying allostery remains incompletely understood. In particular, predictive models are needed that distinguish amino-acid residues that are critical to allostery, or “hotspots”, from non-hotspots. Here we have used data-mining approaches to infer rules that distinguish hotspots from non-hotspots. Starting with a data set of known hotspot and non-hotspot residues from a diverse set of allosteric proteins, the training data set, we applied machine learning to this data to “learn” models, or sets of rules, for distinguishing hotspots and non-hotspots by inferring associations between the classification (hotspot or non-hotspot) and an associated set of calculated attributes. Many models that showed the highest predictive power on the training data also exhibited high accuracy and sensitivity when applied to an independent data set. Moreover, the pattern of predicted hotspots in the proteins we studied was consistent with known structure/function relationships and previous work suggesting that a network of essential residues mediates the allosteric transition.

## Introduction

Allostery is the process whereby an effector molecule binds to one site of a protein and concomitantly modulates the function of a distant site. “Allostery” is derived from the Greek *allos*, “other,” and *stereos*, “solid” or “shape,” as the concept was originally applied to proteins that changed their shape, or conformation, upon binding the effector. Although the impact of effector molecules on steady-state catalysis had been studied in the seminal works of Terrell Hill [2] and Botts and Morales [3], structural mechanisms underlying allostery were first proposed by Monod, Wyman, and Changeux (MWC model; [4]) and by Koshland, Nemethy, and Filmer (KNF model; [5]). The former model posits that a protein undergoes an all-or-none, cooperative transition from a low activity, inactive state to a high activity, active state, with all subunits undergoing the transition together upon ligand binding. Recently, the MWC model has been re-evaluated and reformulated in light of new concepts of allostery. In this revised interpretation, the MWC model considers the inactive state to be an ensemble of conformers, a sub-ensemble of which samples the active state with active state stabilization upon effector binding [6],[7]. In the second model, the KNF model, the protein undergoes a transition consisting of sequential structural rearrangements induced by effector binding, and the inactive state does not adopt an active state in the absence of an effector as in the MWC model.

Over the past 40 years, much has been added to our knowledge of allostery. In particular, the concept of allostery has been extended to single subunit proteins, as allostery was originally characterized in multimeric proteins. In the early 1970s, shortly after the MWC and KNF models were expounded, Neet and coworkers [8] described how hysteretic responses of monomeric proteins to effectors or substrates (a phenomenon first described in detail by Frieden [9]) are correlated with cooperative behavior, a hallmark of allosteric proteins. Since then the presence of allostery in monomeric proteins has been well documented [10]–[13]. Moreover, changes in protein dynamics, in addition to changes in average structure, have been recognized as playing an important role in allostery and protein function in general [10], [14]–[19]. Both NMR experiments and normal mode calculations suggest that the unbound state can adopt conformations resembling a bound state [20]–[22]. This has led to the concept of allosteric proteins existing as an ensemble of conformers, with a binding event shifting the ensemble toward a particular functional state [11]–[13],[23],[24]. Moreover, several studies have documented functionally significant dynamical coupling between residues in allosteric proteins [14],[16],[17], as well as correlations between dynamical coupling and coupling in free energy [17].

Since allostery relies on the communication of binding information from one site to another in a protein, much experimental work has been targeted at elucidating the network of coupled interactions among residues that mediate the allosteric transition. Di Cera and coworkers showed that a network of structural changes connecting an allosteric site and a distant site adjacent to the active site in thrombin is formed upon effector binding to the allosteric site, inducing a key conformational change that renders the active site able to bind substrate [25]. Di Cera and coworkers also demonstrated that a network of hydrogen bonds (some involving waters as well as protein residues) and salt bridges links another key allosteric site, the Na^+^-binding site, to the active site and that this structural network underlies the allosteric transition between so-called slow and fast forms of the enzyme [26]. Work by MacKinnon and coworkers on the voltage-gated potassium channel revealed that residues that affect the gating exist not only in the activation gate and selectivity filter, but also along a path connecting these functional regions, and that these residues are energetically coupled [27]–[29]. Sadovsky and Yifrach further demonstrated that there exist higher order couplings among these residues in addition to pairwise couplings [30]. Recent work on caspase-1 revealed that the network of hydrogen bonds linking the effector and substrate sites changes upon effector binding and that mutation of several residues participating in the network causes reduction in catalytic efficiency [31]. In addition to structurally linked residue networks, residues linked in terms of their dynamic properties have been implicated in allostery. Fuentes et al. showed that the peptide-binding site in a PDZ domain of human tyrosine phosphatase 1E is linked to distant sites via a contiguous network of residues that undergo significant changes in side-chain dynamical properties as measured by NMR [16],[28]. In Pin 1, a peptidyl-prolyl isomerase, a pathway of residues whose side-chains rigidified upon substrate binding linked the active site and the interface between the two domains of the protein [28],[32].

In addition to these experimental studies, computational methods for elucidating the network(s) of coupled interactions among residues have been developed. Lockless and Ranganathan [33] developed a bioinformatic method, statistical coupling analysis (SCA), to discover co-evolved residues in families of proteins, the rationale being that co-conservation reflects a functional coupling. There are also molecular dynamics (MD) techniques aimed at understanding the mechanism by which a signal from one site is transduced to a distant site: pump-probe dynamics [34] and anisotropic thermal diffusion [35]. When these dynamics methods and SCA were applied to a PDZ domain, the three methods yielded similarities in the residues identified as important, but some differences as well, indicating that dynamic and bioinformatic methods can be complementary. Dynamical correlations have been probed using elastic network normal mode analysis combined with a novel method of introducing a theoretical mutation and have yielded significant insight into residue coupling in myosin II [36], helicase [37], and DNA and RNA polymerases [38]. Another method developed originally by Hilser and Freire (COREX; [39]) to study folding pathways, has demonstrated that local order/disorder transitions mediate coupling among distant sites [40]–[42]. Daily et al. [43],[44] investigated coupling among residues by calculating networks of contact rearrangement. Del Sol et al. represented allosteric proteins as graphs of residue van der Waals interactions and showed that residues responsible for maintaining short communication paths correlate with functional significance [45] and that signaling is mediated by residues at the interface between topologically delineated modules [46]. Lastly, Chennubhotla and Bahar [47],[48] developed a method based on Markov propagation of information in a protein, where residues are nodes and inter-residue contacts are edges, to identify sites of high allosteric potential.

Despite providing insight into allosteric regulation, some of these methods have drawbacks. Computational power constraints limit MD-based methods to small systems. While COREX provides significant insights, it uses a reduced model for the degrees of conformational freedom available to a residue, as each residue exists in either a folded or unfolded state. SCA's drawback is that evolutionary co-conservation of residues is not necessarily a property specific to allosterically coupled residues. Finally, the methods of Daily et al. and del Sol et al. rely on single static structures of a protein, and thus lack dynamical measures.

By contrast, a computationally inexpensive meta-method that incorporates a number of parameters putatively implicated in allostery may overcome the drawbacks of individual approaches. In this work, we seek to develop such a method that predicts “hotspot” residues important to allostery for large systems with high sensitivity and specificity. First, we assembled a dataset of residues that were classified as hotspots or non-hotspots (mutations known not to perturb allostery) based on the results of published mutagenesis experiments on allosteric proteins. Then, support vector models were trained to distinguish the known hotspots from the known non-hotspots in this dataset based on several calculated structural, dynamical, network, and informatic features. Support-vector machines are polynomial functions of the calculated features that separate the feature spaces of the hotspots and non-hotspots, thus discriminating between the two classes [49]. An important advantage of SVMs compared with other techniques is that they require no discretization of numerical data, as is the case for machine learning using Bayesian networks. Finally, we also compared our data-mining method with the Statistical Coupling Analysis (SCA) method of Lockless and Ranganathan [33], which has been used to identify networks of allosterically important residues in G-proteins [50],[51] as well as in other families [51],[52].

## Results

### Data Set and Support-Vector Machine Learning

The training data set consisted of point mutants of allosteric enzymes, transcription factors, and signal transduction proteins (Table 1). A residue was designated as a hotspot if a point mutation of that residue reduced cooperativity, as measured by a significant lowering of the Hill coefficient or an increase in IC-50 in the case of negative allostery, or the reduction or abolition of a function that only occurs in the presence of an allosteric effector. Although the most rigorous criterion for classification as a hotspot would be a measure of a residue's perturbation on the allosteric coupling free energy between effector and active site, such a measure is not widely available in the experimental literature. It is thus incumbent upon us to consider other measures that are correlates of free energy coupling. Wyman, after whom the Monod, Wyman, Changeux model of allostery is partly named, has shown that the Hill coefficient is correlated with the coupling free energy between effector and active sites [53] by the following relation (equation 9.4 in his study [53]):
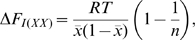
(1)where Δ*F_I(XX)_* is the site-site coupling free energy (*ΔΔG* in our notation), 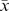 is the fractional saturation of protein with ligand, and *n* is the Hill coefficient. Moreover, a recent study using point mutants to probe the energetic coupling among residues in the voltage-activated potassium channel revealed strong correlations between second- and third order coupling free energies between residues and the associated Hill coefficients (R^2^ = 0.89 and 0.97, respectively) [30]. Based on these results, we assert that perturbation of the Hill coefficient reflects perturbation in site-site coupling free energy. Here, we extrapolate this assertion to other non-energetic measures of site-site coupling, such as IC-50, *in vivo* measures of inducibility (used for transcription factors), and abrogation of the allosteric transtion. Since it has not been shown before, we will make an argument for a relationship between perturbation of the IC-50 of a mutation and the perturbation in site-site coupling free energy. First, we define the coupling free energy between sites as follows in the case where there is a dissociation constant for substrate, *K_S_*, in the absence of allosteric effector and *K_S_'* in the presence of effector, in this case an inhibitor:
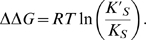
(2)


**Table 1:** Training and Independent Data Sets of Proteins with PDB identifications for the inactive and active state structures for various classes of molecules.

	PDB of effector ligand-unbound (inactive state)	PDB of effector ligand-bound (active state)
**Training Data Set Proteins**		
CheY (signal transduction)	3chy	1fqw
PurR repressor (transcription factor)	1dbq	1wet
Tet repressor (transcription factor)	2trt	1qpi
Hemoglobin (carrier protein/enzyme)	4hhb	1hho
Phosphofructokinase (enzyme)	6pfk	4pfk
phosphoglycerate dehydrogenase (enzyme)	1psd	1yba
fructose-1,6-bisphosphatase (enzyme)	1eyj	1eyi
Aspartate transcarbamoylase (enzyme)	1rac	1d09
RhoA (signal transduction)	1ftn	1a2b
CDC-42 (signal transduction)	1an0	1nf3
glycogen phosphorylase (transcription factor)	1gpb	7gpb
**Independent Data Set Proteins**		
glucokinase (enzyme)	1v4t	1v4s
glutamate dehydrogenase (enzyme)	1nr7	1hwz
lac repressor (transcription factor)	1tlf	1efa
myosin II (motor protein/enzyme)	1vom	1fmw
thrombin (enzyme)	1sgi	1sg8

Since the Michaelis constant, *K_M_*, has been shown to be an apparent dissociation constant, taking into account all substrate-bound species of enzyme, and is directly proportional to *K_S_*, we may replace *K_S_* with *K_M_* in (2) [54]. We then assume mixed inhibition, where the inhibitor can bind to both the substrate-free and substrate-bound states of the enzyme, since this is the most general case of inhibition at a site distinct from the substrate site [55]. The rate equation for mixed inhibition is given in [55] as:
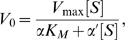
(3)where [*S*] is the concentration of free substrate and *α* is defined as
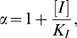
(4)where [*I*] is the inhibitor concentration and *K_I_* is the dissociation constant of inhibitor [55]; *α'* is defined in the same fashion. As the apparent Michaelis constant in the presence of inhibitor is *αK_M_*, the coupling free energy between the inhibitor site and the substrate-binding site after substituting *αK_M_* and *K_M_* for *K_S_'* and *K_S_*, respectively, in (2) is:
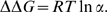
(5)


The Cheng-Prusoff equation relates the *IC-50* to the dissociation constant of inhibitor [56]:
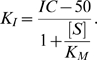
(6)


Substituting (6) for *K_I_* in (4), we have
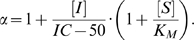
(7)


By substituting (7) in (5), we thus are able to establish a relationship between coupling free energy and *IC-50*. Clearly, a perturbation in this coupling energy due to mutation with an associated coefficient *α_mut_* can be expressed as follows:
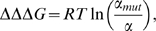
(8)where *α_mut_* reflects an altered IC-50 according to (7).

In the case of allosteric transcription factors, inducibility of the effector was measured using *in vivo* reporter gene assays. Assuming that inducibility is directly related to the differential affinity for DNA in the presence and absence of effector, it can consequently take on an associated coupling free energy, *ΔΔG*. If, in turn, differential expression of a reporter gene is correlated with differential affinity for DNA in the presence and absence of effector, we can establish a link between the reporter gene assay and the coupling free energy.

In this study, care was taken not to include mutations in effector sites, as perturbations in allosteric properties resulting from such mutations could be attributed to altered binding free energy. Also, no mutations were included that completely abolished the protein's function, as such a case could be attributed to perturbed folding of the protein. Hence, our training set was chosen to represent residues that mediate coupling between sites using criteria that are reasonable proxies of energetic coupling. The training data set comprised 44 hotspots and 50 non-hotspots (See Table S1).

Support-vector machine models for predicting allosteric hotspots were initially developed using two sets of features. Feature Set 1 (Table 2) consisted of a combination of dynamical features calculated from normal modes, as well as structural, network, and informatic (primarily sequence-based) features. Feature Set 2 (Table 3) consisted of various structural metrics describing differences between inactive and active state conformations. Please refer to Methods for a complete description of each feature set. All possible combinations of features from Set 1 were tested; however, due to the larger size of Feature Set 2, all possible combinations of features could not be tested. In particular, combinations using between 8 and 14 features at a time could not be tested due to the astronomical number of possible combinations.

**Table 2:** Feature Set 1.

Feature Set 1	Abbreviation
**Dynamical Features**	
Deformation Energy of the inactive state	def-energ-i
Mean Squared Fluctuation of the inactive state	msf-i
Mean Squared Fluctuation of the active state	msf-a
Difference in Mean Squared Fluctuation between inactive and active states	diff-msf
Mutual Information in the inactive state	mut-info-i
**Structural Features**	
B-factor of the inactive state	bfac-i
B-factor of the active state	bfac-a
Difference in B-factor between the inactive and active states	diff-bfac
No. Potential Hydrogen Bonds in the active state	hbond-a
No. Potential Hydrogen Bonds in the inactive state	hbond-i
Difference in No. of Potential Hyd. Bonds between the inactive and active states	diff-hbond
Average Local Atomic Density in the inactive state	at-dens-i
Average Local Atomic Density in the active state	at-dens-a
Difference in Atomic Density between the inactive and active states	diff-at-dens
**Network Features**	
Node degree in inactive state	node-deg-i
Perturbation in Clustering Coefficient upon Node Removal in inactive state	pert-clust-coef-i
**Informatic Features**	
Evolutionary Conservation	cons
Local Structural Entropy	lse

**Table 3:** Feature Set 2.

Feature Set 2	Abbreviation
Alpha-carbon Displacement	Ca-disp
Side-Chain RMS Distance between inactive and active states	sc-rms
Rotation of Alpha Carbon-Beta Carbon bond from the inactive to active state	sc-flip
Difference in Phi Angle between inactive state and active states	dphi
Difference in Psi Angle between inactive state and active states	dpsi
Maximum of dphi and dpsi	maxdihed
Difference in Chi1 Angle between inactive state and active states	dchi1
Difference in Chi2 Angle between inactive state and active states	dchi2
Maximum of dchi1 and dchi2	maxdchi
Fractional Change in Contact Environment relative to inactive state	fI
Fractional Change in Contact Environment relative to active state	fA
Maximum of fI and fA	fmax
All-Atom Solvent-Accessible Surface Area in inactive state	asa1
All-Atom Solvent-Accessible Surface Area in active state	asa2
Average of asa1 and asa2	asaavg
Side-Chain Solvent-Accessible Surface Area in inactive state	asasc1
Side-Chain Solvent-Accessible Surface Area in active state	asasc2
Average of asasc1 and asasc2	asascavg
Backbone Solvent-Accessible Surface Area in inactive state	asabb1
Backbone Solvent-Accessible Surface Area in active state	asabb2
Average of asabb1 and asabb2	asabbavg

Both second- and third-degree polynomial kernels were used in the training. In the context of SVMs, the kernel is the following expression:
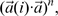
(9)where 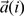 is the ith support vector defining the optimal hyperplane (often referred to as the maximal margin hyperplane) separating two classes (here, hotspot and non-hotspot); 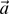 is a test instance; and *n* is an integer representing the degree of the kernel [57]. The complete function that is at the core of the SVM algorithm is as follows:
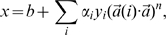
(10)where *y_i_* is the class corresponding to support vector 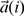, and *b* and *α_I_* are parameters to be determined. Linear (first-degree) kernels were tried initially, but they performed less well in preliminary tests than the other two kernels tested. Higher order polynomials were not tried, as their use can result in overfitting of the models to the data due to the greater number of parameters involved, particularly in situations like ours where the size of the training data set is relatively small.

### Results for Cross-Validation Training/Testing

Each feature/kernel degree combination tested in the training was evaluated for predictive performance. For each combination, a nine-fold cross-validation was performed, where a model was trained on 8 portions of the training data and tested on the ninth. Here, each portions consists of one protein's hotspots and non-hotspots, except in cases for which only hotspots or non-hotspots existed in the data set. For these cases, the hotspots of a protein without non-hotspots were grouped with the non-hotspots of a protein having no hotspots. Thus, each feature/kernel degree combination resulted in nine support-vector-machine models. This procedure is designed to prevent overtraining, or over-fitting, of the support-vector machine parameters, which results from training on all the data at once and yields inflated performance measures. Precision, recall, and F1 were calculated for each feature/kernel combination by pooling the true positives, false positives, true negatives, and false negatives from each of the nine folds (models). Each feature/kernel degree combination was ranked by F1. The top 20 feature/kernel degree combinations by F1 using Feature Set 1 are given in Table 4. Using Feature Set 1, precision ranged from 0.54–0.64, recall from 0.68–0.91, and F1 from 0.66–0.68 for the top 20 feature/kernel degree combinations. A similar range in performance was seen in the top 300 feature/kernel degree combinations, where precision ranged from 0.51–0.66, recall from 0.61–0.91, and F1 from 0.63–0.68 (Table 5).

**Table 4:** Top 20 highest performing feature/kernel degree combinations (as ranked by F1) using Feature Set 1.

F1	Precision	Recall	Feature Combination	Kernel Degree
0.68	0.62	0.75	def-energ-i, msf-i, diff-msf, at-dens-a, diff-at-dens, diff-bfac, lse	3
0.68	0.58	0.82	msf-i, msf-a, diff-hbond, bfac-a, node-deg-i, lse	2
0.68	0.54	0.91	msf-i, msf-a, diff-hbond	3
0.67	0.63	0.73	def-energ-i, msf-i, diff-msf, at-dens-i, at-dens-a, diff-at-dens, diff-bfac, lse	3
0.67	0.61	0.75	msf-a, diff-hbond, diff-at-dens, bfac-a, lse	2
0.67	0.60	0.77	msf-i, msf-a, mut-info-i, diff-hbond, diff-at-dens, bfac-a, lse	2
0.67	0.57	0.82	msf-i, diff-hbond, node-deg-i, lse	3
0.67	0.57	0.82	msf-i, msf-a, hbond-i, diff-hbond, bfac-a, lse	2
0.67	0.57	0.82	def-energ-i, msf-i, diff-hbond, lse	3
0.67	0.62	0.73	msf-a, diff-msf, diff-hbond, at-dens-a, diff-at-dens, bfac-a, lse	2
0.67	0.56	0.82	msf-i, msf-a, diff-hbond, diff-bfac, lse	3
0.66	0.56	0.80	def-energ-i, msf-i, diff-hbond, diff-at-dens, diff-bfac, lse	3
0.66	0.56	0.80	def-energ-i, msf-i, msf-a, diff-msf, diff-hbond, diff-at-dens, lse	3
0.66	0.58	0.77	msf-i, hbond-i, diff-hbond, node-deg-i, lse	2
0.66	0.58	0.77	def-energ-i, msf-i, diff-hbond, lse	2
0.66	0.59	0.75	def-energ-i, msf-a, diff-hbond, diff-at-dens, diff-bfac, lse	3
0.66	0.60	0.73	def-energ-i, msf-a, diff-hbond, diff-at-dens, bfac-a, node-deg-i, lse	2
0.66	0.62	0.70	def-energ-i, msf-a, diff-msf, diff-hbond, diff-at-dens, diff-bfac, node-deg-i, lse	3
0.66	0.62	0.70	def-energ-i, msf-i, diff-msf, diff-hbond, at-dens-a, diff-at-dens, diff-bfac, lse	3
0.66	0.64	0.68	def-energ-i, msf-a, diff-hbond, diff-at-dens, diff-bfac, node-deg-i, lse	3

Precision, recall, and F1 scores calculated from the results of the nine-fold cross-validation on the training set. Refer to Table 2 for explanations of feature abbreviations.

**Table 5:** Summary of the performance of the four feature sets.

Feature Set	Range of F1 of top 300 models for training data set	No. of models of top 300 w/F1>0.60 on ind. data set	F1 of top model on ind. data set
Feature Set 1	0.63–0.68	22	0.73
Feature Set 2	0.68–0.71	293	0.68
Aug. Feature Set 1	0.60–0.71	31	0.68
Hybrid Feature Set	0.63–0.73**	26,113**	0.73

**: 80,000 feature/kernel degree combinations using the Hybrid Feature Set had F1 scores in the range of 0.63–0.73 on the training data set, and all of these feature/kernel degree combinations were tested on the independent data set. 26,113 models of the 80,000 had an F1 greater than 0.60 on the independent data set. Abbreviation: ind. = independent.

For the top 300 feature/kernel degree combinations using Set 2, precision was lower compared with Feature Set 1 (0.53–0.61; p = 4.2e-10), but recall (0.80–0.95; p<2.2e-16) and F1 (0.68–0.71; p<2.2e-16) were higher (Table 5). The top 20 feature/kernel degree combinations using Feature Set 2 are given in Table 6. Again, the performance of the top 20 feature/kernel degree combinations of this feature set was similar to the top 300, with precision ranging from 0.55–0.61, recall from 0.84–0.95, and F1 from 0.70–0.71.

**Table 6:** Top 20 highest performing feature/kernel degree combinations (as ranked by F1) using Feature Set 2.

F1	Precision	Recall	Feature Combination	Kernel Degree
0.71	0.58	0.93	Ca-disp, sc-flip, asa1, asa2, asasc1	3
0.71	0.58	0.93	Ca-disp, sc-flip, asa1, asa2, asasc1	3
0.71	0.58	0.91	dpsi, asaavg, asascavg, asabbavg	3
0.71	0.56	0.95	Ca-disp, sc-flip, asa1, asa2, asasc1, asascavg	3
0.70	0.61	0.84	dpsi, dchi1, asascavg	2
0.70	0.57	0.91	maxdchi, asa2, asasc1, asascavg, asabb1, asabbavg	2
0.70	0.57	0.91	maxdchi, asa2, asaavg, asasc1, asabb1, asabbavg	2
0.70	0.57	0.91	maxdchi, asa1, asaavg, asasc2, asabbavg	2
0.70	0.57	0.91	maxdchi, asa1, asa2, asasc1, asascavg, asabbavg	2
0.70	0.57	0.91	maxdchi, asa1, asa2, asasc1, asascavg, asabb1, asabbavg	2
0.70	0.57	0.91	maxdchi, asa1, asa2, asasc1, asasc2, asabbavg	2
0.70	0.57	0.91	maxdchi, asa1, asa2, asasc1, asasc2, asascavg, asabbavg	2
0.70	0.56	0.93	sc-flip, asa2, asasc1, asascavg, asabb1, asabbavg	3
0.70	0.56	0.93	asa2, asaavg, asasc2, asabb1	3
0.70	0.56	0.93	Ca-disp, sc-flip, dchi2, asa1, asa2, asaavg	3
0.70	0.56	0.93	Ca-disp, sc-flip, asa2, asaavg, asasc1	2
0.70	0.56	0.93	Ca-disp, sc-flip, asa1, asa2, asaavg, asascavg	3
0.70	0.55	0.95	Ca-disp, sc-flip, asa2, asaavg, asasc1	3
0.70	0.55	0.95	Ca-disp, sc-flip, asa2, asaavg, asasc1	3
0.70	0.58	0.86	dpsi, dchi1, asa1, asasc2, asabb1	2

Precision, recall, and F1 scores calculated from the results of the nine-fold cross-validation on the training set. Refer to Table 3 for explanations of feature abbreviations.

**Figure 1:**
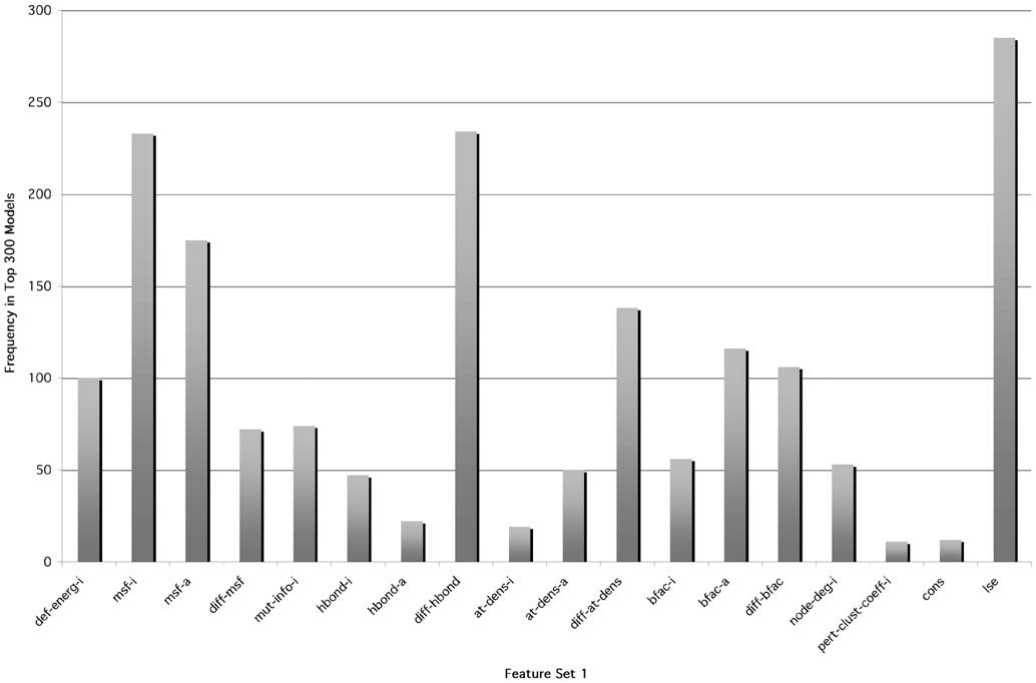
Feature usage in the top 300 SVM models using Feature Set 1. For each feature, the number of models (frequency) in the top 300, as ranked by F1 performance on the training data, that used that particular feature was tabulated.

**Figure 2:**
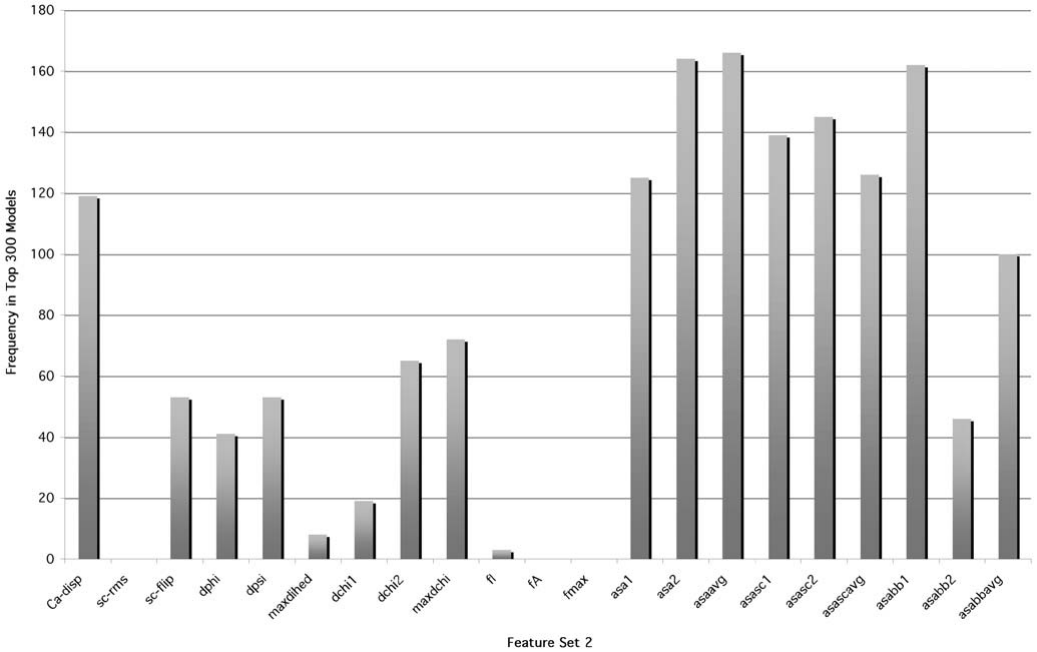
Feature usage in the top 300 SVM models using Feature Set 2. For each feature, the number of models (frequency) in the top 300, as ranked by F1 performance on the training data, that used that particular feature was tabulated.

Identifying the features that were used most frequently in the top 300 feature/kernel degree combinations can yield insights into properties that may, when taken together, indicate signatures of an allosteric hotspot residue. Dominant features in the top 300 feature combinations of Set 1 were mean squared fluctuation in the inactive and active conformers; difference in atomic density between inactive and active conformers; deformation energy of the inactive state; difference in the number of hydrogen bonds between inactive and active states; B-factor in the active state; difference in B-factor between the inactive and active states; and local structural entropy (Figure 1). Features from Set 2 that were dominant when considering the top 300 different combinations were as follows: alpha-carbon displacement; total residue solvent-accessible surface area in the inactive and active states, and the average of the two; side-chain solvent-accessible surface area in both states, and the average; backbone solvent-accessible surface area in the active state, and the average of this value in the inactive and active states (Figure 2).

Since many allosteric proteins have only a single solved structure on which to base hotspot predictions, we identified feature/kernel degree combinations in the top 300 from the Set 1 analysis consisting solely of features calculated from a single structure (either the inactive or active state) or a single structure plus sequence-based features. Seventeen such combinations exist for which precision, recall, and F1 ranges were 0.53–0.56, 0.73–0.80, and 0.63–0.65, respectively (Table 7). Strikingly, in all of these 17 combinations, the single structure required is the inactive structure. No feature/kernel degree combinations in the top 300 required only the active state structure.

**Table 7:** Feature/kernel degree combinations from the top 300 models which used only sequence or inactive state structural information.

F1	Precision	Recall	Feature Combination	Kernel Degree
0.65	0.56	0.80	msf-i, lse	3
0.65	0.55	0.80	msf-i, lse, mut-info-i	3
0.65	0.55	0.80	msf-i, hbond-i, lse	3
0.65	0.56	0.77	msf-i, lse, mut-info-i	2
0.65	0.56	0.77	msf-i, lse, node-deg-i	3
0.64	0.56	0.75	msf-i, bfac-i, lse, node-deg-i	2
0.64	0.56	0.75	def-energ-i, msf-i, lse, mut-info-i	2
0.63	0.55	0.75	msf-i, lse, node-deg-i, mut-info-i	3
0.63	0.55	0.75	msf-i, lse	2
0.63	0.55	0.75	msf-i, bfac-i, hbond-i, lse	2
0.63	0.56	0.73	msf-i, hbond-i, lse, node-deg-i	3
0.63	0.56	0.73	def-energ-i, msf-i, lse	3
0.63	0.56	0.73	def-energ-i, msf-i, lse	2
0.63	0.53	0.77	msf-i, bfac-i, lse, mut-info-i	3
0.63	0.53	0.77	msf-i, bfac-i, mut-info-i	3
0.63	0.54	0.75	msf-i, bfac-i, lse, mut-info-i	2
0.63	0.54	0.75	def-energ-i, msf-i, hbond-i, lse	3

Precision, recall, and F1 scores calculated from the results of the nine-fold cross-validation on the training set.

To ascertain whether there are general discrepancies in the predictive behavior of models generated with Feature Set 1 and 2, we assessed the overlap in the predictions between the two feature sets. Here, we considered how many predictions were the same using a pair of feature/kernel degree combinations and how many were different, where one combination was based on Feature Set 1 and the other on Feature Set 2. All pair-wise combinations of models were tested. On average, the models agreed on 61.4% of their predictions and disagreed 38.6% of the time.

We hypothesized that residues important for allostery may reside in hinge regions that undergo a change in their deformation properties upon binding an allosteric effector. To test this, we assessed whether adding features related to active state deformations would result in more accurate models. We augmented the top 8 features as ranked by their frequency in the top 300 feature/kernel combinations (deformation energy in the inactive state; mean-squared fluctuation in the inactive and active states; difference in the number of H-bonds between inactive and active states; local structural entropy; difference in atomic density between inactive and active states; B-factor in the active state; and the difference in B-factor between the inactive and active states) with the deformation energy of the active state and the difference in the deformation energy between inactive and active states. We then evaluated all possible combinations of those features using kernel degree 2 or 3 and cross-validation on the training data set. The top 20 scoring feature/kernel degree combinations using this feature set (referred to henceforth as Augmented Feature Set 1) scored about 2 points higher in F1 than Feature Set 1 (Table 8), and nearly all of these made use of the additional active state features. For the top 300 models, the F1 ranged from 0.60–0.71 (Table 5).

**Table 8:** Top 20 highest performing feature/kernel degree combinations (as ranked by F1) using top 8 Set 1 features augmented with deformation energy of the active state (abbreviated def-energ-r in the table) and the difference in deformation energy between the inactive and active states (abbreviated diff-def-energ), Augmented Feature Set 1.

F1	Precision	Recall	Feature Combination	Kernel Degree
0.71	0.64	0.80	msf-i, diff-hbond, lse, diff-at-dens, msf-a, bfac-a, def-energ-a	2
0.70	0.66	0.75	diff-hbond, lse, diff-at-dens, msf-a, def-energ-a	3
0.70	0.64	0.77	msf-i, diff-hbond, lse, diff-at-dens, diff-bfac, def-energ-a	3
0.69	0.63	0.77	diff-hbond, lse, diff-at-dens, msf-a, bfac-a, def-energ-a	3
0.69	0.61	0.80	msf-i, diff-hbond, lse, diff-at-dens, msf-a, bfac-a, diff-bfac, def-energ-a	2
0.69	0.63	0.75	def-energ-i, diff-hbond, lse, diff-at-dens, msf-a, bfac-a, def-energ-a	2
0.69	0.63	0.75	def-energ-i, diff-hbond, lse, diff-at-dens, msf-a, bfac-a, diff-def-energ	2
0.69	0.59	0.82	diff-hbond, lse, msf-a, diff-def-energ	3
0.69	0.58	0.84	def-energ-i, msf-i, lse, diff-def-energ	3
0.68	0.64	0.73	diff-hbond, lse, diff-at-dens, msf-a, bfac-a, def-energ-a, diff-def-energ	2
0.68	0.62	0.75	diff-hbond, lse, diff-at-dens, msf-a, bfac-a, def-energ-a	2
0.68	0.62	0.75	def-energ-i, diff-hbond, lse, diff-at-dens, msf-a, bfac-a	2
0.68	0.62	0.75	def-energ-i, msf-i, diff-hbond, lse, diff-at-dens, msf-a, diff-bfac, def-energ-a	3
0.68	0.61	0.77	msf-i, diff-hbond, lse, diff-at-dens, msf-a, diff-bfac, def-energ-a	3
0.68	0.54	0.91	msf-i, diff-hbond, msf-a	3
0.67	0.63	0.73	def-energ-i, diff-hbond, lse, diff-at-dens, msf-a, diff-def-energ	3
0.67	0.63	0.73	def-energ-i, msf-i, diff-hbond, lse, diff-at-dens, msf-a, bfac-a, def-energ-a	2
0.67	0.61	0.75	diff-hbond, lse, diff-at-dens, msf-a, bfac-a	2
0.67	0.61	0.75	def-energ-i, diff-hbond, lse, diff-at-dens, msf-a, diff-bfac	3

Precision, recall, and F1 scores calculated from the results of the nine-fold cross-validation on the training set.

### Results for Testing on the Independent Data Set

We tested our top models on an independent data set to further assess their predictive performance, because a useful predictive model should perform well on data that is unseen during the training. The top 300 feature/kernel degree combinations of Set 1 and Set 2 were used to train support-vector models. Here we created a single support-vector model by training on the entire training data set at once (rather than doing a cross-validation as in the previous section, where each fold in the training generated a model), and then tested this model on an independent data set. The independent data set consisted of 87 experimentally determined hotspots and non-hotspots from five allosteric proteins (Table 1). 22 of the top 300 Feature Set 1-models had an F1 ranging from 0.60–0.73 (Table 5; See Table 9 for the top 20 models). 293 of the top 300 models (Table 5) for Feature Set 2 had an F1 that ranged from 0.60–0.68 (See Table 10 for the top 20 models.). Although Feature Set 2 had higher F1 scores than Set 1 on the independent data set (p = 7.7e-5), the top 20 highest scoring Set 1 models on the independent data set were more precise (p = 1.0e-8) than the corresponding top 20 Set 2 models (Refer to Table 9 and 10). For Feature Set 1, mean square fluctuation, hydrogen bonding, and atomic density predominated in the top 20 models. For Feature Set 2, the dominant features for the top models related to solvent-accessible surface area and alpha-carbon displacement, similar to the results for the cross-validated training/testing.

**Table 9:** Performance of the top Feature Set 1-models on the independent data set.

F1	Precision	Recall	Feature Combination	Kernel Degree
0.73	0.67	0.81	msf-i, diff-hbond, mut-info-i, msf-a, diff-msf	3
0.68	0.60	0.78	msf-i, mut-info-i, msf-a	3
0.68	0.59	0.81	msf-i, diff-hbond, mut-info-i, msf-a	3
0.67	0.61	0.76	msf-i, diff-hbond, msf-a, diff-msf	3
0.67	0.61	0.73	msf-i, hbond-a, msf-a	3
0.67	0.58	0.78	msf-i, diff-hbond, diff-at-dens, mut-info-i, msf-a	3
0.66	0.58	0.76	msf-i, diff-hbond, msf-a	3
0.66	0.58	0.76	msf-i, bfac-i, msf-a	3
0.66	0.62	0.70	msf-i, msf-a, diff-msf	3
0.66	0.64	0.68	msf-i, diff-hbond, msf-a	2
0.66	0.64	0.68	msf-i, diff-hbond, diff-at-dens, msf-a	2
0.66	0.70	0.62	hbond-a, hbond-i, lse, mut-info-i, msf-a, diff-msf	3
0.65	0.63	0.68	msf-i, bfac-i, diff-hbond, msf-a	3
0.64	0.57	0.73	msf-i, diff-hbond, diff-at-dens, msf-a	3
0.64	0.61	0.68	def-eng-i, msf-i, diff-hbond, msf-a	3
0.63	0.67	0.59	hbond-a, hbond-i, lse, msf-a, diff-msf	3
0.62	0.68	0.57	hbond-a, hbond-i, lse, diff-at-dens, msf-a, diff-msf	3
0.61	0.61	0.62	hbond-i, lse, diff-at-dens, msf-a, diff-msf	3
0.60	0.61	0.59	diff-hbond, lse, diff-at-dens, pert-clust-coeff-i, msf-a, bfac-a	2
0.60	0.61	0.59	msf-i, diff-hbond, lse, at-dens-a, diff-at-dens, pert-clust-coeff-i, msf-a, bfac-a	2

Each of the top 300 feature/kernel degree combinations (as determined by the leave-one-out cross-validation) was used to train a model on the entire training data set. The resulting models were tested on the independent data set.

**Table 10:** Performance of top Feature Set 2-models on the independent data set.

F1	Precision	Recall	Feature Combination	Kernel Degree
0.69	0.56	0.89	Ca-disp, dchi2, asa1, asaavg, asasc1, asabb1	3
0.69	0.56	0.89	Ca-disp, dpsi, dchi2, asaavg, asascavg, asabb1	3
0.69	0.56	0.89	Ca-disp, asa1, asaavg, asasc1, asabb1, asabbavg	3
0.69	0.55	0.92	dpsi, asaavg, asascavg	3
0.68	0.59	0.81	sc-flip, asa2, asaavg, asasc2, asascavg, asabb1	3
0.67	0.58	0.81	Ca-disp, sc-flip, dchi1, dchi2, asasc2, asascavg, asabb1	2
0.67	0.55	0.86	dpsi, dchi1, asascavg	2
0.67	0.55	0.86	Ca-disp, sc-flip, dchi2, asa1, asaavg, asasc2	3
0.67	0.55	0.86	Ca-disp, asa1, asasc1, asascavg, asabbavg	2
0.67	0.55	0.86	Ca-disp, fI, asaavg, asasc1, asascavg, asabbavg	2
0.67	0.54	0.89	Ca-disp, dpsi, asa1, asa2	3
0.67	0.54	0.89	Ca-disp, asa1, asaavg, asasc1	2
0.67	0.57	0.81	dchi1, dchi2, asasc2, asascavg, asabb1	2
0.67	0.55	0.84	dpsi, dchi2, asasc1, asascavg, asabb1, asabb2	3
0.67	0.54	0.86	dpsi, dchi2, asaavg	3
0.67	0.54	0.86	dpsi, asaavg, asascavg, asabbavg	3
0.67	0.54	0.86	dpsi, asa2, asaavg, asascavg, asabb1, asabb2	3
0.67	0.54	0.86	dpsi, asa1, asa2, asaavg, asasc1, asabb1	3
0.67	0.54	0.86	Ca-disp, dchi2, asaavg, asascavg	2
0.67	0.54	0.86	Ca-disp, dpsi, dchi2, asa2, asascavg, asabb1	3

Each of the top 300 feature/kernel degree combinations (as determined by the leave-one-out cross-validation) was used to train a model on the entire training data set. The resulting models were tested on the independent data set. The top 20 models are given above.

We also evaluated the performance of the top 300 inactive state- and/or sequence-based (i.e., single structure-based) Feature Set 1 models on the independent data (Table 11). These models had an F1 in the range of 0.49–0.56, and all except one yielded a precision greater than or equal 0.53. Thus, although the models based on inactive state and sequence may not have had high F1 or recall, the high precision is noteworthy. In cases where only an inactive state structure is known, this provides the experimentalist with a good starting point for further exploration of the computational predictions.

**Table 11:** Performance of models that used only inactive state structure and/or sequence information from the top 300 on the independent data set.

F1	Precision	Recall	Feature Combination	Kernel Degree
0.56	0.54	0.59	msf-i, lse	3
0.56	0.54	0.59	def-eng-i, msf-i, lse	3
0.55	0.54	0.57	msf-i, hbond-i, lse	3
0.55	0.56	0.54	msf-i, lse, node-deg-i, mut-info-i	3
0.55	0.56	0.54	msf-i, bfac-i, hbond-i, lse	2
0.55	0.53	0.57	msf-i, lse, mut-info-i	3
0.55	0.53	0.57	msf-i, bfac-i, lse	3
0.54	0.54	0.54	msf-i, lse, node-deg-i	3
0.54	0.54	0.54	msf-i, lse	2
0.53	0.53	0.54	msf-i, bfac-i, lse, mut-info-i	3
0.53	0.51	0.54	def-eng-i, msf-i, hbond-i, lse	3
0.52	0.53	0.51	msf-i, lse, mut-info-i	2
0.52	0.53	0.51	msf-i, bfac-i, lse, mut-info-i	2
0.51	0.57	0.46	def-eng-i, msf-i, lse, mut-info-i	2
0.50	0.55	0.46	msf-i, bfac-i, lse, node-deg-i	2
0.49	0.53	0.46	def-eng-i, msf-i, lse	2
0.49	0.57	0.43	msf-i, hbond-i, lse, node-deg-i	3

Further, the performance of the top 300 models using Augmented Feature Set 1 on the independent data was evaluated. The performance of the top 20 highest scoring models is given in Table 12. 31 models of the top 300 scored an F1 of 0.60–0.68 on the independent data set (Table 5). Similar to Feature Set 1, although the F1 scores of the top 20 highest scoring models using this feature set were lower than those of Set 2 (2.2e-5), precision scores were significantly higher than those of Set 2 (p = 4.8e-12).

**Table 12:** Performance of the top 20 models consisting of the top 8 features from Set 1 augmented with deformation energy of the active state (abbreviated def-energ-a in the table) and the difference in deformation energy between the inactive and active states (abbreviated diff-def-energ) on the independent data set (Augemented Feature Set 1).

F1	Precision	Recall	Feature Combination	Kernel Degree
0.68	0.67	0.70	def-energ-i, msf-i, diff-at-dens, msf-a, def-energ-a	2
0.68	0.68	0.68	def-energ-i, msf-i, diff-hbond, msf-a	2
0.68	0.63	0.73	msf-i, diff-hbond, msf-a, diff-def-energ	3
0.67	0.66	0.68	msf-i, diff-at-dens, msf-a, diff-def-energ	2
0.67	0.66	0.68	msf-i, diff-hbond, msf-a, def-energ-a	2
0.67	0.63	0.70	msf-i, msf-a, def-energ-a	2
0.66	0.58	0.76	msf-i, diff-hbond, msf-a	3
0.66	0.62	0.70	msf-i, diff-hbond, msf-a, def-energ-a	3
0.66	0.64	0.68	msf-i, diff-hbond, msf-a	2
0.66	0.64	0.68	msf-i, diff-hbond, diff-at-dens, msf-a	2
0.66	0.64	0.68	msf-i, diff-hbond, diff-at-dens, msf-a, def-energ-a	2
0.66	0.67	0.65	def-energ-i, msf-i, diff-hbond, diff-at-dens, msf-a	2
0.65	0.65	0.65	msf-i, msf-a, diff-def-energ	2
0.65	0.65	0.65	msf-i, diff-at-dens, msf-a, def-energ-a, diff-def-energ	2
0.64	0.57	0.73	msf-i, diff-hbond, diff-at-dens, msf-a	3
0.64	0.66	0.62	def-energ-i, lse, msf-a, def-energ-a, diff-def-energ	3
0.62	0.62	0.62	msf-i, diff-hbond, lse, msf-a, def-energ-a	3
0.62	0.59	0.65	def-energ-i, msf-i, diff-hbond, msf-a	3
0.61	0.61	0.62	def-energ-i, msf-i, lse, diff-at-dens, diff-def-energ	3
0.61	0.63	0.59	msf-i, diff-hbond, diff-at-dens, msf-a, diff-def-energ	2

Precision, recall, and F1 scores were calculated from the results on the independent data set.

### Parsimony of Feature Usage/Feature Enrichment in Feature Set 1 SVM Models

**Figure 3:**
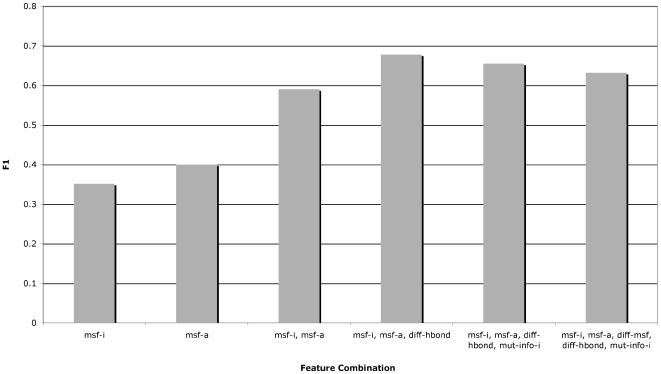
Improvement of F1 upon successive feature addition. The bar on the far right represents a feature combination from the top 10 models. Preceding bars represent feature combinations where each bar contains one feature fewer than the bar to its right.

To assess how much each feature contributes to the predictive ability of a given feature/kernel degree combination, we considered a feature combination from the top 300 that also performed well on the independent data set and analyzed the effect of successive feature addition. In this analysis, the starting point is one feature contained in a top-300 feature/kernel degree combination, followed by a 2-feature model, etc. (Figure 3). The greatest improvement in F1 occurred with the combination of two features (mean-squared fluctuation in the active and inactive states), followed by a modest improvement after the addition of some third feature. Additional features did not appreciably improve the F1 scores. This suggests that mean-squared fluctuations in the two states are “anchor” features for this particular model, and successive features finely tune the performance.

Naturally, a parsimonious model that makes accurate predictions with fewer parameters (or features, in our case) is more favorable than one that requires a large number of features. Having fewer features reduces the number of required calculations for test cases, and lowers propensity for overfitting. Thus, we investigated whether any of the top 300 feature/kernel degree combinations consisted of just 2 or 3 features. Twenty-three such feature/kernel degree combinations were found within the top 300. Feature usage in these combinations reflected that of the top 300 feature/kernel degree combinations, with mean-squared fluctuation and local structural entropy predominating (Table 13).

**Table 13:** Feature/kernel degree combinations from the top 300 models that used only two or three features.

F1	Precision	Recall	Feature Combination	Kernel Degree
0.68	0.54	0.91	msf-i, diff-hbond, msf-a	3
0.65	0.55	0.82	msf-i, diff-hbond, msf-a	2
0.65	0.56	0.80	msf-i, lse	3
0.65	0.54	0.82	msf-i, msf-a, diff-msf	3
0.65	0.55	0.80	msf-i, lse, diff-msf	3
0.65	0.55	0.80	msf-i, lse, mut-info-i	3
0.65	0.55	0.80	msf-i, diff-hbond, lse	3
0.65	0.55	0.80	msf-i, hbond-i, lse	3
0.65	0.56	0.77	msf-i, lse, mut-info-i	2
0.65	0.56	0.77	msf-i, lse, node-deg-i	3
0.64	0.54	0.80	msf-i, hbond-a, msf-a	3
0.64	0.55	0.77	msf-i, lse, diff-bfac	3
0.64	0.55	0.77	msf-i, diff-hbond, lse	2
0.64	0.55	0.77	msf-i, hbond-a, lse	3
0.64	0.52	0.82	msf-i, mut-info-i, msf-a	3
0.63	0.55	0.75	msf-i, lse	2
0.63	0.56	0.73	msf-i, lse, at-dens-a	3
0.63	0.56	0.73	def-energ-i, msf-i, lse	3
0.63	0.56	0.73	def-energ-i, msf-i, lse	2
0.63	0.53	0.77	msf-i, lse, diff-at-dens	3
0.63	0.53	0.77	msf-i, bfac-i, lse	3
0.63	0.54	0.75	msf-i, hbond-i, msf-a	2
0.63	0.54	0.75	msf-i, bfac-i, msf-a	3

Precision, recall, and F1 scores calculated from the results of the nine-fold cross-validation on the training set.

### Combining Feature Set 1 and Set 2

Because each feature set had its unique strengths in terms of predictive power, and there was limited consensus of predictions between models using the two feature sets, we formed a hybrid feature set consisting of the features of Set 1 and 2 that were most prevalent in top models. Specifically, we pooled the top 8 features from Set 1 as ranked by frequency in the top 300 feature/kernel degree combinations trained solely on this feature set (deformation energy in the inactive state; mean-squared fluctuation in the inactive and active states; difference in the number of H-bonds between inactive and active states; local structural entropy; difference in atomic density between inactive and active states; B-factor in the active state; and the difference in B-factor between the inactive and active states – see Figure 1) and the top 9 features from Set 2 as ranked in the same fashion (α-carbon displacement; percent all-atom SASA in inactive and active states, and the average of the two; percent side chain SASA in inactive and active states, and the average of the two; percent backbone SASA in the inactive state and the average of this measure in the inactive and active states – see Figure 2). We then trained hybrid models using all possible combinations of this mixture of the top Set 1 and 2 features (Hybrid Feature Set). The Hybrid Feature Set yielded the highest scoring feature/kernel degree combinations in terms of F1 on the training data set (Table 14; p<2.2e-16, when comparing F1 of the top 300 Hybrid Feature Set-feature/kernel degree combinations with those of Set 2, the one that performed the best on the training data). For the top 300 feature/kernel degree combinations, F1 ranged from 0.71–0.73, precision ranged from 0.56–0.70 (296 of these had precision greater than 0.60), and recall ranged from 0.73–0.93. 80,000 feature/kernel degree combinations had an F1 score greater than or equal to 0.63 in the cross-validation on the training data (Table 5). Thus, the hybrid set produces a much higher proportion of good- to excellent-quality models than either feature set 1 or 2 alone. These 80,000 were then used for training on the entire training data set, and the resulting models were tested on the independent data set.

**Table 14:** Top 20 highest performing feature/kernel degree combinations (as ranked by F1) using all possible combinations of a mixture of Set 1 and Set 2 features that were found most frequently in the top-scoring models made using all possible combinations of each of the two feature sets separately (Hybrid Feature Set).

F1	Precision	Recall	Feature Combination	Kernel Degree
0.73	0.65	0.84	def-energ-i, msf-i, diff-hbond, lse, diff-at-dens, bfac-a, diff-bfac, asa2, asaavg, asasc1, asascavg, asabb1	3
0.73	0.65	0.84	def-energ-i, msf-i, diff-hbond, lse, diff-at-dens, bfac-a, diff-bfac, asa2, asaavg, asasc1, asascavg, asabb1	3
0.72	0.68	0.77	def-energ-i, diff-hbond, lse, bfac-a, Ca-disp, asasc1, asasc2, asascavg, asabb1, asabbavg	2
0.72	0.68	0.77	def-energ-i, diff-hbond, lse, bfac-a, Ca-disp, asa2, asaavg, asasc2	2
0.72	0.66	0.80	def-energ-i, diff-hbond, lse, diff-at-dens, diff-bfac, asascavg	3
0.72	0.66	0.80	def-energ-i, msf-i, lse, diff-at-dens, bfac-a, diff-bfac, asa1, asa2, asasc1, asasc2, asabb1	3
0.72	0.64	0.82	def-energ-i, msf-i, lse, diff-at-dens, bfac-a, diff-bfac, asa1, asa2, asasc1, asascavg, asabb1	3
0.72	0.64	0.82	def-energ-i, msf-i, diff-hbond, lse, diff-at-dens, bfac-a, diff-bfac, asa2, asaavg, asasc1, asasc2, asascavg, asabb1	3
0.72	0.64	0.82	def-energ-i, msf-i, diff-hbond, lse, diff-at-dens, bfac-a, diff-bfac, asa1, asaavg, asasc1, asasc2, asabbavg	3
0.72	0.64	0.82	def-energ-i, msf-i, diff-hbond, lse, diff-at-dens, bfac-a, diff-bfac, asa1, asaavg, asasc1, asasc2, asabbavg	3
0.72	0.64	0.82	def-energ-i, msf-i, diff-hbond, lse, diff-at-dens, bfac-a, diff-bfac, asa1, asa2, asasc1, asascavg, asabbavg	3
0.72	0.64	0.82	def-energ-i, msf-i, diff-hbond, lse, diff-at-dens, bfac-a, diff-bfac, asa1, asa2, asasc1, asascavg, asabbavg	3
0.72	0.69	0.75	def-energ-i, diff-hbond, lse, bfac-a, asasc1, asasc2, asascavg	2
0.72	0.61	0.86	def-energ-i, lse, Ca-disp, asasc2, asabb1, asabbavg	3
0.72	0.61	0.86	def-energ-i, diff-hbond, lse, asasc2	3
0.72	0.67	0.77	def-energ-i, diff-hbond, lse, bfac-a, Ca-disp, asa1, asa2, asasc1, asasc2, asabbavg	2
0.72	0.67	0.77	def-energ-i, diff-hbond, lse, bfac-a, Ca-disp, asa1, asa2, asaavg, asasc2, asascavg, asabbavg	2
0.72	0.67	0.77	def-energ-i, diff-hbond, lse, bfac-a, diff-bfac, asa2, asaavg, asasc2	2
0.72	0.67	0.77	def-energ-i, diff-hbond, lse, bfac-a, asasc2, asascavg, asabbavg	2

Precision, recall, and F1 scores were calculated from the results of the nine-fold cross-validation on the training set.

The models that scored highest on the independent data set are listed in Table 15. 26,113 models of the 80,000 had an F1 score greater than or equal to 0.60 on the independent data set (Table 5). F1 scores on the independent data set for the top 20 Hybrid Feature Set models ranged from 0.71–0.73 and were higher than those of Set 1, Set 2, or Augmented Feature Set 1 on the independent data set (p = 5.8e-9, p<2.2e-16, and p = 3.7e-12 when compared with Set 1, Set 2, and Augmented Feature Set 1, respectively).

**Table 15:** Performance of the top models consisting of mixtures of the top Set 1 and Set 2 features on the independent data set (Hybrid Feature Set).

F1	Precision	Recall	Feature Combination	Kernel Degree
0.73	0.67	0.78	msf-i, diff-at-dens, msf-a, asaavg, asascavg, asabb1, asabbavg	2
0.73	0.67	0.78	msf-i, diff-hbond, msf-a, Ca-disp, asasc1, asabbavg	2
0.73	0.67	0.78	msf-i, diff-hbond, msf-a, Ca-disp, asasc1, asabb1	2
0.73	0.67	0.78	msf-i, diff-hbond, msf-a, Ca-disp, asa1, asabbavg	2
0.73	0.67	0.78	msf-i, diff-hbond, msf-a, asaavg, asasc1, asabb1	2
0.72	0.71	0.73	diff-hbond, msf-a, Ca-disp, asaavg, asasc2, asascavg, asabbavg	3
0.72	0.68	0.76	diff-hbond, msf-a, Ca-disp, asa1, asa2, asaavg, asasc1, asascavg, asabbavg	3
0.72	0.66	0.78	msf-i, diff-hbond, msf-a, Ca-disp, asa1, asabb1	2
0.71	0.64	0.81	msf-i, diff-at-dens, asaavg, asascavg, asabb1, asabbavg	3
0.71	0.64	0.81	msf-i, diff-hbond, msf-a, Ca-disp, asa1, asabb1, asabbavg	3
0.71	0.64	0.81	msf-i, diff-hbond, msf-a, asa1, asabbavg	3
0.71	0.64	0.81	msf-i, diff-hbond, msf-a, asa1, asasc1, asabbavg	3
0.71	0.64	0.81	msf-i, diff-hbond, diff-at-dens, msf-a, asa1, asascavg, asabb1, asabbavg	3
0.71	0.62	0.84	msf-i, diff-at-dens, msf-a, asa2, asaavg, asasc2, asascavg, asabb1	3
0.71	0.67	0.76	msf-i, diff-hbond, msf-a, Ca-disp, asasc1, asabb1, asabbavg	2
0.71	0.67	0.76	msf-i, diff-hbond, msf-a, Ca-disp, asaavg, asasc1, asabb1	2
0.71	0.67	0.76	msf-i, diff-hbond, msf-a, Ca-disp, asa1	2
0.71	0.67	0.76	msf-i, diff-hbond, msf-a, Ca-disp, asa1, asasc1, asabbavg	2
0.71	0.67	0.76	msf-i, diff-hbond, msf-a, Ca-disp, asa1, asasc1, asabb1, asabbavg	2
0.71	0.67	0.76	msf-i, diff-hbond, msf-a, asa1, asabbavg	2

Precision, recall, and F1 scores were calculated from the results on the independent data set. Listed are the top scoring feature/kernel degree combinations as ranked by F1 on the independent data set.

### Structural Analysis of Predicted Hotspots

To investigate the topology of predicted allosteric hotspots, we considered the predictions made by the top 9 most precise Hybrid Feature Set models for each residue of each protein in the independent data set (Table 16). That is, each residue was labeled in the protein structure by color according to the number of the top 9 highest-precision models that predicted a hotspot for that residue (Table S3). Naturally, one could simply consider the top-scoring model only, but we assert that such a voting scheme gives a more realistic picture of the pattern of hotspots, because it offsets the limitations of any single model alone. Moreover, adopting this voting scheme and labeling residues according to the number of votes they receive from the models for hotspot or non-hotspot reveals residues that are intermediate in terms of their hotspot/non-hotspot character, rather than yielding a simple binary prediction. We used the 9 most precise models for this analysis, because this ensures a minimum of false positive predictions and thus has the greatest likelihood to uncover the sparse network of interactions consisting of only the most definite hotspot predictions. The Hybrid Feature Set models were used, as this feature set performed the best overall compared with the other feature sets.

**Table 16:** The top 9 models with the highest precision on the independent data set that were used in the structural analysis.

F1 train	P train	R train	F1 ind	P ind	R ind	Feature Combination	Kernel Degree
0.65	0.54	0.84	0.70	0.75	0.65	msf-i, diff-hbond, msf-a, Ca-disp, asa2, asaavg, asasc1, asasc2, asascavg, asabbavg	3
0.65	0.55	0.80	0.70	0.74	0.68	msf-i, diff-at-dens, Ca-disp, asaavg, asabb1, asabbavg	2
0.64	0.57	0.73	0.69	0.73	0.65	msf-i, diff-hbond, bfac-a, Ca-disp, asa2, asasc1, asabb1, asabbavg	2
0.63	0.56	0.70	0.69	0.73	0.65	msf-i, diff-hbond, diff-at-dens, msf-a, bfac-a, diff-bfac, asa1, asa2, asaavg, asasc2, asabbavg	2
0.63	0.52	0.80	0.69	0.71	0.68	msf-i, diff-at-dens, Ca-disp, asa1, asaavg, asabbavg	2
0.65	0.55	0.80	0.69	0.71	0.68	msf-i, diff-at-dens, msf-a, asa1, asa2, asaavg, asasc2, asabbavg	3
0.64	0.57	0.73	0.69	0.71	0.68	def-energ-i, msf-i, diff-hbond, bfac-a, diff-bfac, asa1, asa2, asasc1, asascavg, asabbavg	2
0.64	0.56	0.75	0.69	0.71	0.68	def-energ-i, msf-i, diff-hbond, msf-a, Ca-disp, asa2, asasc1, asasc2, asascavg, asabb1	3
0.64	0.57	0.73	0.69	0.71	0.68	def-energ-i, msf-i, diff-hbond, diff-at-dens, bfac-a, diff-bfac, asa2, asaavg, asasc2, asabb1	2

The performance on both the training (abbreviated train) and independent (abbreviated ind) data sets are given. The F1, Precision (P) and Recall (R ) values for each model are reported based on their performance on the training and independent data sets.

In doing this analysis, we assessed whether predicted hotspots form a network pattern in the protein structure, in light of previous work showing the existence of networks of contiguous residues connecting effector and substrate sites in allosteric proteins [25], [30], [44], [50]–[52]. Qualitative inspection of the spatial distribution of predicted hotspot residues in the five proteins in the independent data set showed that predicted hotspots tended to be centrally located in the protein molecules and form a dense network pattern, while predicted non-hotspots tended to be solvent-exposed or on the periphery. This is consistent with the idea that residues that relay allosteric signals form a communication network within the molecule.

**Figure 4:**
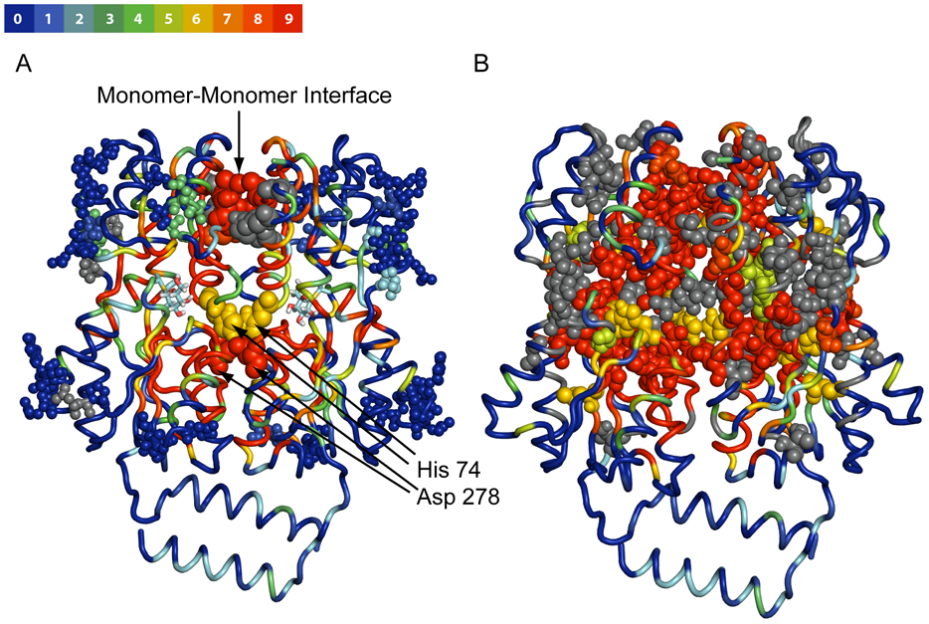
Hotspot predictions mapped to the inactive state structure of lac repressor. (A) Predictions made by the top 9 highest-precision Hybrid Feature Set models according to the voting scheme for lac repressor mapped onto the inactive state structure (1tlf). Experimentally tested residues rendered in van der Waals spheres, with known non-hotspots in small van der Waals spheres and known hotspots in larger ones. For other residues, the prediction is shown along the backbone trace, but no experimental data is available to test the prediction. Each residue in the structure is colored according to a blue→green→red heat map, where the extremes are as follows: red represents residues predicted to be hotspots by 9/9 of the models and blue residues to be predicted hotspots by 0/9 models (predicted non-hotspots by 9/9 models). (Refer to color bar above for exact mapping of the number of predicted hotspots to the color.) For ease of viewing only one set of dimers (chain A and B) is shown. His 74 and Asp 278, residues not in the independent data set but were studied experimentally and found to be allosterically active, are rendered in van der Waals mode as well [63]. Correct positive (hotspot) and negative (non-hotspot) predictions are colored according to the heat map, while false predictions are colored gray. The inducer molecule IPTG is rendered as sticks and colored by element. (B) Here the complete set of residues that caused the I^S^ phenotype are rendered in van der Waals spheres. The hotspots depicted in A. are a subset of these for which no substitution caused an I^−^ phenotype (completely nonfunctional). Incorrect predictions, i.e. false negatives, are colored in gray.

Furthermore, the locations of predicted hotspots and non-hotspots in the protein structure and the known functions of the structural elements of each protein system gave insight into the functional significance of the predictions. For *lac* repressor, a large number of predicted hotspots were found at the monomer-monomer interface (Figure 4a; Table S3), especially where the respective N-terminal domains of the two monomers interact. At this interface, significant alterations of residue-residue interactions occur in the allosteric transition [58]–[60]. Mutations in this region result in a non-inducible (i.e. allosterically unresponsive) phenotype [61],[62]. In addition to residues designated as hotspots and non-hotspots that were included in the independent data set, a key interaction at the monomer-monomer interface, a salt bridge between His 74 and Asp 278 that has been found to be important for the allosteric transition in this system [63], is highlighted in Figure 4a. Both of these residues were predicted hotspots by a majority of the models. A striking observation is the asymmetry of some of the predictions between the monomers. For instance, Lys 84, a known hotspot in the independent data set, is a predicted hotspot by 8 out of 9 models in chain A, but in chain B, it is a predicted hotspot by 4 out of 9 models (Table S3). This is consistent with the observed crystallographic asymmetry between the monomers, especially in the vicinity of Asp 149 [60],[64],[65]. Indeed, we found an all-atom RMSD of 5.55 Å between chain A and B in the inducer-bound state (PDB code 1TLF). Moreover, our finding is supported by the MD simulation study of Flynn et al. [64], who observe structural asymmetry between monomers during targeted MD simulations of the allosteric transition from the DNA-bound to the inducer-bound state and even during the equilibration phase, with an allosteric signal originating in the “trigger” monomer propagating to the “response” monomer.

**Figure 5:**
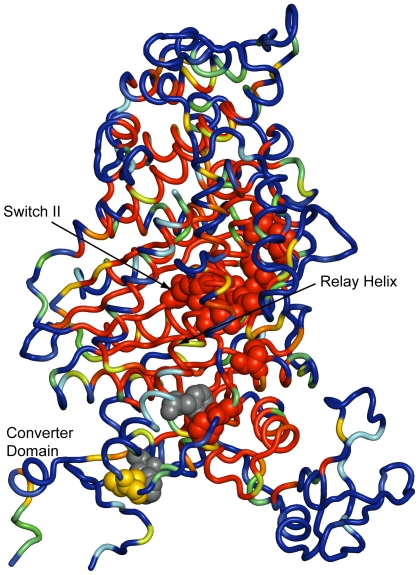
Hotspot predictions mapped to the inactive state structure of myosin II. Predictions made by the top 9 highest-precision Hybrid Feature Set models according to the voting scheme for myosin II motor domain mapped onto the inactive state structure (1vom). Refer to Figure 4 above for an explanation of the coloring. Residues that met our criteria for classification as hotspot and included in the independent data set are rendered in van der Waals spheres. Switch-II (a region with high homology to the switch region of G-proteins that couples GTP hydrolysis to effector-domain conformation) residues (454–459) are depicted in van der Waals spheres as well, and colored according to the heat map.

The top-precision Hybrid Feature Set models predicted many residues with known functional significance to be hotspots in the myosin II motor domain (Figure 5; Table S3). The models predicted hotspots in regions implicated in the coupling of ATP hydrolysis with movement along actin filaments, in particular, a large portion of the relay helix proximal to the ATP binding site and the entirety of Switch II. Specifically, the models identified in the relay helix Ile 499 as an intermediate hotspot, and Thr 474, Glu 476, and Phe 506 as strong hotspots, consistent with experimental data showing that mutations at these sites uncouple ATPase activity and motor function [66]–[68]. In addition, Cys 678 in the SH2 helix, which, along with the SH1 and relay helix, holds the converter domain in place, was identified as a hotspot. Mutations at this residue have been found to reduce the velocity of movement of myosin along actin [69]. The fact that the top-precision models predicted all of Switch II residues (454–459) to be hotspots is also noteworthy, for this region, which is close to the nucleotide-binding site, couples ATP hydrolysis with motor activity and is also homologous to the Switch II loop of G-proteins, which connects the GTPase site to the effector binding region, putatively playing a key role in coupling nucleotide hydrolysis to effector affinity and activity [70],[71].

Glucokinase is an enzyme that plays a role in regulating blood glucose levels through its function as a glucose sensor. Congenital mutations in this protein are associated with maturity-onset diabetes of the young [72]. The models identified a number of residues whose mutations cause this disease, which are the hotspots labeled in Figure S1 [73]–[76]. Interestingly, the models predicted residues that form contacts with the drug Compound A to be hotspots. This drug enhances the enzyme's affinity and enzymatic activity, and has been considered as a candidate for treatment of maturity-onset diabetes of the young [77]. This observation suggests that solvent-exposed predicted hotspots might be targets for drugs that have allosteric effects.

Glutamate dehydrogenase is an enzyme that plays an important role in nitrogen/carbon metabolism, oxidatively deaminating glutamate to 2-oxoglutarate, which is supplied to the TCA cycle [78],[79]. Like glucokinase, certain key mutations in this enzyme are of clinical relevance, as those that reduce sensitivity to the allosteric inhibitor GTP are associated with hyperinsulinism/hyperammonemia syndrome [80]–[83]. Mutations that result in this set of genetic diseases are localized to a region known as the antenna domain that contributes to allosteric regulation by mediating communication among the enzyme subunits [84],[85]. Like the other examples, a network- or mosaic-like pattern of predicted hotspots was revealed in the interior of the protein (Figure S2). However, the voting scheme performed only moderately well at predicting mutations implicated in hyperinsulinism/hyperammonemia syndrome, with two correctly predicted hotspots (Ser 444 and Gly 452) and one correctly predicted non-hotspot (Arg 466). This poorer performance may result from our model's tendency to predict hotspots in regions of the protein with low solvent accessibility.

Thrombin is a serine protease that plays key roles in both promoting and preventing clotting [86]–[91]. Although thrombin is regulated in many ways, one of its most well documented allosteric regulators is Na^+^ [87],[92],[93], whose binding shifts the conformation from the “slow” form with anticoagulant activity to a “fast” form with procoagulant activity [87], [94]–[100]. Of the residues that were observed by Pineda et al. to be critical to the allosteric transition, based on a three-fold change in the specificity ratio upon mutation to alanine [26], Asp 189, Ser 214, and Val 163 were identified in our analysis as strong hotspots, and Thr 172 was an intermediate hotspot (predicted by 6 out of 9 models; Table S3 and Figure S3). Additionally, Asp 221, another key hotspot according to Pineda et al. [26], was a predicted hotspot in 4 out of 9 models. Asp 189 plays an important structural role in thrombin allostery, coordinating waters to which the allosteric ligand, Na^+^, is bound [87],[101] and forming important interactions with the active site in the slow-to-fast transition. Thr 172 stabilizes the 220-loop of the Na^+^ binding site and links Glu 217 of the allosteric core to the active site [26],[87],[102]. Ser 214, as well, makes important links between the allosteric core and the active site [26],[103]. Strikingly, all residues that exhibited no change in their specificity ratios according to Pineda et al. [26] were predicted as strong non-hotspots (Table S3 and Figure S3). These residues are Asp 60E, Lys 60F, Asn 60G, His 71, Thr 74, Trp 96, Arg 97, Glu 97A, Arg 175, and Trp 245 (Table S3 and Figure S3).

In addition, Tyr 225 and Tyr 184A, two residues designated as part of the allosteric core [26],[103], were strong (predicted by 9 out of 9) and intermediate (predicted by 6 out of 9) hotspots, respectively. However, we did not include these residues in the independent data set, as their mutations to alanine did not meet the significance threshold (>three-fold) for the change in specificity ratio [26]. Tyr 225 is crucial for Na^+^ allostery, allowing Lys 224 to adopt a conformation able to coordinate Na^+^ and forming the water channel that connects the Na^+^ allosteric site to the substrate-binding site [26],[104]. Tyr 184A stabilizes one of the water molecules to which Na^+^ is coordinated.

Since *lac* repressor has been subjected to the most exhaustive site-directed mutagenesis of any of the protein systems in the independent data set [61], we considered *lac* repressor as a model system for comparing our predictions with the “true” distribution of hotspots in a protein. That is, *lac* repressor allows us to evaluate the sparsity of a network of experimentally determined hotspots and the degree of overlap between the true network and the predicted network. This is not possible with the other proteins in our independent data set, because they were mutated to one or two other residues at most in their respective experimental studies, giving an incomplete picture of the propensity of mutations to perturb allostery. Markiewicz et al., on the other hand, substituted every residue in *lac* repressor with 12–13 others. Additionally, only a small fraction of residues in the other proteins were studied by mutagenesis, while all the residues in *lac* repressor were studied. For our analysis, we considered all residues that caused the I^S^ phenotype (unresponsive to allosteric inducer). This is a larger set of hotspots than was included in the independent data set, because this larger set includes residues that result in a strong I^−^ phenotype (nonfunctional) after certain substitutions. Hotspots of the independent data set were a subset of the residues associated with I^S^ for which no substitution caused a strong I^−^ phenotype, as we wanted to focus the evaluation of our models specifically on residues relevant only to allostery and not to stability or folding.

First, we counted the number of residues associated with I^S^. 113 residues out of the 329 that were studied by Markiewicz et al. [61] had at least one substitution which resulted in I^S^. When we labeled the structure with all the I^S^ mutations, a dense pattern of mostly buried residues surrounding the inducer site emerged with appreciable aggreement with our predicted hotspots (Figure 4b; Table S4). Although this experimental finding seems contrary to the assertion that a sparse network of residues mediates allostery, we emphasize that this analysis considers *all* residues important in allostery and not just the most critical ones. Indeed, the most critical interactions, or those that most perturb the allosteric coupling free energy, may form a sparse network topology. The fact that our residues overlap appreciably with the complete set of residues observed experimentally to be important for allostery suggests that our models “cast a wide net,” identifying residues that are strongly important in allostery as well as those that have more modest effects when mutated. Additionally, we calculated the recall of the top–precision Hybrid Feature Set models on all I^S^ mutations. Given the asymmetry in the predictions owing to asymmetry in the dynamical properties and structure of *lac* repressor, we defined a true positive prediction as a residue predicted to be a hotspot by at least 5 of 9 models in at least one chain (Table S4). The recall was 0.83.

### Comparison of Machine-Learning Models with Statistical Coupling Analysis

We compared the performance of our best models with Statistical Coupling Analysis (SCA; [33]), a method used previously to investigate allosteric coupling [50]–[52], using code provided by the Ranganathan laboratory. The method relies on a multiple sequence alignment, followed by calculation of pairwise *ΔΔG* values and hierarchical clustering of the matrix *ΔΔG*. The multiple sequence alignment was done using the parameters prescribed by the developers of the method in other allostery studies [50]–[52]. All clustered matrices along with dendrograms are available in Supplementary Information. We identified clusters of residues based on simultaneous inspection of the clustered matrix of *ΔΔG* values and dendrograms (Figure S4). Clusters of allosterically important residues were defined by regions in the clustered *ΔΔG* matrix that contained significant amounts of points greater than or equal to 1.6 kT, which also coincided with cluster demarcations naturally defined by groups of branches in the dendrogram. For the training data set (consisting of 94 data points), SCA yielded a precision of 0.44, a recall of 0.16, and an F1 of 0.23. For the independent data set (consisting of 87 data points), SCA did considerably better, with a precision of 0.56, a recall of 0.51, and an F1 of 0.54. Considering all the data at once, SCA had a precision of 0.52, a recall of 0.32, and an F1 of 0.40.

## Discussion

In this work, we assembled a data set of residues that have been found experimentally to either perturb allostery (hotspots) or not (non-hotspots). We then calculated features for each data point, i.e., mutation site, to train machine-learning models that can predict a mutation's impact on allostery. We compared the performance of models based on structural, dynamic, network, and informatic features (Feature Set 1 and Augmented Feature Set 1) with ones trained on structural features requiring both inactive and active state structures (Feature Set 2). An advantage of our approach is that the models make automatic predictions about whether a residue is a hotspot or non-hotspot, avoiding the need for qualitative assessment or manual data analysis, and make use of a broad range of residue-level attributes implicated in allostery. Furthermore, our methods do not require long simulations or free energy calculations, which are difficult to perform when screening a large number of residues.

After testing all possible combinations of features on the training data set, we evaluated feature usage by the top-scoring models to provide insights into what may be residue-level signatures of allostery. In top-scoring models using Feature Set 1, deformation energy, mean-squared fluctuation, B-factor, atomic density, hydrogen bonding, and local structural entropy were predominant. In Feature Set 2, α-carbon displacement and solvent-accessible surface area measures predominated. We then combined the features that were predominant in top scoring models (based on the training data set) of each of these two feature sets and trained models using this feature set (Hybrid Feature Set). It was this hybrid set that performed best on the training and independent data sets.

### Examination of Feature Usage by Top-scoring Models

Features that predominate in high scoring models should be examined individually in the context of other work on allostery. Our examination of feature usage suggests that deformation energy is an important residue property in allostery. Deformation energy reflects a residue's participation in a protein hinge, and one can envision that hinge regions would coincide with residues of allosteric relevance. Others have applied residue-level constraints and analyzed their effects on the protein structure to define domains within a protein [105]. In a similar vein, Jacobs et al. [106] analyzed the network of constraints in a protein to define domains and predict flexible regions. Kovacs et al. [107] defined deformability in a stress tensor formulation using normal modes and applied this method to a set of kinases, two of which are known to be allosteric (cyclin-dependent kinase and cAMP-dependent protein kinase), demonstrating good agreement with experimentally determined hinge regions. The ability of constraint or deformation measures to define domains taken together with work demonstrating inter-domain communication as a basis for allostery [43], [46]–[48],[52] is consistent with the observed importance of deformation as a residue property in allostery in this study. We also considered the deformation energy of a residue in the active state along with the difference in this measure between the active and inactive state as features, and retrained combinations of the top 8 Feature Set 1 features supplemented with these two features. Top models trained using this feature set performed better on the training data set than the top models trained with all possible combinations of the original 18 features in Set 1, suggesting that deformation energy of the active state and changes in deformation energy are key features in describing allosteric properties of residues.

Measures related directly to solvent-accessible surface area (SASA) and those that correlate with SASA were also found to be features important for describing allostery. In Feature Set 1, the difference between normalized B-factors and atomic densities in the active and inactive states, along with the magnitudes of mean-squared fluctuations in the active and inactive states, were predominant features in the top models. Mean-square fluctuation and B-factor indirectly reflect the degree of exposure to the surface, while atomic density relates directly to solvent exposure. In addition, SASA-related features were found to be especially dominant in the top- scoring models created using Feature Set 2.

The observed prevalence of these features in top-scoring models was confirmed by inspecting the average values of these measures for hotspots and non-hotspots. Mean-squared fluctuation in the inactive state, atomic density in the inactive and active states, and most SASA measures were all significantly lower for hotspots than for non-hotspots, suggesting that hotspots tend to be buried (Table S5; Refer to table for p-values). Interestingly, the B-factor difference between the two states was larger for hotspots than non-hotspots. The difference was also moderately statistically significant (Table S5; p = 0.056), suggesting that hotspots tend to undergo greater changes in solvent-accessibility (and mobility) than non-hotspots. Taken together, these results point to the importance of residue burial or change in burial in allostery. Allosteric hotspots may derive their unique function from their tendency to be buried, allowing them to form internal networks within the structure, as well as change their solvent exposure, and, in turn, their microenvironment, during the allosteric transition.

Consistent with the importance of B-factor and mean-squared fluctuations in our models is the fact that residue fluctuations and correlations in fluctuations have been found computationally to yield putative allosteric networks of communication, with confirmation by experiment in some cases [36],[37]. In addition, other work demonstrated the importance of coupled changes in fluctuation to allostery [108]. In an indirect fashion, one can see a parallel between the importance of fluctuations and coupled fluctuations and work using the COREX algorithm [39], which revealed functionally relevant thermodynamic couplings based on the relative distributions of residue folding states [40]–[42]. The key parallel lies in the fact that this approach models the native-state as ensembles of microstates in which residues may exist in either a folded or unfolded state. These microstates can be considered analogous to thermal fluctuations or local pico- to nanosecond-scale motions that allow the protein to sample conformations separated by low-lying energy barriers.

The observed prevalence of another feature related to changes in solvent-accessible surface area in Feature Set 1, the difference in atomic density between active and inactive states, can also be related to important work in allostery. In particular, this finding is consistent with work showing the ability of networks of changes in residue contacts to identify putative allosteric communication and experimental hotspots [44].

A striking result from our analysis was the prevalence of local structural entropy, which is essentially a measure of the potential variability in protein secondary structure. The importance of the variability of secondary structure can be related to work using COREX [39], as this method relies on generating ensembles of structures where contiguous groups of residues are permitted to exist either in a folded or unfolded state, highlighting the utility of considering local structural variability. Hotspots on average had lower local structural entropy than non-hotspots (Table S5; p = 0.032), suggesting that hotspots have greater stability in their local secondary structure than non-hotspots.

Our analysis further revealed the importance of differences in hydrogen bonding between the inactive and active states, underscoring the role of this feature in governing processes that require microenvironmental specificity. We found that hotspots undergo greater changes in their hydrogen-bonding network in the allosteric transition than non-hotspots (Table S5; p = 0.031). This observation is consistent with the hypothesis that allosterically important protein regions undergo changes in their microenvironments in the allosteric transition. Protein-protein binding is one such process that requires a high degree of microenvironmental, or residue scale, specificity and depends highly on hydrogen bonding [109]–[112]. A similar dependence on microenvironmental specificity may underlie allosterically relevant domain-domain interactions [43], making it reasonable to hypothesize that changes in the domain-domain chemical microenvironment, including changes in hydrogen bonding, could be critical for allostery. Recently, Datta et al. [31] found a network of hydrogen bonds connecting the allosteric site to the active site within a monomer of caspase-1, suggesting that intradomain hydrogen bonding can mediate allosteric effects as well.

We were surprised to observe the low occurrence in the top models of the two network related properties, node degree and perturbation of the clustering coefficient upon node removal of the inactive state, given the demonstration that proteins are small-world networks [113],[114]. It may be the case that network properties of a single static structure are insufficient to describe allostery, which is defined by two-end state structures and potential intermediates. This is supported by the work of Daily et al. [44], in which important network relationships were inferred using both end-state structures.

### Models Requiring a Single Protein Structure

We examined our top scoring models from Feature Set 1 to determine if any of them required only a single structure, since in many systems, the crystal structure for only one conformation has been solved. Models that required the inactive state structure alone were found among the top 300 models, but none required only the active state structure. This suggests that the inactive state encodes a greater amount of relevant functional information than the active state. This is consistent with the observation that the inactive state is predisposed toward adopting functionally relevant conformations and can undergo the allosteric transition in the absence of effector [20]–[22].

### Combining Feature Set 1 and 2: Hybrid Feature Set

Because neither Feature Set 1 nor 2 appeared to be absolutely superior in performance, we created an optimal “hybrid” feature set by combining the top features of each. The hybrid set outperformed either set 1 or set 2 individually. Specifically, top Hybrid Feature Set models achieved the highest F1 scores on the training data set and independent data sets with a statistically significant (p<2.2e-16; for both data sets) improvement over the non-mixture feature set that scored best on the training data, Set 2. This result suggests that optimal predictions of allosteric functional properties from protein structure and sequence must account both for dynamic properties of the protein structure and for structural differences between the end-states. Moreover, one can say that empirical structural observations can work synergistically with dynamical properties that are based on a simple mechanical model, i.e., the elastic network model for normal mode calculations.

### Comparison of SVM models with Statistical Coupling Analysis (SCA)

Top-scoring SVM models trained using Feature Set 1, Set 2, Augmented Set 1, and the Hybrid Feature Set outperformed SCA in sensitivity and accuracy of class prediction. The difference in performance could be due to two reasons. First, SCA is based strictly on sequence, whereas our methods rely on sequence, structural, dynamical, and network features. Second, SCA was not originally developed as an allosteric hotspot-prediction method *per se*, but as a method for revealing thermodynamic coupling between residues more generally. Thermodynamic coupling underlies diverse protein properties, in particular folding, not just allostery. Indeed, observed patterns of evolutionary coupling in the WW domain family have been successfully used to design stably folding novel WW domain sequences [115]. While SCA lent early insights to residue-level thermodynamic coupling in key systems, we see that features based on protein structures provide the essential information for describing allostery. Nonetheless, the elegance and utility of the SCA method, which yields relevant information with a single measure, qualifies it as an important and complementary tool.

### Structural Analysis of Predicted Hotspot Residues Using Hybrid Feature Set Models

To shed light on the pattern of hotspots in the structures, we applied a voting scheme to the Hybrid Feature Set models with the highest precision on the independent data set to make predictions for every residue of each protein in the independent data set. This voting among models was adopted to avoid the limitations of any single model in predictive power. Furthermore, this scheme yields a continuum of predictions based on how many models predict a hotspot or non-hotspot for each residue. That is, this method not only predicts which residues are strong hotspots or non-hotspots, in which cases the models cast a unanimous or nearly unanimous vote for hotspot or non-hotspot, but it also uncovers residues with intermediate relevance to allostery, where there is not a large majority of models predicting either class.

Predicted hotspots tended to occur at highest densities in the interior of the structures, while non-hotspots tended to be found in the periphery of the proteins studied, consistent with the work of others demonstrating the importance of internal networks of residues connecting distant sites in allosteric proteins [25], [30], [44], [50]–[52]. Our predictions were found to be consistent in many cases with point mutant data and with experimentally elucidated functionalities of the various structural motifs in the proteins that we studied. While the analyses in this work suggest that perturbation of buried residues is more likely to disrupt allostery than is perturbation of exposed residues, we caution against taking this conclusion generally. For example, most of the known hotspots in glutamate dehydrogenase were close to the protein surface, and our top-precision models missed some of these residues. Similarly, in thrombin, the known hotspots that were more buried were classified as hotspots, while solvent exposed hotspots were not.

We examined the topology of all residues whose substitution with any residue has been found experimentally to cause I^S^ in *lac* repressor [61] and compared it with the topology of hotspots predicted by the top precision Hybrid Feature Set models. This system is noteworthy in that all residues were substituted with 12–13 others in the study of Markiewicz et al., thus enabling us to compare our predictions with a case in which hotspots were exhaustively examined through experimental study. We found that the pattern of residues that cause I^S^ is dense and shows significant overlap with the predicted hotspots, suggesting that our method can yield insight into the true distribution of hotspots in a protein.

One notable observation was that in one of the proteins, glucokinase, residues that made contacts with the synthetic allosteric activator, Compound A, were all predicted hotspots and some of these were known hotspots included in the independent data set. Compound A enhances the activity of glucokinase and has been considered as a therapy for diabetes, as glucokinase acts as a glucose sensor that plays a role in the regulation of serum glucose levels. This result suggests that predicted solvent-accessible hotspots might be candidates for binding sites of small-molecule effectors that can rescue the behavior of mutant proteins. Liu and Nussinov [116] suggest in their study of mutants that modulate the function of von Hippel-Lindau protein allosterically (that is, the effects of the mutations are manifested distally) that such mutations can be mimicked by drugs. Moreover, allosteric approaches to targeting of G-protein-coupled receptors are increasingly recognized to be highly selective and have low propensity for side effects [117].

### Conclusion

We have demonstrated that machine-learning models using dynamical, structural, informatic, and network features can discriminate between allosteric hotspots and non-hotspots with high sensitivity and accuracy, that the patterns of predictions form a network of residues within the structures, and that hotspots correlate with regions of known functional relevance. In our structural analysis, we exploited the exhaustive nature of an experimental mutagenesis study of *lac* repressor [61] to approximate the “true” topological distribution of allosteric hotspots in the protein and compared this with the distribution of predicted hotspots. We have shown that our hotspot predictions overlap appreciably with experimental hotspots. One key observation is noteworthy, which is that *the pattern of experimental hotspots is dense*. Although this seemingly conflicts with the sparse networks observed by others [50],[51],[118], one must keep in mind that computational studies that rely on a single property like evolutionary co-conservation may yield incomplete information [50],[51], and that many experimental studies focus on only a few sites of interest [118].

We hope our methods can help experimentalists identify residues that contribute to mechanisms of allostery in proteins of interest. Typically, residues thought to participate in the allosteric transition are those that undergo significant structural alterations between the inactive and active states or those that interact at subunit interfaces. Thus, site-directed mutagenesis studies probing the allosteric transition tend to target these residues. However, other residues may play key roles in the transition yet are not targeted, since they do not undergo obvious structural rearrangements. The observed importance of dynamics in addition to structure suggests that traditional structure-based approaches to selecting candidate residues for mutagenesis may not give a complete picture of allosterically relevant residues. Our methods overcome this shortcoming by including dynamical as well as structural features. Predictions made by our methods may be used to guide experimentalists in their choice of residues to target in mutagenesis studies, in particular, residues that would not be considered relevant to allostery based on structural methods alone.

An important test of our methods will be whether predicted hotspot residues correlate with those whose mutations result in significant perturbation of the allosteric coupling free energy between sites, *ΔΔΔG*, defined as follows:
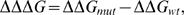
(11)where *ΔΔG_mut_* and *ΔΔG_wt_* are the site-site coupling free energies of the mutant and wildtype, respectively. Due to the paucity of such measures in the experimental literature, our training data was chosen based on indirect measures of coupling free energies. However, the most appropriate validation of our predictions would be the demonstration of a correlation between hotspot predictions and perturbation of coupling free energy averaged over all 19 possible mutations, 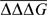.

The fact that one of the proteins we studied, glucokinase, exhibited extensive contacts of hotspot residues with a drug that shifts the protein to an active state suggests that hotspot residues could be candidates for drug targets. There exist enzymes in the drug discovery field for which finding active site inhibitors has been difficult [119], thus making allosteric sites an attractive alternative. Although the purpose of the current work was not allosteric site prediction *per se*, we posit that small molecules might be used to target residues that are putatively involved in allosteric communication, with the goal of modulating the allosteric transition. That is, binding of small molecules to hotspot residues might mimic the effect of mutations that shift the protein to either an inactive or active state, in cases where causing a shift in a protein's conformational distribution would be therapeutic. Conversely, binding of small molecules to hotspot residues may rescue the normal allosteric regulation in cases where an altered active-inactive distribution of a mutant protein is pathological; naturally, we realize this would be very challenging in practice. For multimeric proteins, subunit interfaces may be appropriate targets, as they often contain allosterically relevant residues and have sufficient solvent exposure to provide binding access for small molecules. Indeed, drugs targeting multimeric proteins have been shown to bind at subunit interfaces and exert their effects by stabilizing the inactive state [119]–[123].

An advantage of our techniques over other computational methods is that they are “meta-methods” that incorporate a variety of features. In contrast, many computational methods for inferring allosteric coupling derive their predictions from measurements of only single features. However, allostery is arguably a complex phenomenon that requires a more detailed model. Here, we have taken into account a number of features putatively relevant to allostery and combined them using a machine-learning algorithm to determine their relative importance in discriminating hotspots from non-hotspots. An advantage of these features is that most of them, with the exception of mean-squared fluctuation, deformation energy, and mutual information, can be calculated directly from the structures or sequences without the use of calculations that require heavily parameterized force fields or expensive simulations. Even the features that do rely on a parameterized model are calculated using the elastic network model, which has only two adjustable parameters. Thus, in creating a complex model for allosteric communication, we have striven to keep the individual features of the model as simple as possible.

## Materials and Methods

### Criteria for Classification as Hotspot or Non-hotspot

In the case of multimeric proteins, allosteric function is considered perturbed if, upon mutation: the Hill coefficient, a measure of cooperativity, is significantly altered; the protein is locked in either an inactive or active state (Hill coefficient of 1) even in the presence of effector; the concentration of allosteric inhibitor required to cause 50% inhibition is increased; binding or activity curves are altered from sigmoidal (characteristic of multimeric allosteric enzymes) to hyperbolic; or if inducibility is altered as measured by expression of a reporter gene *in vivo*, in the case of allosteric transcription factors whose response elements are designed to control expression of the reporter gene. In the latter case, care was taken to not include mutations that completely abolished inducibility, as this case cannot be distinguished from the case where the protein fails to fold or to be transcribed/translated *in vivo*.

Since a classification model must distinguish between positive and negative data, mutations that have no effect on allostery are included in the training data as controls. An additional criterion for inclusion in the training data set is that the mutation not be located in an effector or substrate-binding site. Naturally, it is possible for mutations that perturb binding to perturb the allosteric transition. In this study, the aim is to predict mutations that disrupt or alter the communication between effector and substrate sites (in the case of heterotropic cooperativity) or between substrate sites (in the case of homotropic cooperativity). Our training data set is a subset of those allosteric proteins compiled by Daily and Gray [1] for which sidechain substitution data is available. The training data consist of 44 hotspots and 50 non-hotspots from a set of allosteric enzymes, transcription factors, and signal transduction proteins (Table S1). The independent data set consists of 37 hotspots and 50 non-hotspots from a set of three allosteric enzymes, a transcription factor (*lac* repressor), and myosin II (Table S2).

### Calculation of Features

Eighteen attributes are computed for each protein in the training and independent data sets (Feature Set 1). Dynamical attributes are calculated with the program DIAGRTB, which calculates all-atom elastic network model (ENM) normal modes with rotational-translation blocking [124]–[127]. Here we used one residue per block. This method was used due to the fact that the large size of most proteins in the data sets necessitated a computationally cheap, yet accurate, method. The normal mode-based dynamical attributes are as follows:

#### Mean squared fluctuation

Atomic mean square fluctuations were calculated for both inactive and active conformers using the following formula:
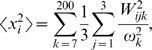
(12)where 〈*x^2^_i_*〉 is the mean square fluctuation of the *i*th atom, *W_ik_* is the *j*th component of the *i*th atom in the *k*th normal mode, and *ω_k_* is the frequency of the *k*th normal mode. The summation occurs over the nontrivial normal modes up to mode 200, since these correspond to the largest amplitude fluctuations that are most accurately calculated by ENM.

Since the actual numerical values of the mean square fluctuations are only meaningful within a protein and not across proteins, a method to determine the relative degree of fluctuation was required. To this end, the atoms were ranked according to the magnitudes of their fluctuation. The decile rank was determined for each atom of each of the mutant residues in the dataset, and a score for each residue was taken to be the average of the decile ranks for each atom in the residue. The difference in scores between mean squared fluctuation in the inactive and active states as well as the individual values were ascertained.

#### Deformation energy

Deformation energy was calculated for the inactive and active conformers as follows [128]–[130]:
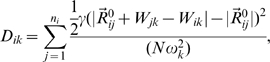
(13)where *D_ik_* is the deformation of the *i*th atom due to the *k*th normal mode, *γ* is the spring constant (set equal to1), and *R_ij_^0^* is the distance between atom *i* and atom *j* in the structure, *N* is the total number of atoms, and all other terms are as previously described. Scoring for each mutation site was performed as for mean square fluctuation. (Deformation energy score of the active state as well as the difference in scores between the inactive and active states were not part of Feature Set 1, but were included in the training of models consisting of the top eight highest performing Set 1 features supplemented with these two features.)

#### Mutual Entropy

Mutual entropy, or mutual information, between two coordinates *x_i_* and *x_j_* is defined as:
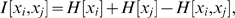
(14)where *H*[*x_i_*] is the entropy given the marginal distribution *p*(*x_i_*) and *H*[*x_i,_x_j_*] is the entropy given the marginal distribution *p(x_i_,x_j_)*. Here, the coordinates are inactive state alpha carbon atoms. The entropy *H[x_i_*] is as follows:
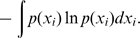
(15)


In this work, an approximation for estimating *H*[*x_i_*] was used, taken to be [131]:
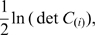
(16)where *C_i_* is the marginal covariance for the ith atom, and *C_ij_* is the same for the ith and jth atoms. Mutual information is thus estimated as:
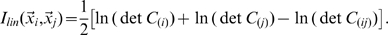
(17)


For each residue, a mutual information score was taken as the number of instances a given residue (represented by its alpha carbon) had an off-diagonal *I_lin_* greater than a threshold of 5.0, normalized by (i.e., divided by) the number of alpha carbons in the protein structure.

In addition to dynamic information based on normal modes, the following static-structure attributes were calculated:

#### B-factor

Mutation sites were ranked according to their B-factors in the same manner as applied in the case of mean-square fluctuation and deformation energy, that is, a decile rank score was used to normalize for variability in global protein flexibility. This was performed using both active- and inactive-state structures. The difference in scores between B-factor in active and inactive-states as well as the individual values were ascertained.

#### Atomic density

An average atomic density was determined for each residue in both the active and inactive states using FADE (Fast Atomic Density Evaluator; [132]), as well as the absolute difference in density between active and inactive states. The algorithm uses the fast Fourier transform to rapidly calculate atomic density. Here, the density at the grid point nearest each atom is determined, followed by averaging over the density of each atom in the residue.

#### Hydrogen bonding

Potential hydrogen bonds for residues in both active- and inactive-state structures were determined using the What-if program [133]. The absolute difference in the number of hydrogen bonds between bound and unbound structures was determined.

A number of network-based features were calculated for the inactive-state structure:

#### Node degree

Node degree was taken to be the total number of residues that contain at least one heavy atom within 5.0 Å of the residue (node) of interest.

#### Perturbation of the clustering coefficient

The clustering coefficient is defined as follows [114]. If residue *k* has N*_k_* residues in contact with it, the maximal number of possible contacts between the N*_k_* neighbors is N*_k_* (N*_k_*-1)/2. The clustering coefficient for the entire protein is
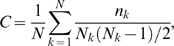
(18)where *n_k_* is the actual number of contacts that exist between the N*_k_* neighbors of residue *k* and N is the total number of residues. The perturbation in the clustering coefficient is the change in this value after a given residue of interest has been removed from the protein network.

Finally, a number of informatics features were calculated:

#### Local structural entropy

Local structural entropy is a measure of the propensity for variability in secondary structure within a given 4-residue site [134]. It is based on the probability of occurrence of a given secondary structure type at a 4-residue primary sequence site, which is used to compute a local structural entropy score. Eight secondary structure types are recognized by the algorithm: β-bridges, extended β-sheets, 3_10_-helices, α-helices, π-helices, bends, turns, and others. A local structural entropy score is obtained for a residue as the average over the four 4-mer windows containing the given residue.

#### Evolutionary conservation

The Consurf web server was used to determine a residue's conservation score, as determined by multiple sequence alignment [135].

#### Change in average structure

Calculations of features related to the change in average structure between active- and inactive-state conformations (Feature Set 2) were originally performed by Daily and Gray [1]. They are differences in various structural metrics between the active and inactive-state structures, and are as follows α-carbon displacement, side-chain root mean squared distance relative to the backbone atoms, angle between the α-β carbon bond in the inactive and active states, difference in the φ- and ψ-angles as well as the maximum of the two, difference in χ1 and χ2 side-chain torsion angles as well as the maximum, difference in the fractional change in a residue's contact environment as well as the maximum, secondary-structure type in inactive and active states, percent all-atom solvent-accessible surface area (SASA; relative to a model peptide) in inactive and active states and the average of the two, percent side chain SASA in inactive and active states and the average, and percent backbone SASA in inactive and active states and the average. Daily and Gray [1] used the program NACCESS [136] to calculate solvent-accessible surface area. These data were downloaded from Dr. Gray's laboratory website at http://graylab.jhu.edu/allostery/.

### Machine-Learning Algorithm

Support-vector machine learning was implemented using the Weka machine-learning package [57]. Second- and third-degree polynomial kernels were used. All possible combinations of the 18 features were input into the algorithm using either of the two kernel functions. A nine-fold, leave-one-out cross-validation of the data was used to learn a support-vector model for each fold, where the training of the model is performed using 8 of the 9 folds of the data, and the model tested on the remaining one. The performance of each feature/kernel combination was evaluated using metrics described under “Evaluation of Learned Models.” This cross-validation is performed to avoid a biasing of the SVM parameters due to overtraining. The same method was applied to the features calculated by Dr. Jeffrey Gray's laboratory. However, due to the size of the latter feature set (21 in all), training using all possible combinations taking 8–14 at a time could not be accomplished, as the number of such combinations requires an astronomical amount of computing time. Subsequent rounds of training using optimized combinations of features were performed using all possible combinations of these features and either of the two kernel functions.

The feature/kernel degree combinations that performed best in the training set were tested on the independent data set. Here, a single model was trained on the entire training data set using each of these highest performing feature/kernel degree combinations, and this model was subsequently tested on the independent data set.

### Statistical Coupling Analysis

We used position-specific iteration BLAST [137] with an E-value cutoff of 0.001 as previously prescribed by the developers of the SCA method in other allostery studies [50],[51] in assembling the sequences to be used for multiple-sequence alignment with ClustalW [138]. SCA and subsequent hierarchical clustering were performed using codes associated with methods outlined in previous work [33], [50]–[52].

For the case of myosin II, we used the results of the SCA analysis published by Yu et al. ([139]; Table 3).

### Evaluation of Learned Models

Precision, recall and F1 were calculated for each feature set and polynomial kernel combination used in the support vector machine learning, using a nine-fold cross validation for each combination. These same measures were calculated when evaluating the performance of models on the independent data set and when evaluating SCA on the training or independent data sets. *Precision* is the fraction of predicted hot spots that are true hot spots:
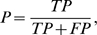
(19)where *P* is the precision, *TP* is the number of predicted true positives, and *FP* is the number of predicted false positives. Therefore, precision is essentially a measure of specificity. *Recall* is the fraction of true hot spots that are predicted hot spots:
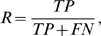
(20)where *R* is the recall and *F*N is the number of predicted false negatives. The denominator of this equation is equal to the number of actual positives. It is clear from this that recall is a measure of sensitivity of a method at detecting hotspots. The *F1* score measures the balance between precision and recall, and it is defined as follows:
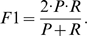
(21)


The feature/kernel degree combinations were ranked according to F1. For the calculation of these measures in evaluating the results of the cross-validated training, we pooled the *TP*, *FP*, *T*N, and *F*N of each of the nine models generated by the nine-fold cross-validation to calculate a *P*, *R*, and F1 for each feature/kernel degree combination tested in the training process.

To measure the statistical significance of differences between the performance measures of sets of models, a one-tailed, unpaired Student's T test was used.

## Supporting Information

Figure S1 Predictions made by the top 9 highest-precision Hybrid Feature Set models according to the voting scheme for glucokinase mapped onto the active state structure (1v4s). Each residue in the structure is colored according to a blue→green→red heat map, where the extremes are as follows: red represents residues predicted to be hotspots by 9/9 of the models and blue residues to be predicted hotspots by 0/9 models (predicted non-hotspots by 9/9 models). Experimentally determined hotspots and non-hotspots included in the independent set are rendered in van der Waals spheres (non-hotspots in small van der Waals spheres). For other residues, the prediction is shown along the backbone trace, but no experimental data is available to test the prediction. Correct true positive (hotspot) and true negative (non-hotspot) predictions are colored according to the heat map, while false negatives and false positives are colored gray. Glucose, the effector and substrate for this enzyme, is rendered in sticks and colored by element. Some correctly predicted true hotspots depicted in spheres in the figure (Met 210, Tyr 214, Val 452, and Val 455), along with two predicted hotspots not in the independent data set (Arg 63 and Tyr 215) also contact the allosteric drug Compound A (rendered in sticks and colored by element), which enhances the activity of the enzyme.
(0.21 MB PDF)

Figure S2 Predictions made by the top 9 highest-precision Hybrid Feature Set models according to the voting scheme for glutamate dehydrogenase mapped onto the inactive state structure (1nr7). Each residue in the structure is colored according to a blue→green→red heat map, where the extremes are as follows: red represents residues predicted to be hotspots by 9/9 of the models and blue residues to be predicted hotspots by 0/9 models (predicted non-hotspots by 9/9 models). Experimentally determined hotspots and non-hotspots included in the independent set are rendered in van der Waals spheres (non-hotspots in small van der Waals spheres). For other residues, the prediction is shown along the backbone trace, but no experimental data is available to test the prediction. Correct true positive (hotspot) and true negative (non-hotspot) predictions are colored according to the heat map, while false negatives and false positives are colored gray.
(7.20 MB PDF)

Figure S3 Predictions made by the top 9 highest-precision Hybrid Feature Set models according to the voting scheme for thrombin mapped onto the structure of the slow form (1sgi). Each residue in the structure is colored according to a blue→green→red heat map, where the extremes are as follows: red represents residues predicted to be hotspots by 9/9 of the models and blue residues to be predicted hotspots by 0/9 models (predicted non-hotspots by 9/9 models). Experimentally determined hotspots and non-hotspots included in the independent set are rendered in van der Waals spheres (non-hotspots in small van der Waals spheres), along with two additional residues that are part of the allosteric core, Tyr 225 and Tyr184A, but did not meet the criteria for inclusion in the independent data set. For other residues, the prediction is shown along the backbone trace, but no experimental data is available to test the prediction. Correct true positive (hotspot) and true negative (non-hotspot) predictions are colored according to the heat map, while false negatives and false positives are colored gray.
(5.63 MB PDF)

Figure S4 SCA data. Results for SCA are present for each protein from the training and independent data sets, except for myosin II where we relied on the previously published analysis by Yu et al. [F1]. a. Hierarchically clustered matrix of ΔΔG values and dendrogram where terminal branches correspond to residue indices of the protein sequence. Branches of the dendrogram corresponding to regions in the matrix containing clusters of high ΔΔG (regions with high fraction of points greater than or equal to 1.6 kT) are highlighted. The color scale is once displayed for CheY and applies to the subsequent protein systems. b. Magnification of the ends of the highlighted branches to display the residue indices, which are based on the numbering in the corresponding PDB file (except for thrombin, where negative numbers are for residues cleaved from prothrombin chain B and thrombin residues start at 1).
(1.78 MB PDF)

Table S1 Training data set. Given are the protein name, the PDB ID of the inactive state, the PDB ID of the active state, the residue that was mutated, the reference(s) where the effect(s) of the mutation is (are) described, and, in the final column, details of the experiment(s) in which the mutation was characterized. In the final column, first the point mutation(s) is (are) given, and this is followed by a brief synopsis of the experimental results. Abbreviations used: wt = wild type; coef. = coefficient; repr. = repression.
(0.11 MB RTF)

Table S2 Independent data set. Given are the protein name, the PDB ID of the inactive state, the PDB ID of the active state, the residue that was mutated, the reference(s) where the effect(s) of the mutation is (are) described, and, in the final column, details of the experiment(s) in which the mutation was characterized. In the final column, first the point mutation(s) is (are) given, and this is followed by a brief synopsis of the experimental results, except for lac repressor where at least 12 amino acid substitutions were made for each residue (The reader may refer to Markiewicz et al. [T44] and Suckow et al. [T45] for details.). Abbreviations used: wt = wild type; coef. = coefficient; repr. = repression; Is = not responsive to inducer (allolactose or isopropyl–D-thiogalactoside); I- = abolished DNA binding or misfolded.
(0.09 MB RTF)

Table S3 Classification of residues in the independent data set according to the voting scheme of the top 9 highest-precision Hybrid Feature Set models that was used in *Structural Analysis of Predicted Hotspots*. The numbers in the columns to the right of the true classification are the number of models out of the nine that predicted a hotspot for each residue. *hotspot = residues that perturb allostery for certain mutations, but did not meet our criteria for inclusion as hotspots in the independent data set. NA = residues not included in the independent data set but have structural properties relevant to allostery.
(0.16 MB RTF)

Table S4 Classification of residues whose mutation caused the I^S^ phenotype in at least one residue substitution. The voting scheme of the top 9 highest-precision Hybrid Feature Set models that was used in Structural Analysis of Predicted Hotspots was used for this classification. The numbers in the columns to the right of the residue index are the number of models out of the nine that predicted a hotspot for each residue.
(0.07 MB RTF)

Table S5 Average values of features of interest for hotspots and non-hotspots, along with the p-value (unpaired Student's T-test) signifying the statistical significance of the difference in the average value of each feature between hotspots and non-hotspots. Values with a strongly statistically significant difference (p<0.05) between the two classes are indicated by ** and in bold, and those with a moderate statistical significance are indicated by * and in bold italic. For Feature Set 1, dotted lines separate features that are based on dynamic structural features, local contact geometry, network-based features and conservation.
(0.02 MB RTF)
