# Supplementary material for: Structure-Based Predictive Models for Allosteric Hot Spots
Source: PLoS Comput Biol. 2009 Oct 9;5(10):e1000531. doi: 10.1371/journal.pcbi.1000531 (PMC2748687; doi:10.1371/journal.pcbi.1000531)
Supplement: Figure S1 — Predictions made by the top 9 highest-precision Hybrid Feature Set models according to the voting scheme for glucokinase mapped onto the active state structure (1v4s). Each residue in the structure is colored according to a blue→green→red heat map, where the extremes are as follows: red represents residues predicted to be hotspots by 9/9 of the models and blue residues to be predicted hotspots by 0/9 models (predicted non-hotspots by 9/9 models). Experimentally determined hotspots and non-hotspots included in the independent set are rendered in van der Waals spheres (non-hotspots in small van der Waals spheres). For other residues, the prediction is shown along the backbone trace, but no experimental data is available to test the prediction. Correct true positive (hotspot) and true negative (non-hotspot) predictions are colored according to the heat map, while false negatives and false positives are colored gray. Glucose, the effector and substrate for this enzyme, is rendered in sticks and colored by element. Some correctly predicted true hotspots depicted in spheres in the figure (Met 210, Tyr 214, Val 452, and Val 455), along with two predicted hotspots not in the independent data set (Arg 63 and Tyr 215) also contact the allosteric drug Compound A (rendered in sticks and colored by element), which enhances the activity of the enzyme. (0.21 MB PDF) [file pcbi.1000531.s001.pdf]

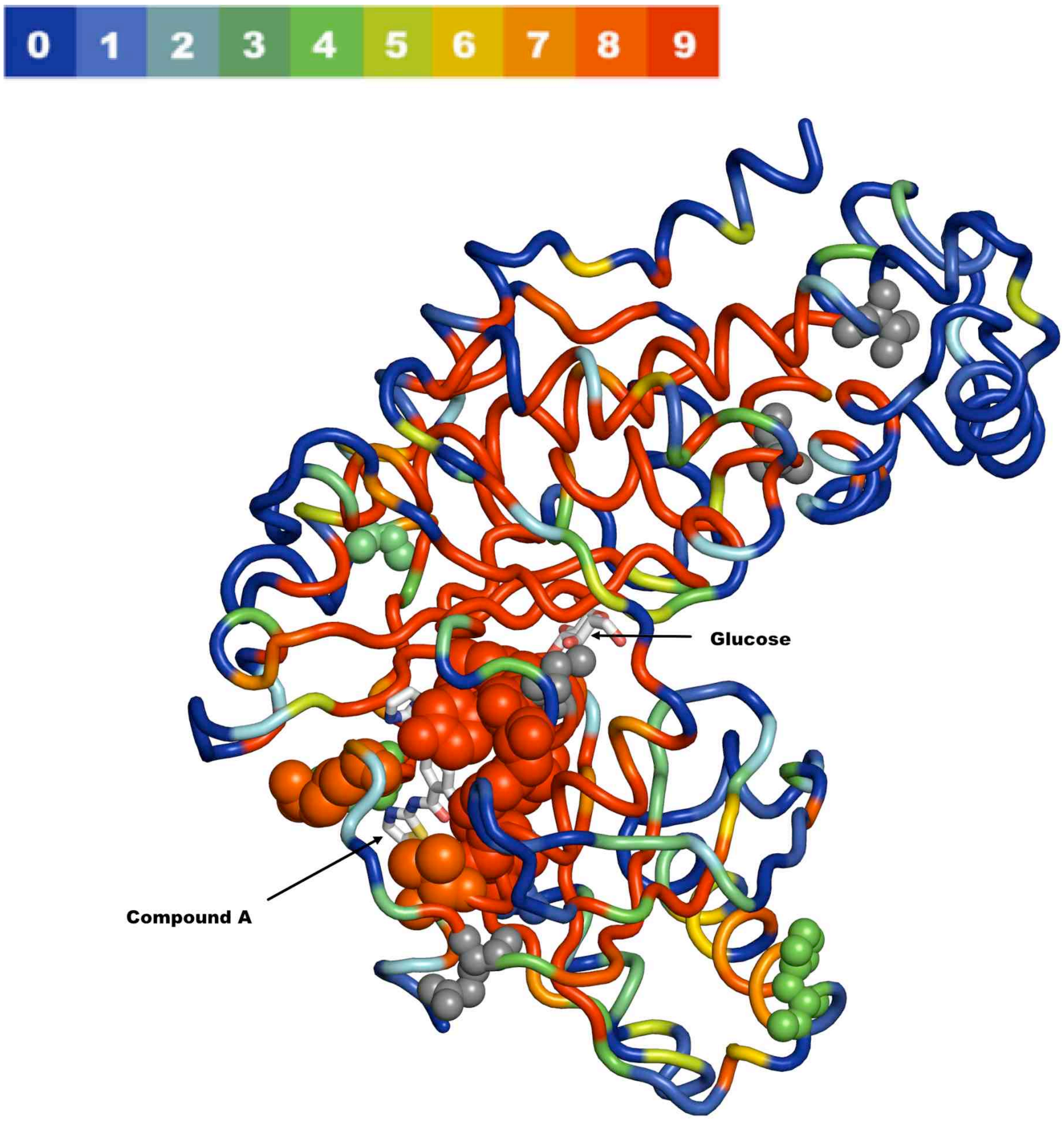


**Figure S1.** Predictions made by the top 9 highest-precision Hybrid Feature Set models according to the voting scheme for glucokinase mapped onto the active state structure (1v4s). Each residue in the structure is colored according to a blue->green->red heat map, where the extremes are as follows: red represents residues predicted to be hotspots by 9/9 of the models and blue residues to be predicted hotspots by 0/9 models (predicted non-hotspots by 9/9 models). Experimentally determined hotspots and non-hotspots included in the independent set are rendered in van der Waals spheres (non-hotspots in small van der Waals spheres). For other residues, the prediction is shown along the backbone trace, but no experimental data is available to test the prediction. Correct true positive (hotspot) and true negative (non-hotspot) predictions are colored according to the heat map, while false negatives and false positives are colored gray. Glucose, the effector and substrate for this enzyme, is rendered in sticks and colored by element. Some correctly predicted true hotspots depicted in spheres in the figure (Met 210, Tyr 214, Val 452, and Val 455), along with two predicted hotspots not in the independent data set (Arg 63 and Tyr 215) also contact the allosteric drug Compound A (rendered in sticks and colored by element), which enhances the activity of the enzyme.
