# Supplementary material for: Structure-Based Predictive Models for Allosteric Hot Spots
Source: PLoS Comput Biol. 2009 Oct 9;5(10):e1000531. doi: 10.1371/journal.pcbi.1000531 (PMC2748687; doi:10.1371/journal.pcbi.1000531)
Supplement: Figure S4 — SCA data. Results for SCA are present for each protein from the training and independent data sets, except for myosin II where we relied on the previously published analysis by Yu et al. [F1]. a. Hierarchically clustered matrix of ΔΔG values and dendrogram where terminal branches correspond to residue indices of the protein sequence. Branches of the dendrogram corresponding to regions in the matrix containing clusters of high ΔΔG (regions with high fraction of points greater than or equal to 1.6 kT) are highlighted. The color scale is once displayed for CheY and applies to the subsequent protein systems. b. Magnification of the ends of the highlighted branches to display the residue indices, which are based on the numbering in the corresponding PDB file (except for thrombin, where negative numbers are for residues cleaved from prothrombin chain B and thrombin residues start at 1). (1.78 MB PDF) [file pcbi.1000531.s004.pdf]

CheY a.

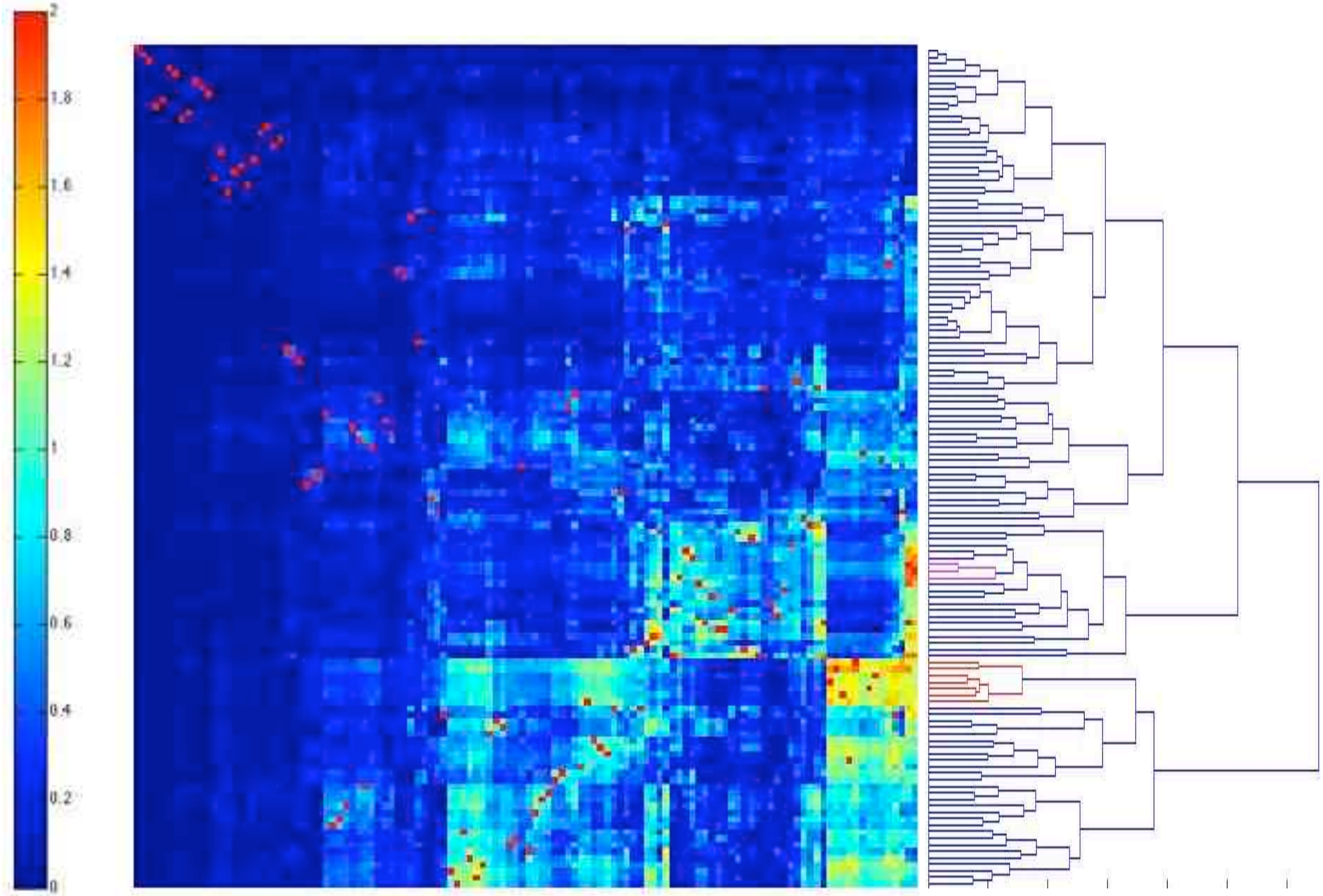

CheY b.

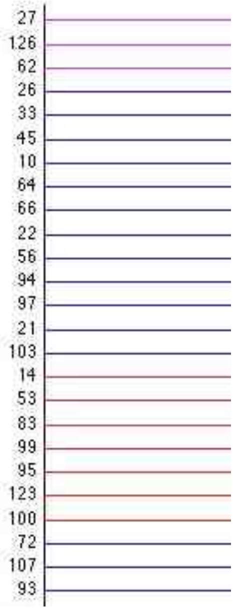
27
126
62
26
33
45
10
64
66
22
56
94
97
21
103
14
53
83
99
95
123
100
72
107
93

Purine repressor a.

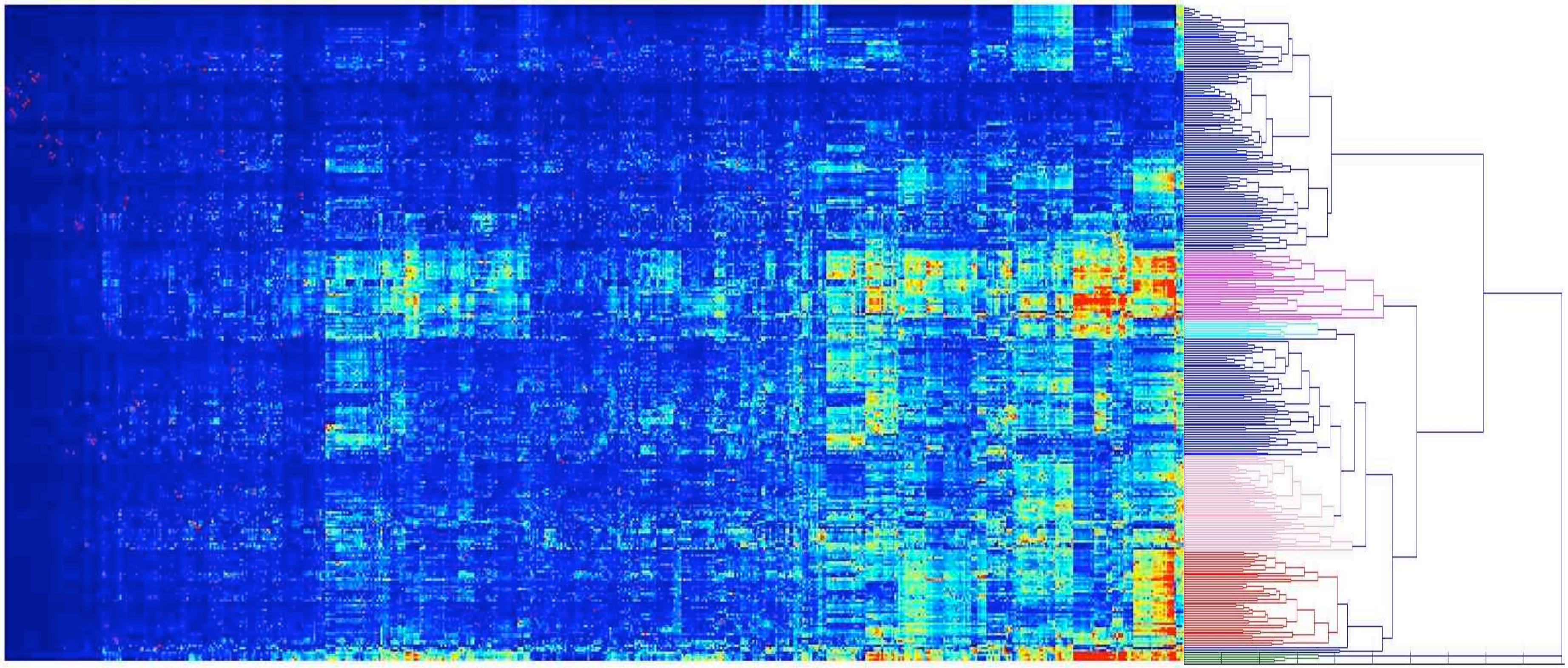

Purine repressor b.

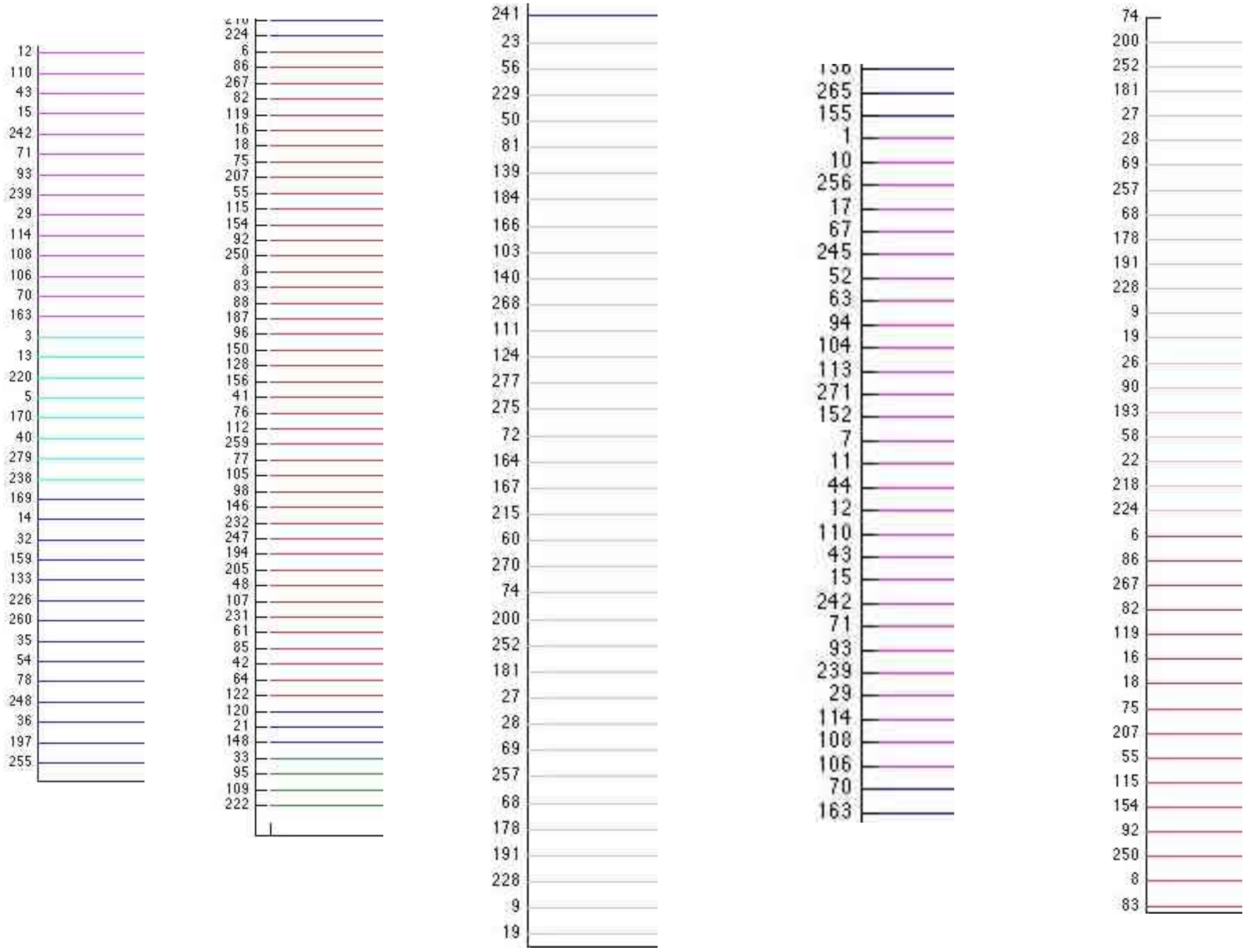

Tet repressor a.

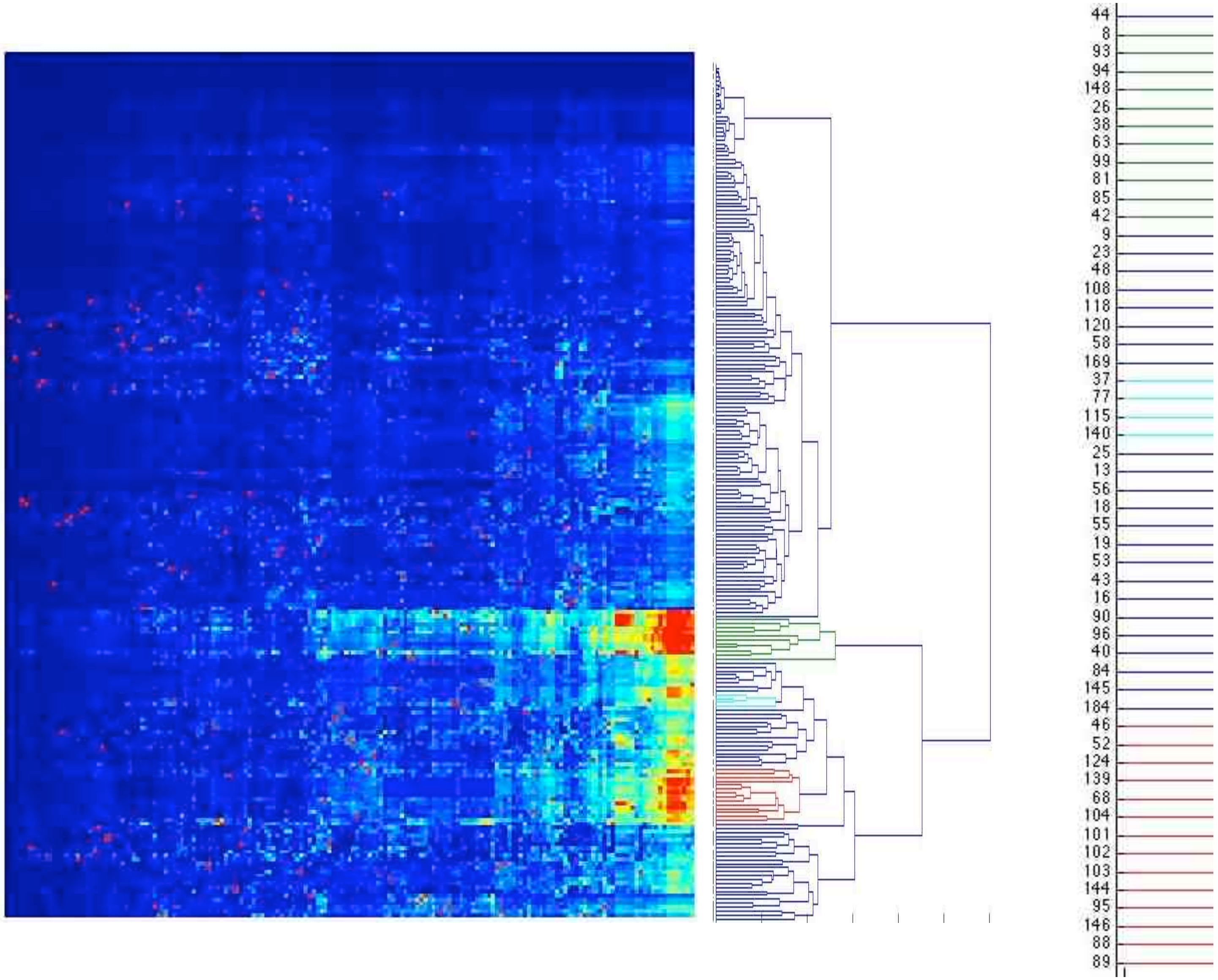

Tet repressor b.

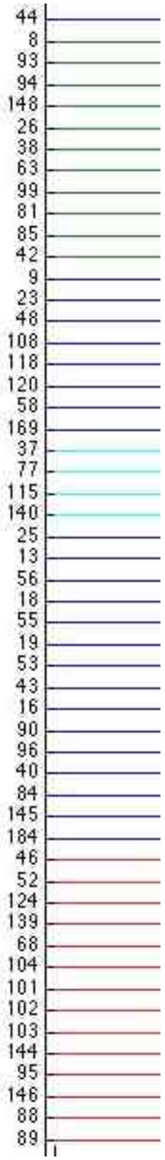

Hemoglobin, alpha subunit

a.

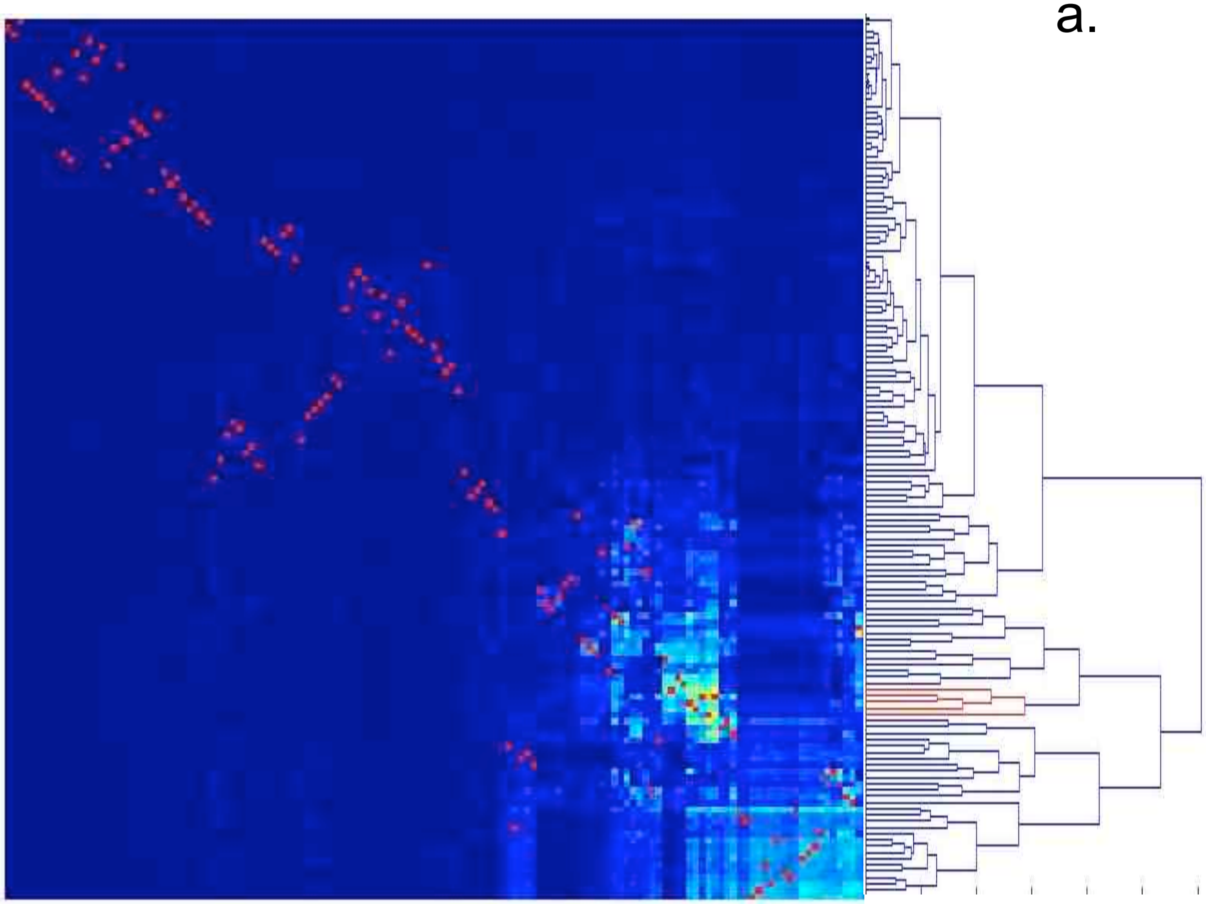

b.

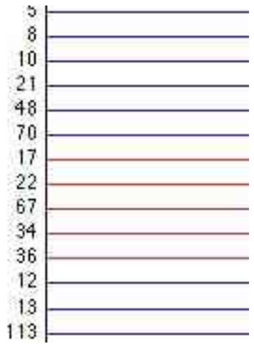

Hemoblobin, beta subunit

a.

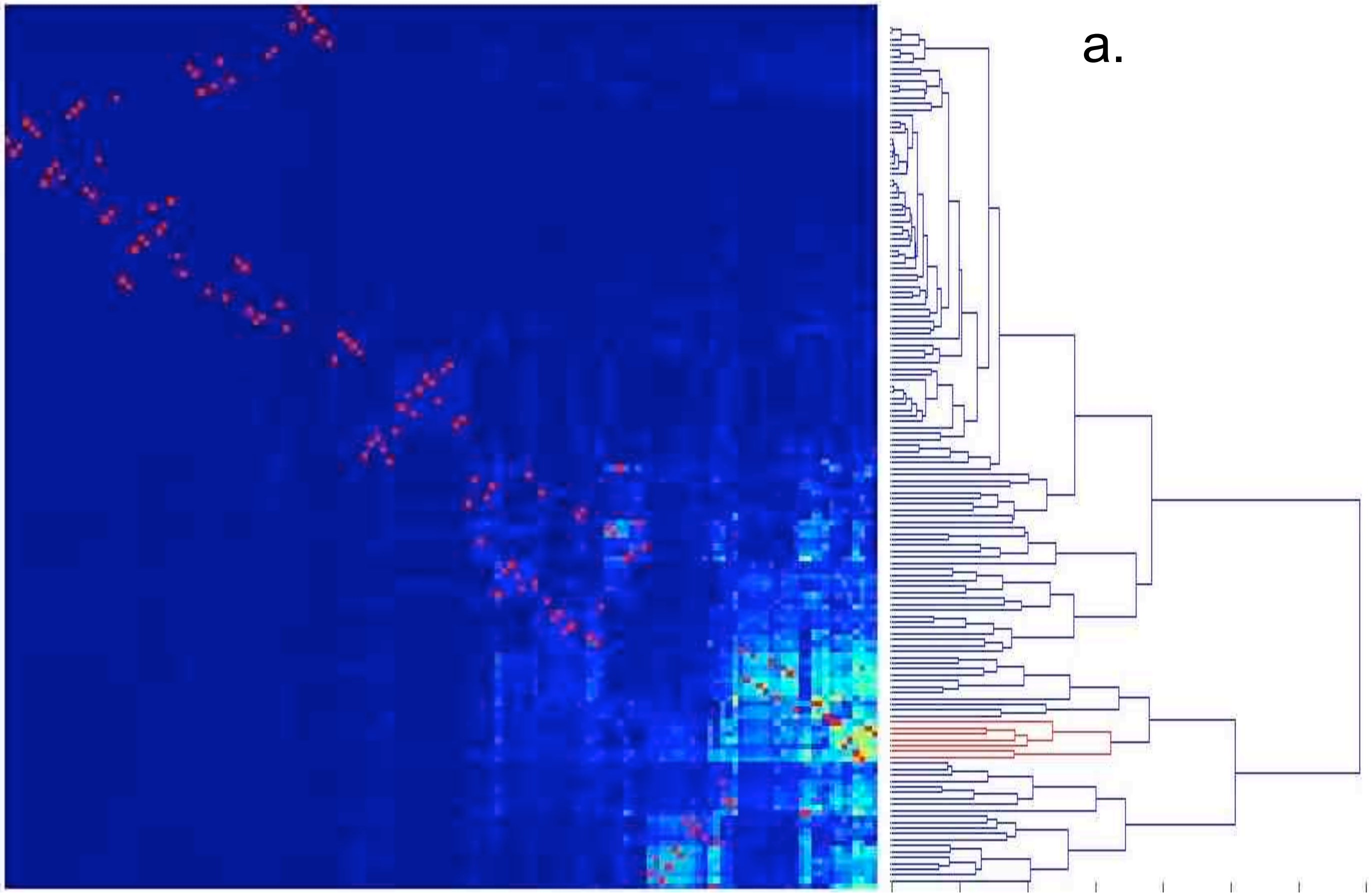

b.

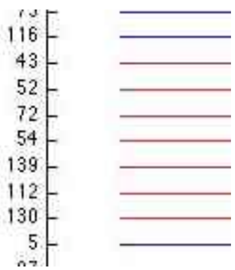

d-3-phosphoglycerate dehydrogenase a.

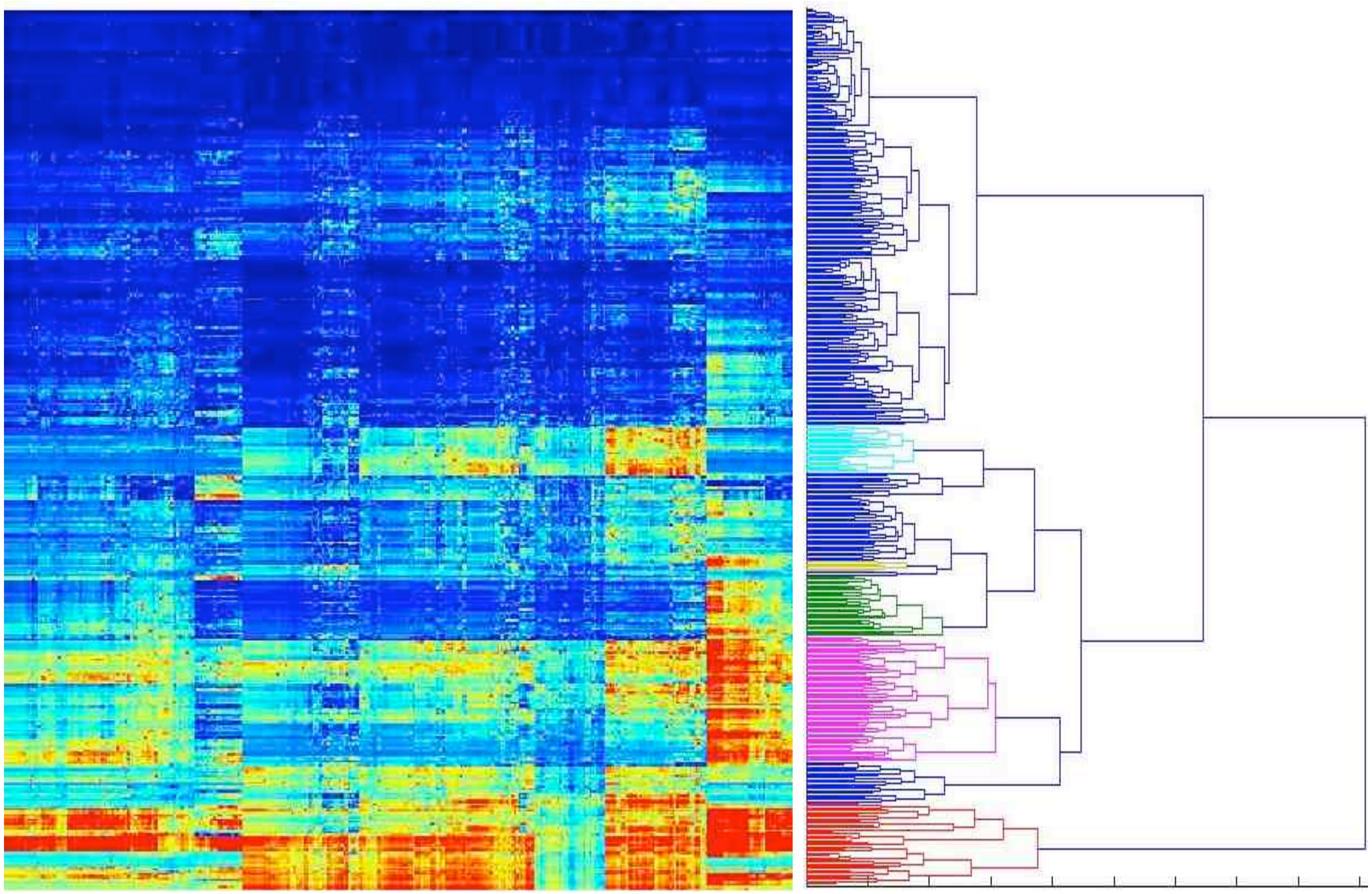

d-3-phosphoglycerate dehydrogenase b.

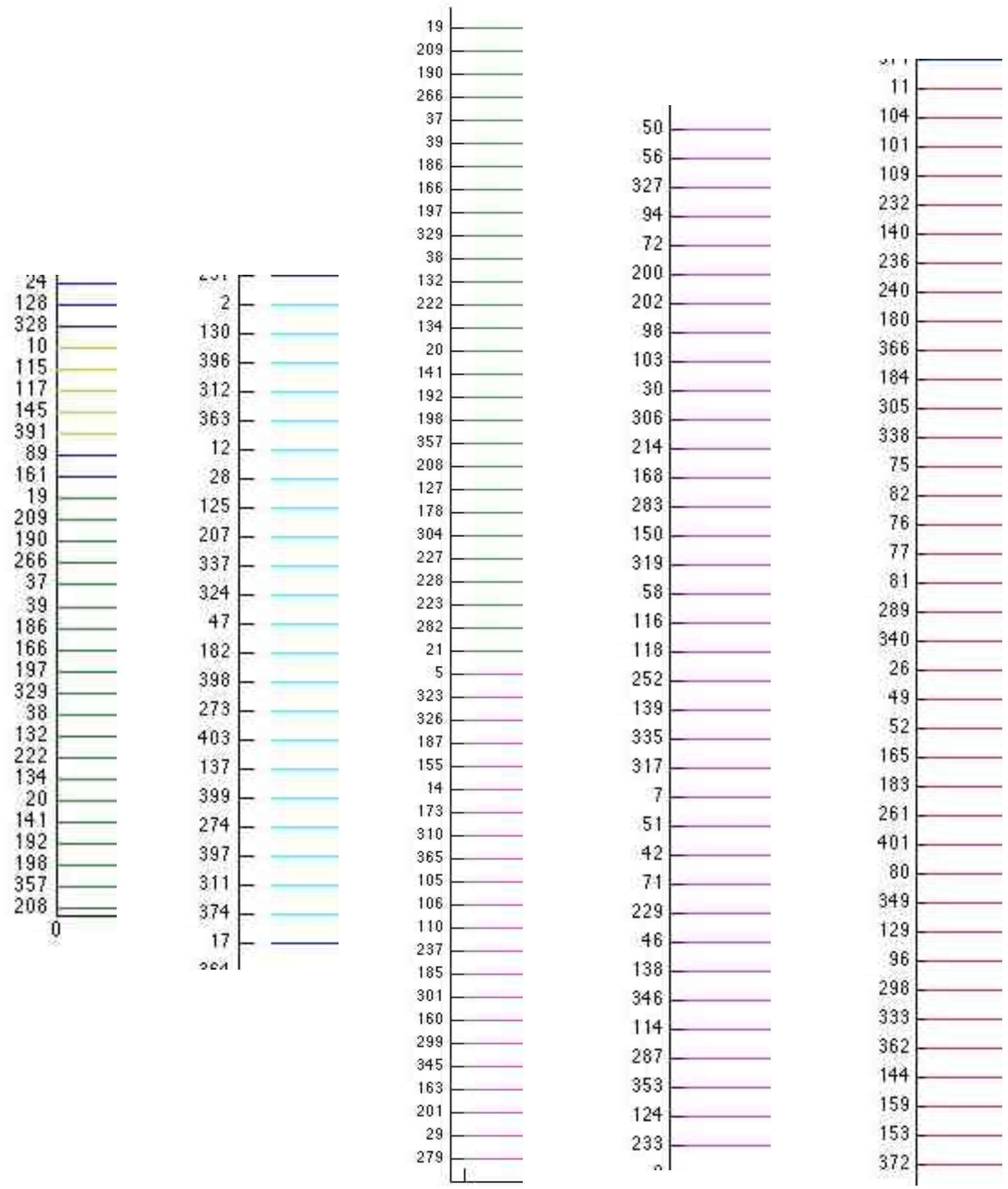

FBPase-1 a.

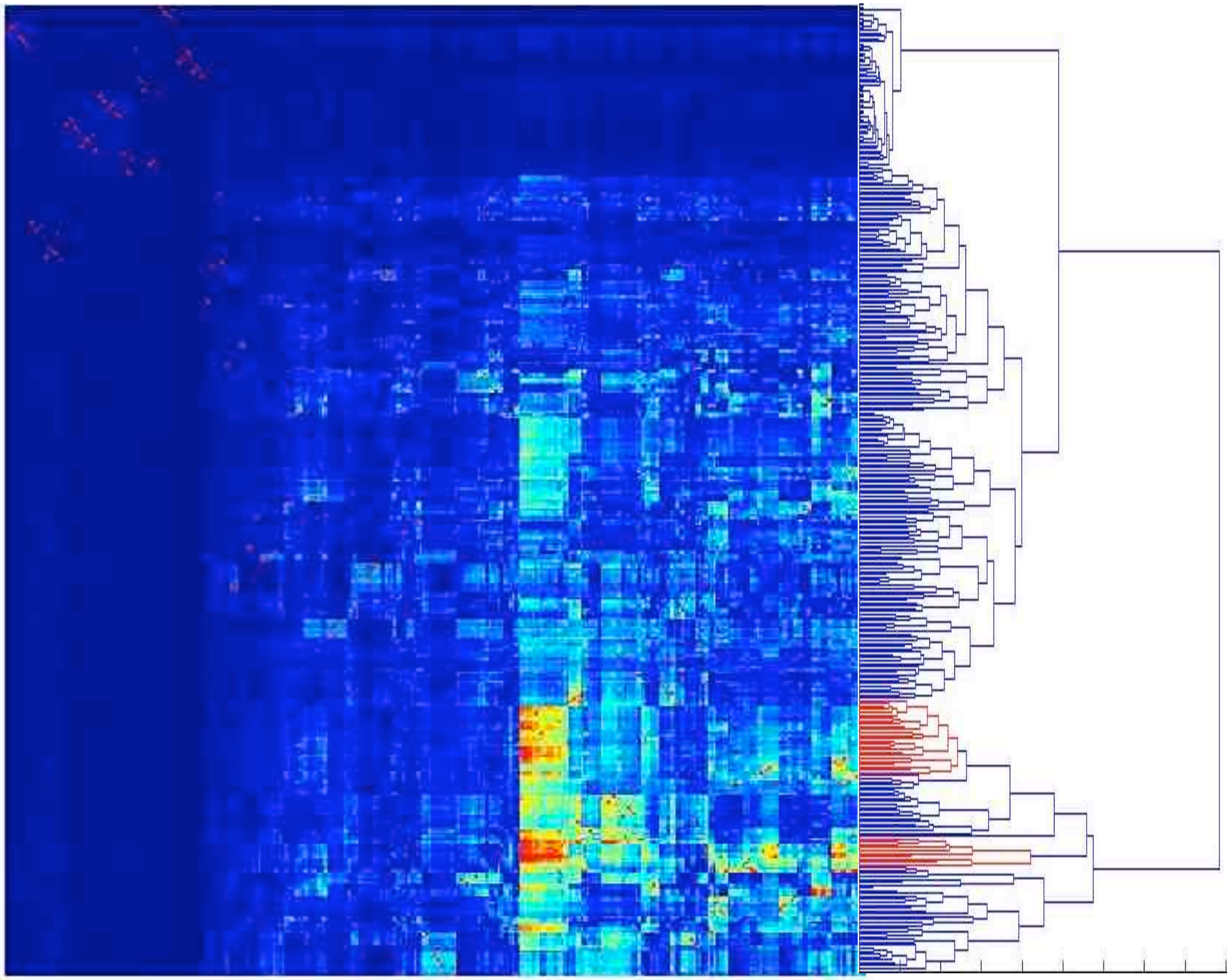

FBPase-1 b.

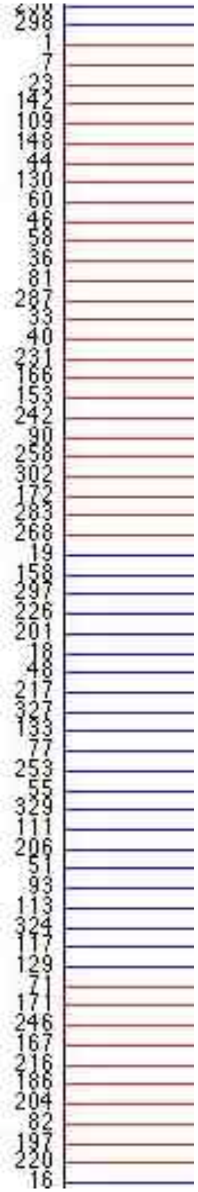
298
1
7
23
142
109
148
44
130
60
46
58
36
81
287
33
40
231
166
153
242
90
258
302
172
283
268
19
158
297
226
201
18
48
217
327
133
77
253
55
329
111
206
51
93
113
324
117
129
71
171
246
167
216
186
204
82
197
220
16

Phosphofructokinase a.

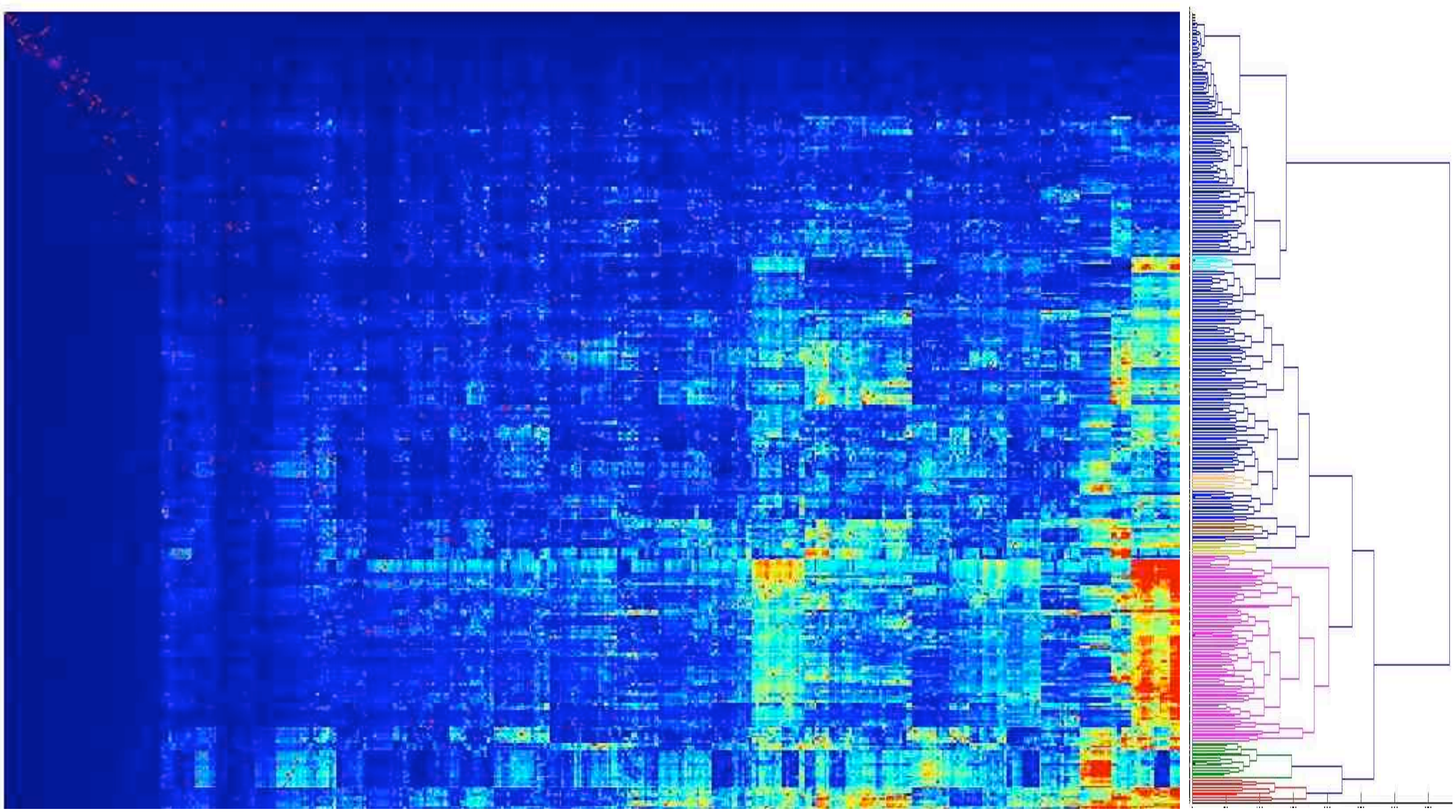

Phosphofructokinase b.

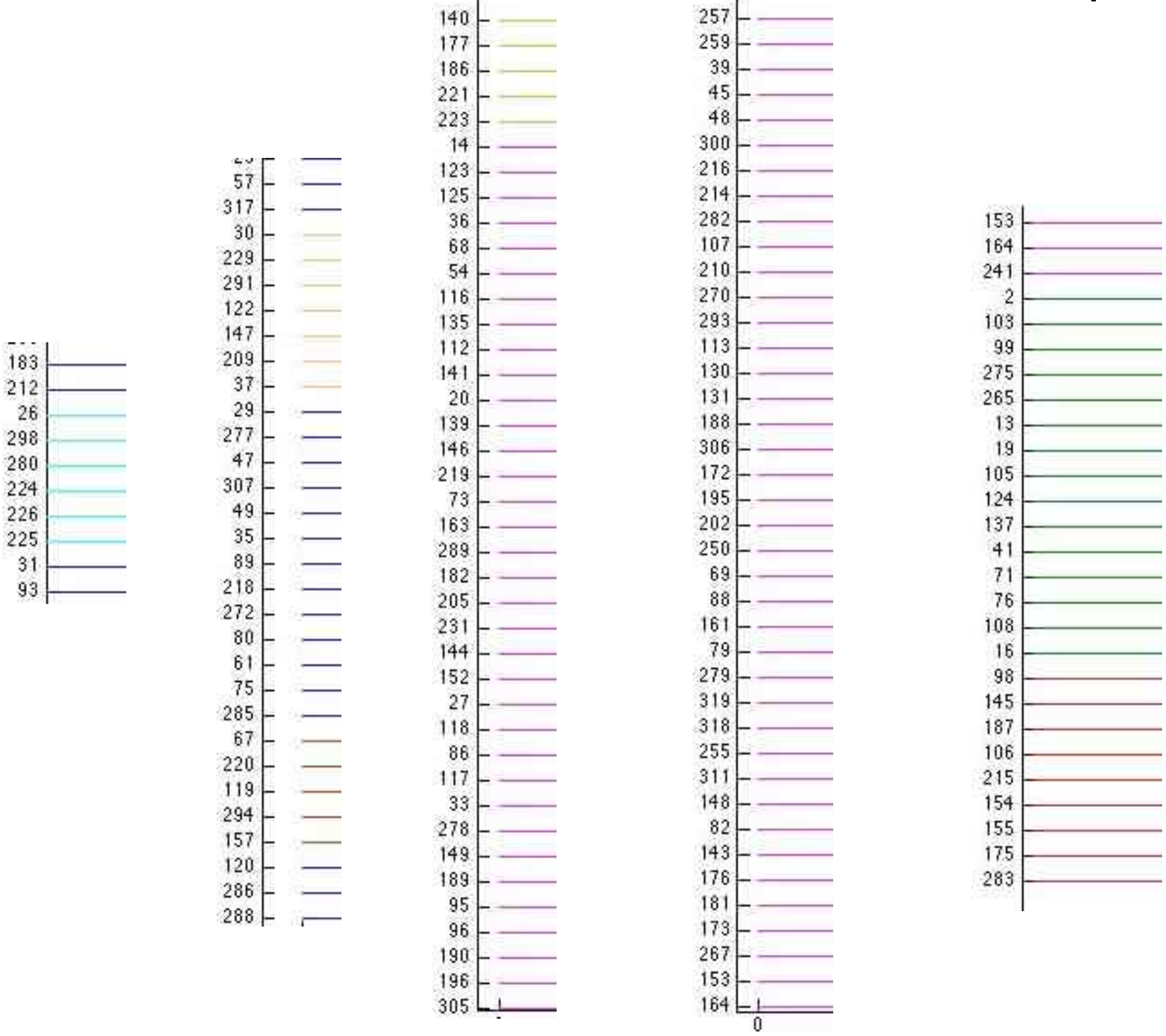

Aspartate transcarbamoylase, catalytic subunit a.

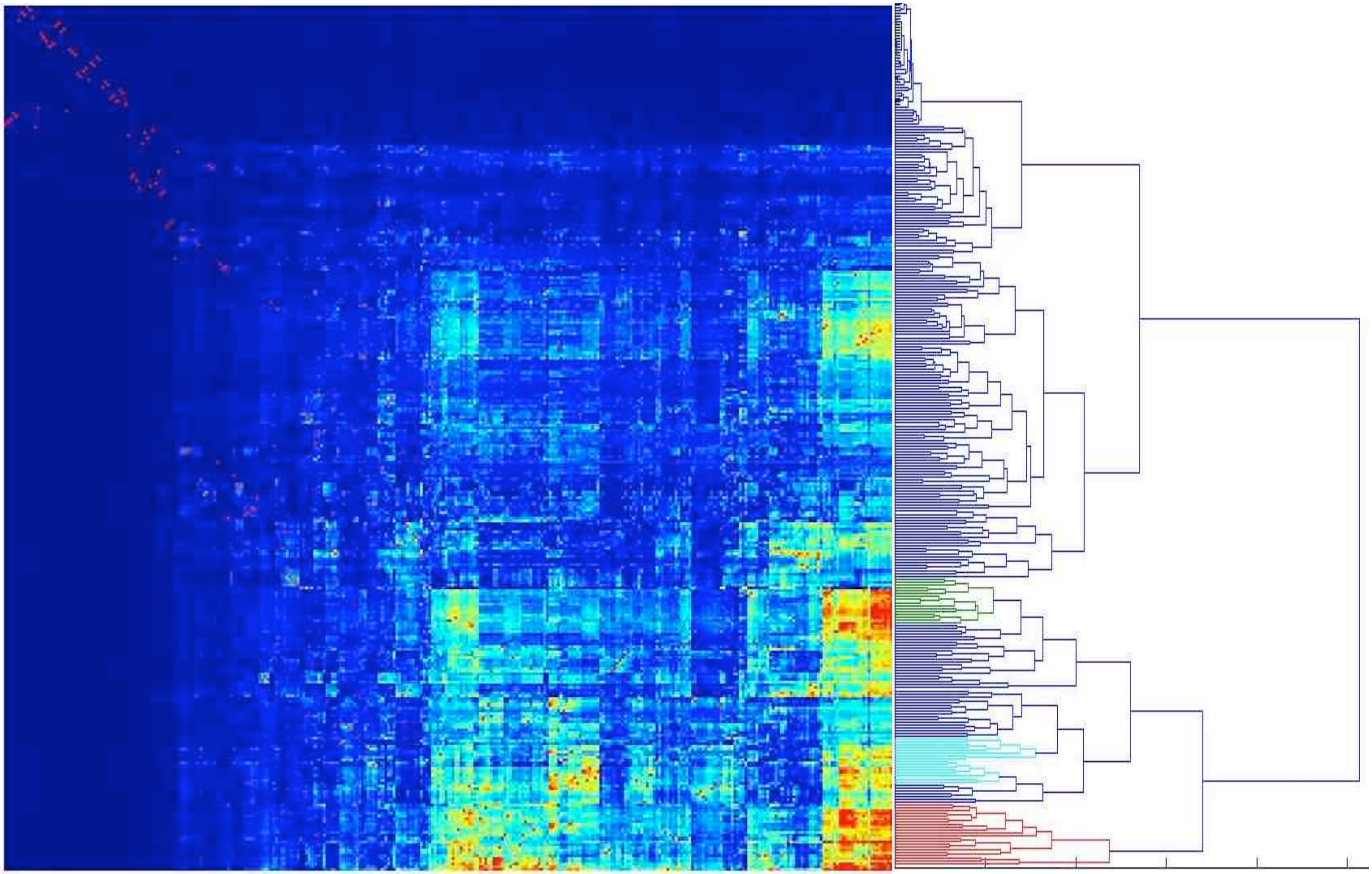

Aspartate transcarbamoylase, catalytic subunit b.

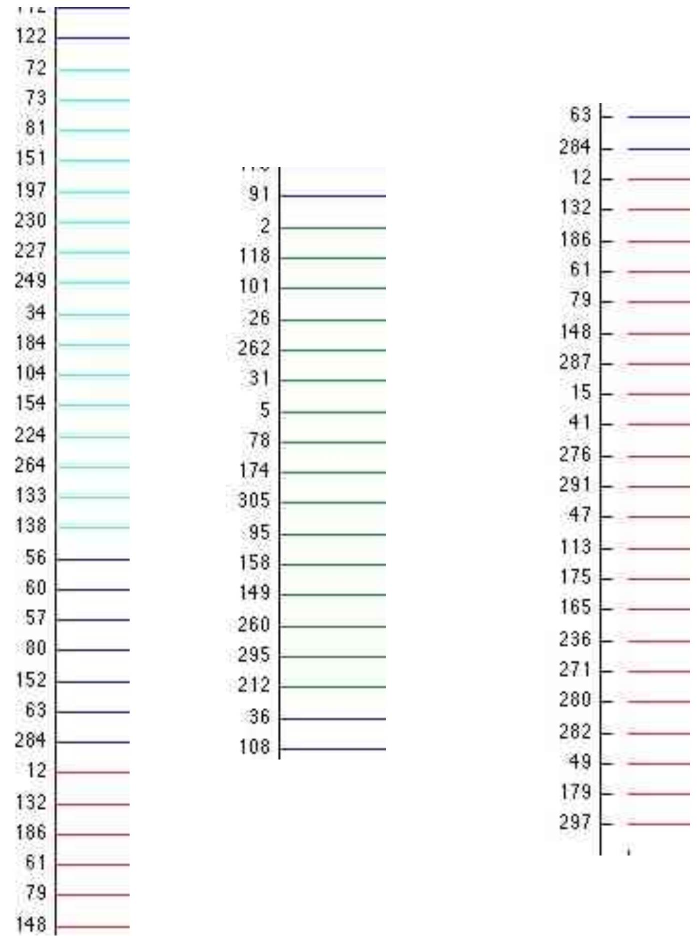

Glycogen phosphorylase a.

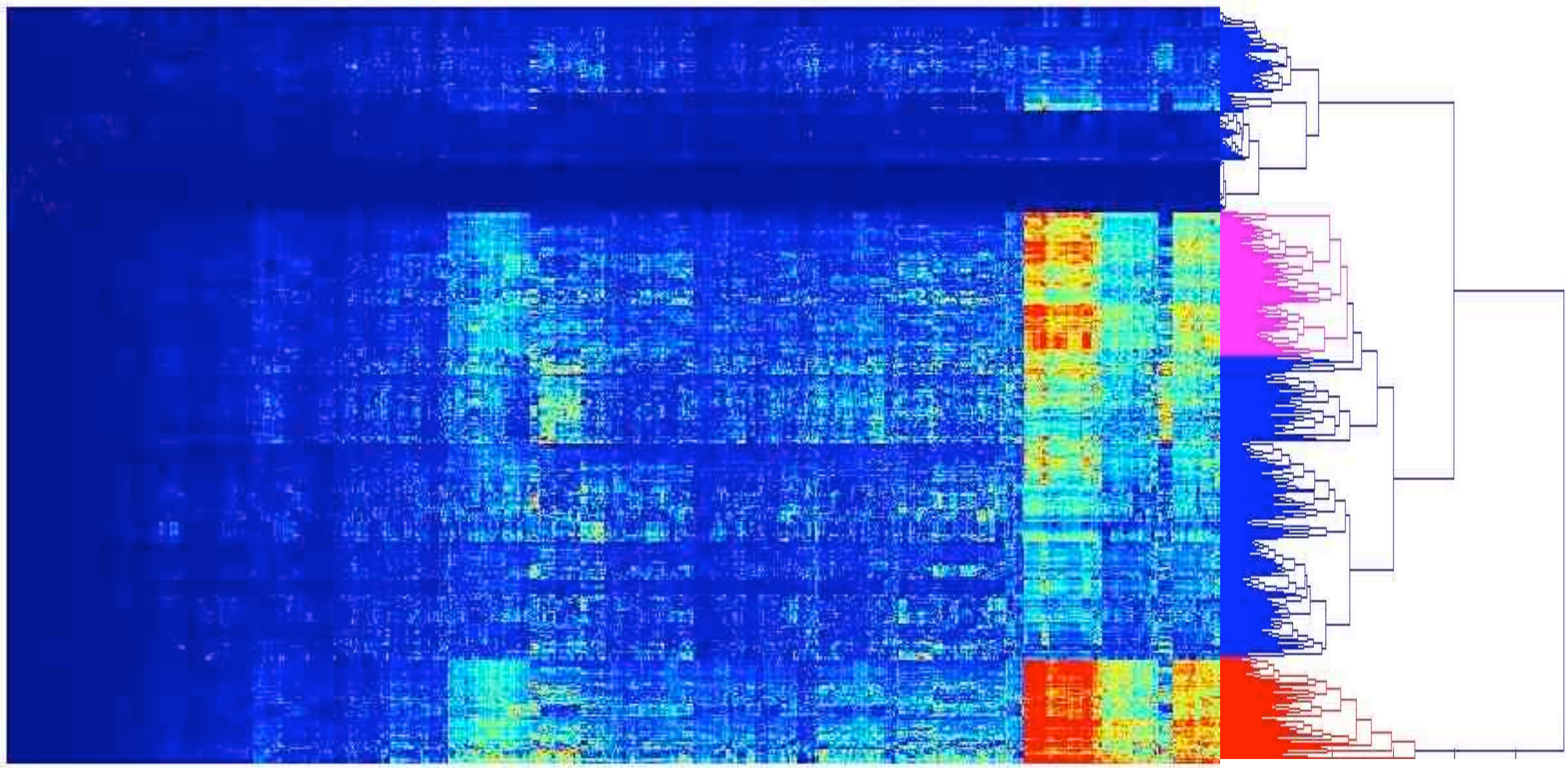

Glycogen phosphorylase b.

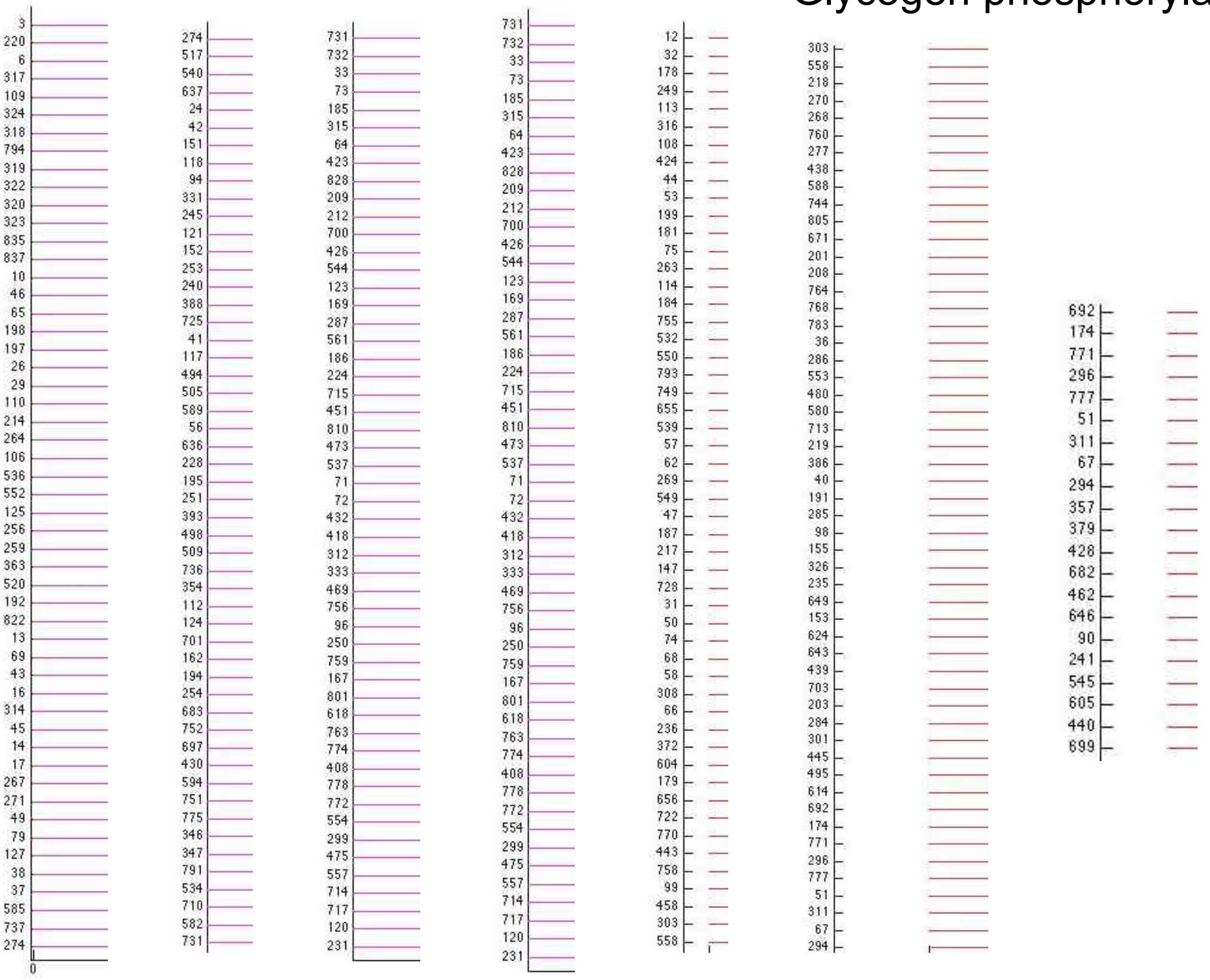

Cdc-42 a.

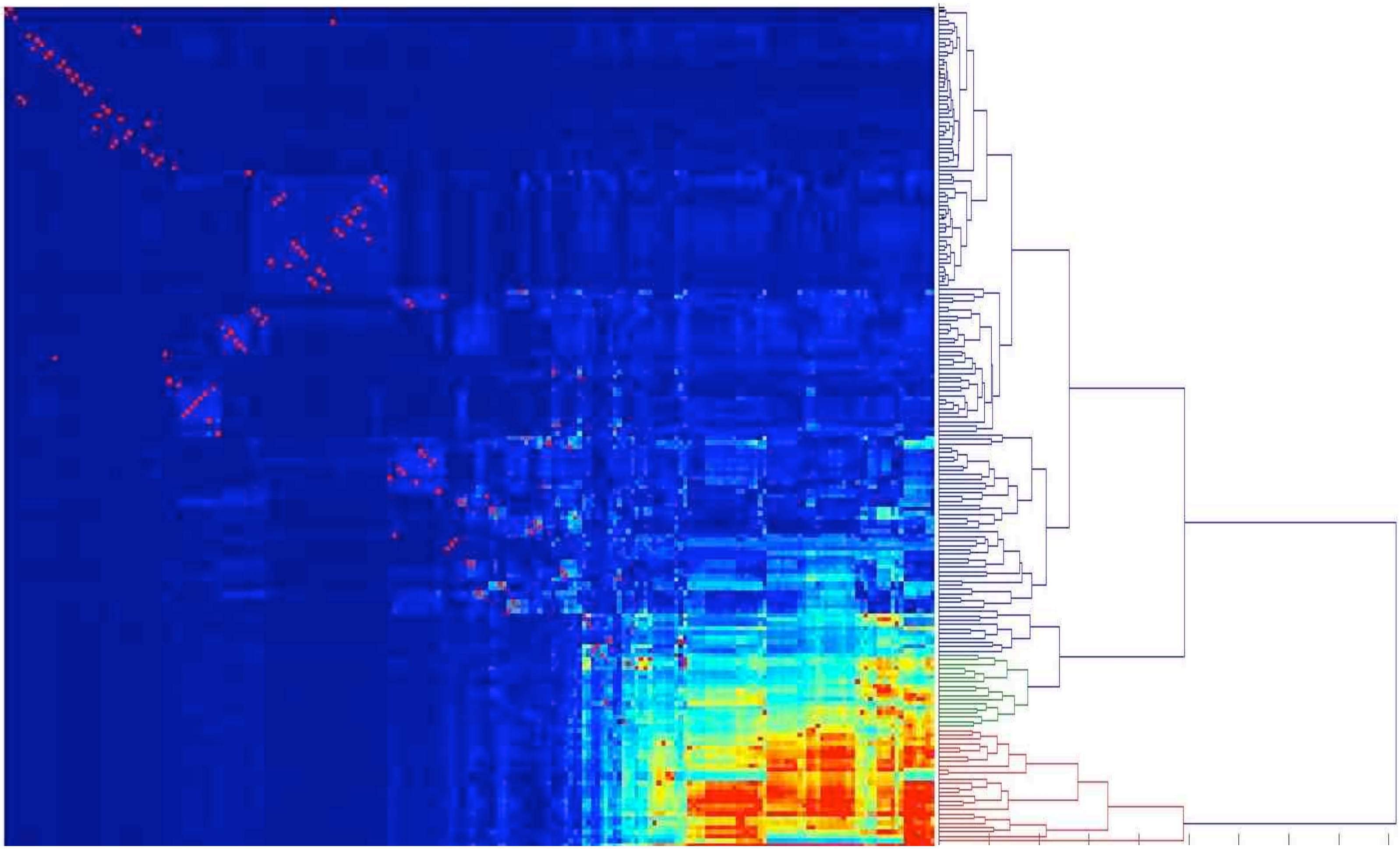

Cdc-42 b.

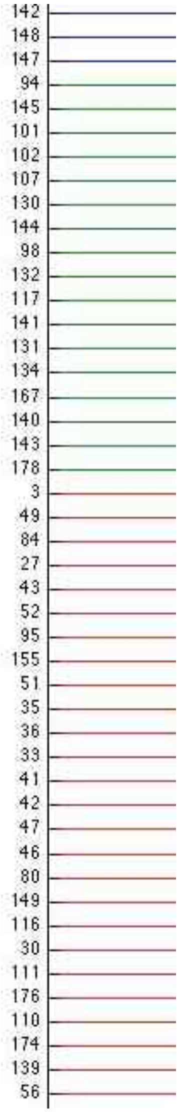
142
148
147
94
145
101
102
107
130
144
98
132
117
141
131
134
167
140
143
178
3
49
84
27
43
52
95
155
51
35
36
33
41
42
47
46
80
149
116
30
111
176
110
174
139
56

rhoA a.

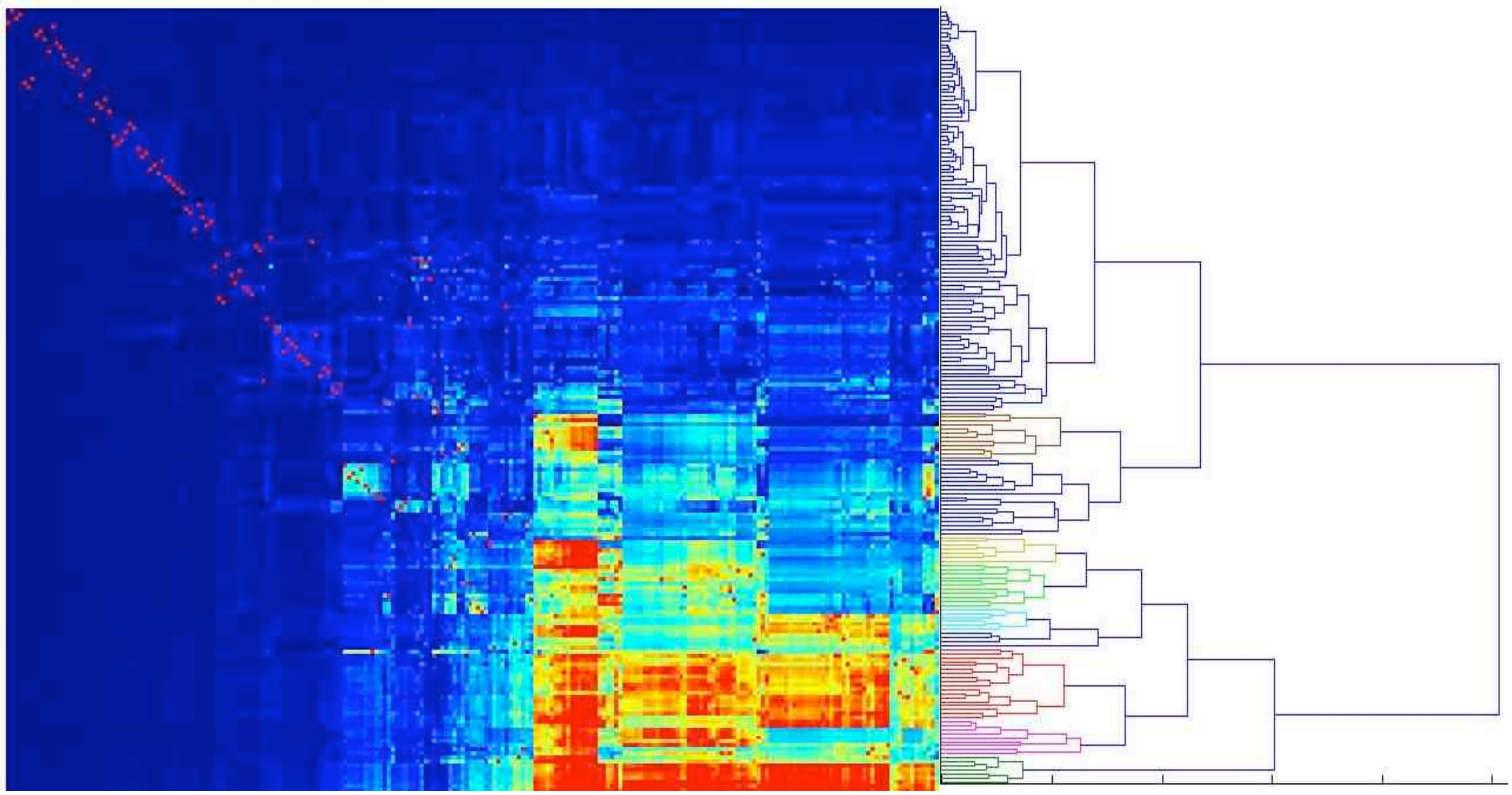

rhoA b.

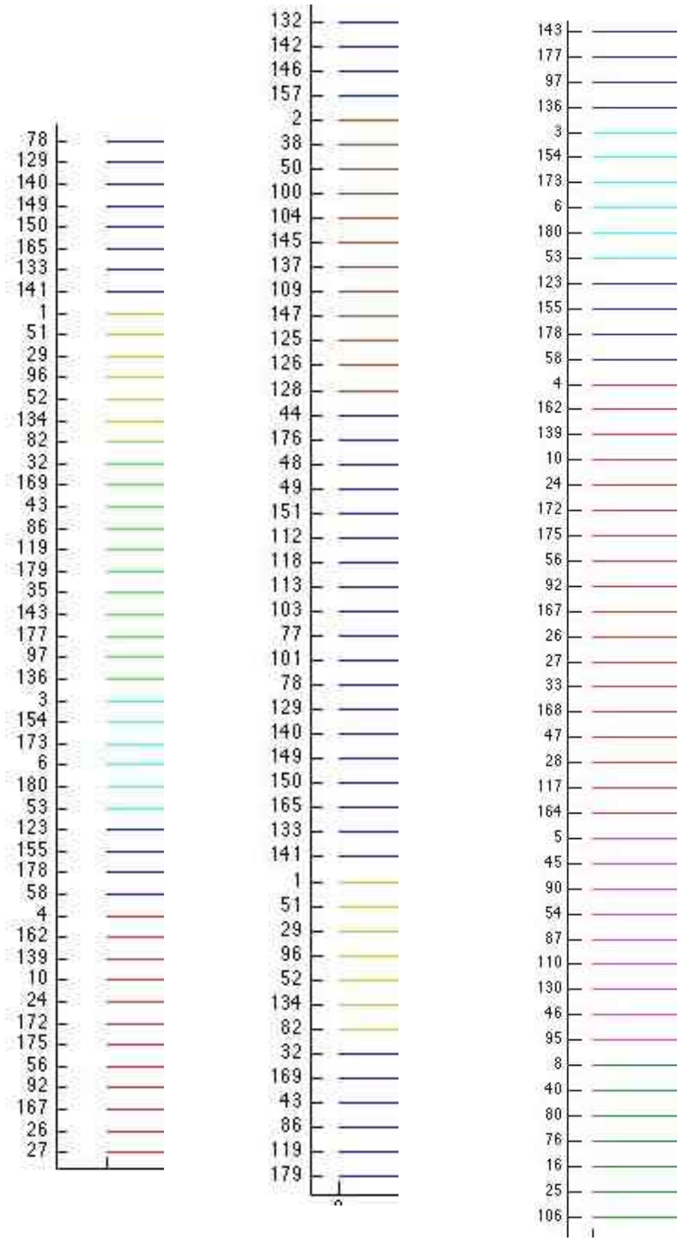
78 129 140 149 150 165 133 141 1 51 29 96 52 134 82 32 169 43 86 119 179 35 143 177 97 136 3 154 173 6 180 53 123 155 178 58 4 162 139 10 24 172 175 56 92 167 26 27
132 142 146 157 2 38 50 100 104 145 137 109 147 125 126 128 44 176 48 49 151 112 118 113 103 77 101 78 129 140 149 150 165 133 141 1 51 29 96 52 134 82 32 169 43 86 119 179
0
143 177 97 136 3 154 173 6 180 53 123 155 178 58 4 162 139 10 24 172 175 56 92 167 26 27 33 168 47 28 117 164 5 45 90 54 87 110 130 46 95 8 40 80 76 16 25 106

glutamate dehydrogenase a.

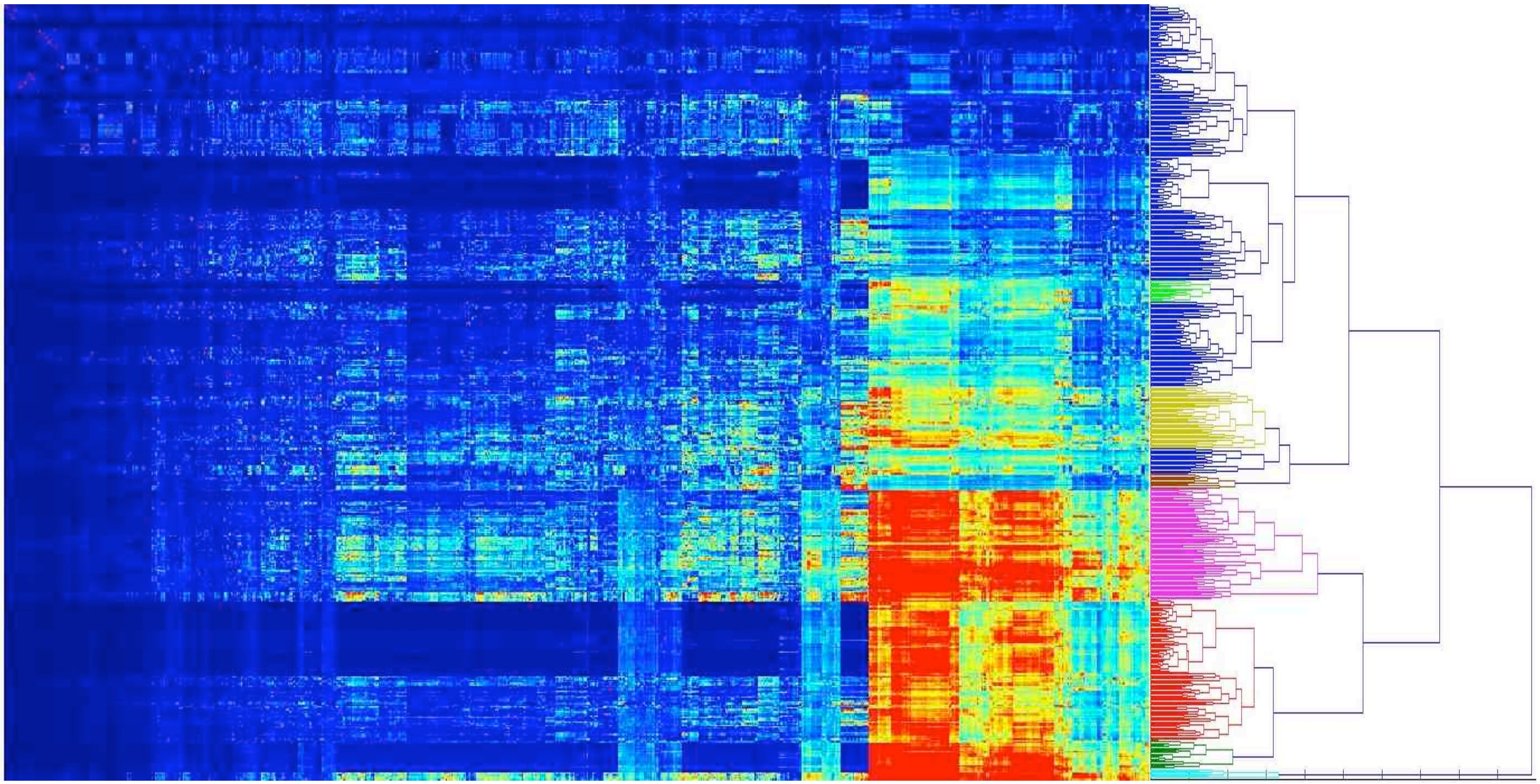

glutamate dehydrogenase b.

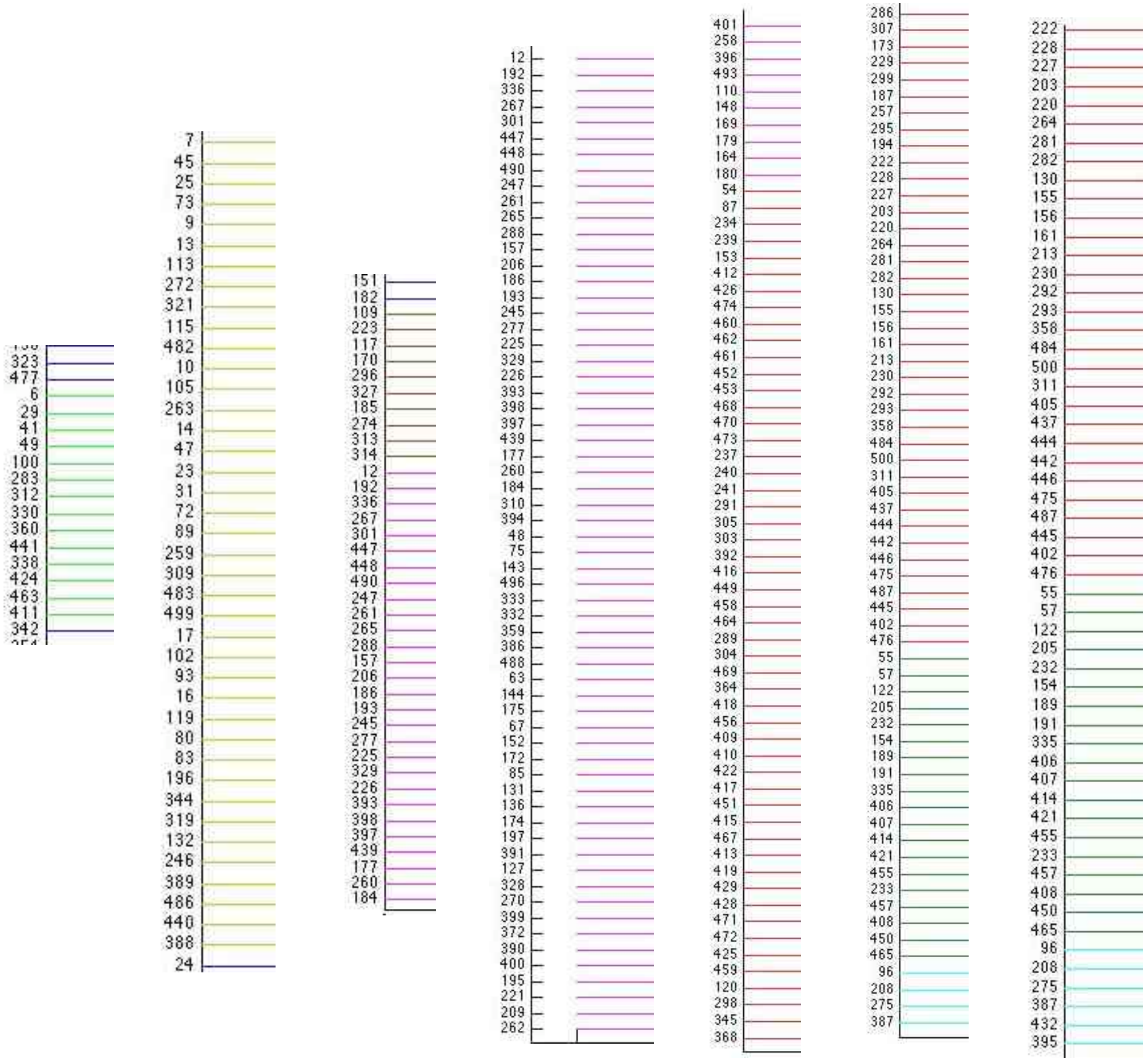

glucokinase a.

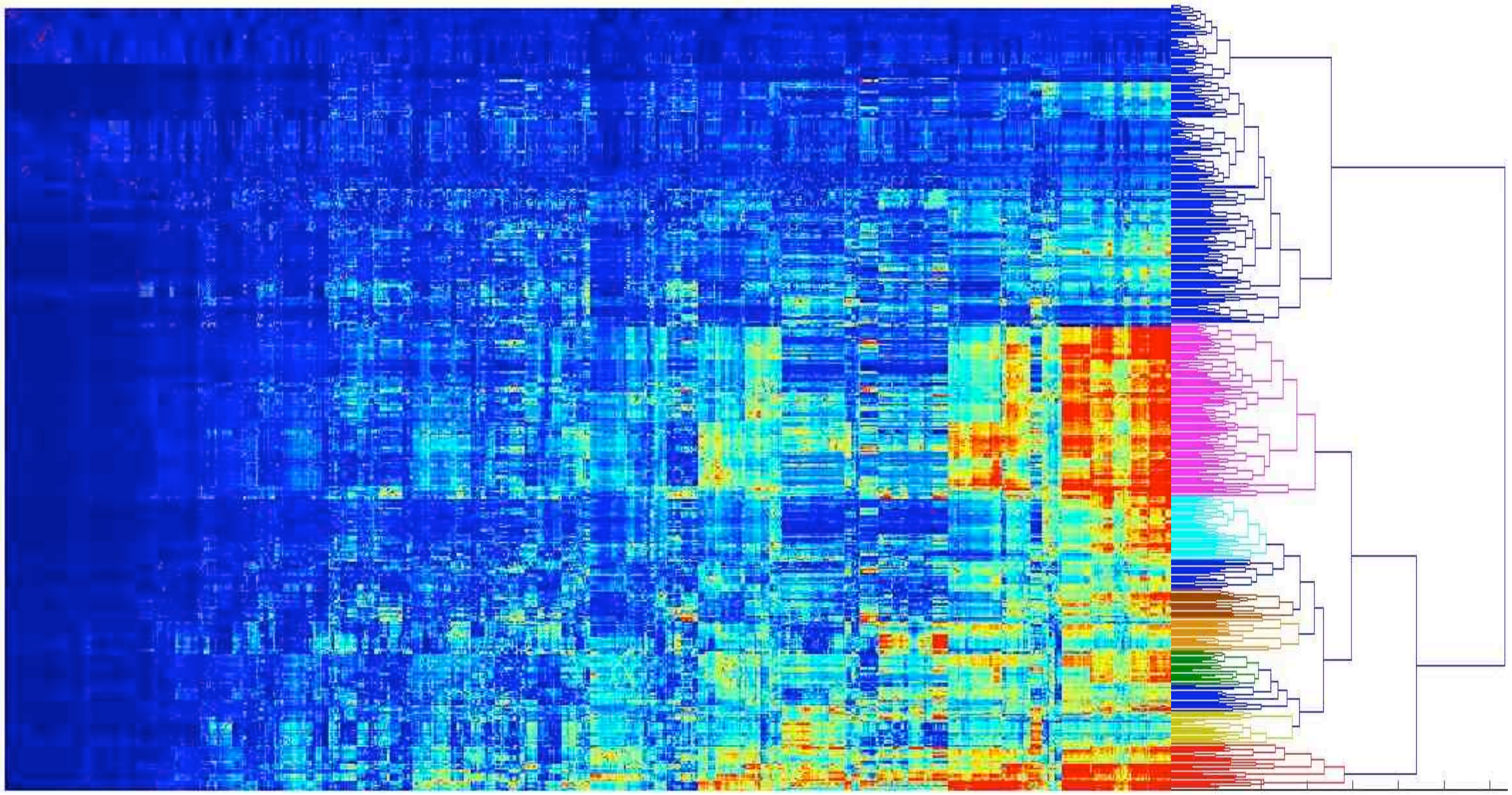

glucokinase b.

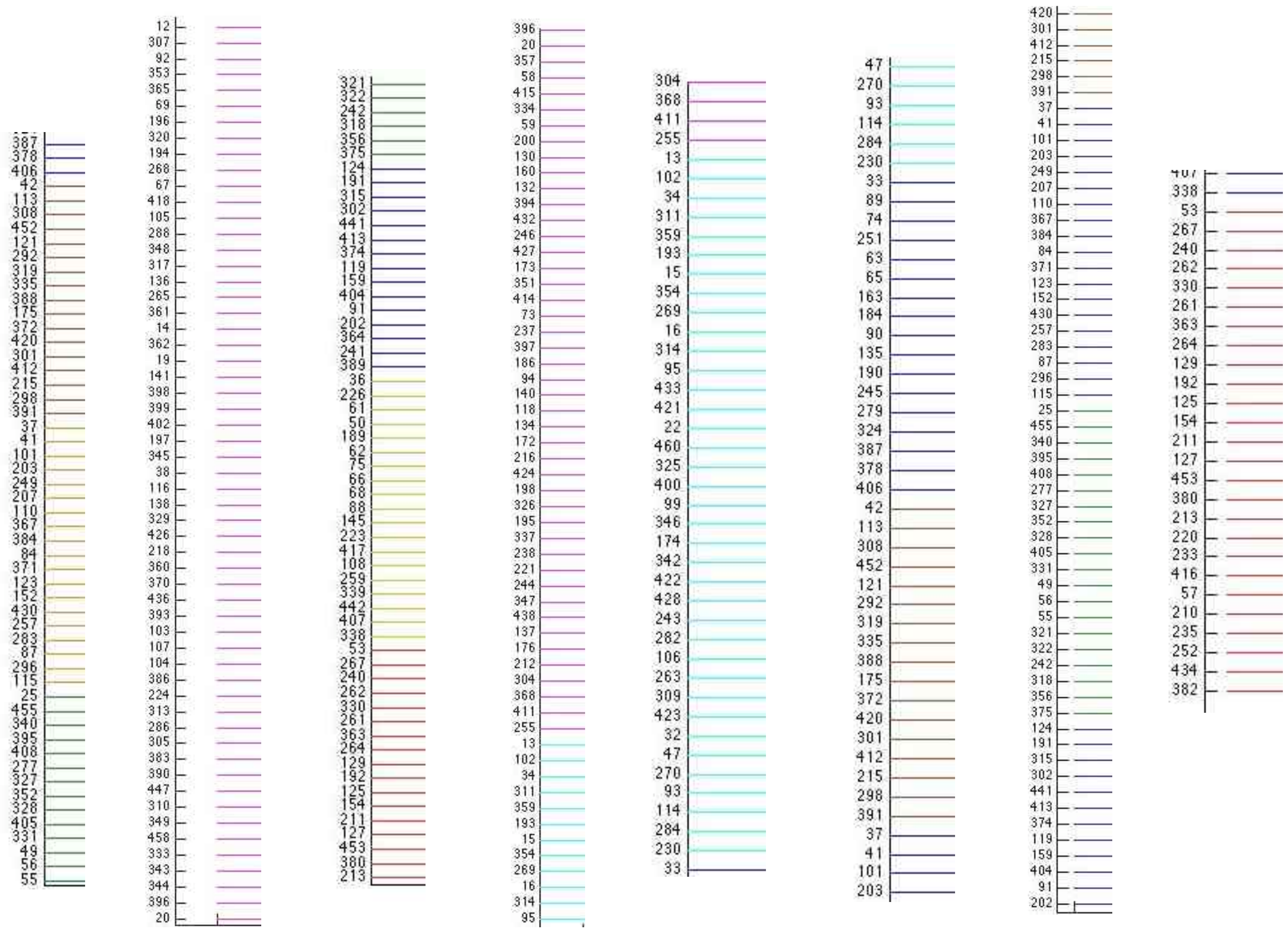

Lac repressor a.

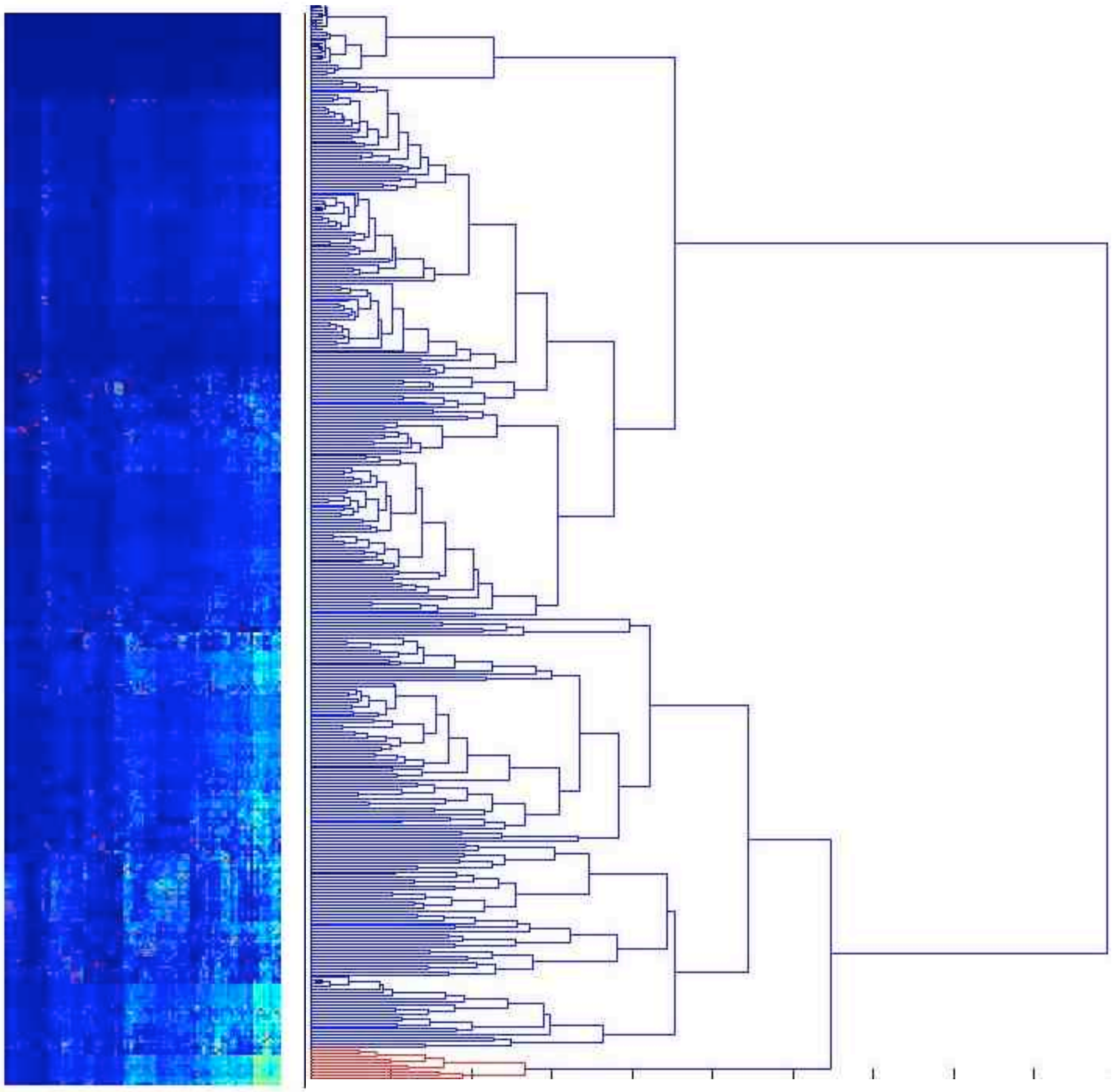

Lac repressor b.

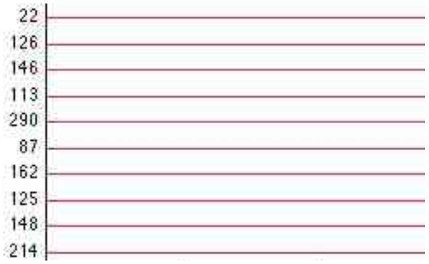

Thrombin, chain B a.

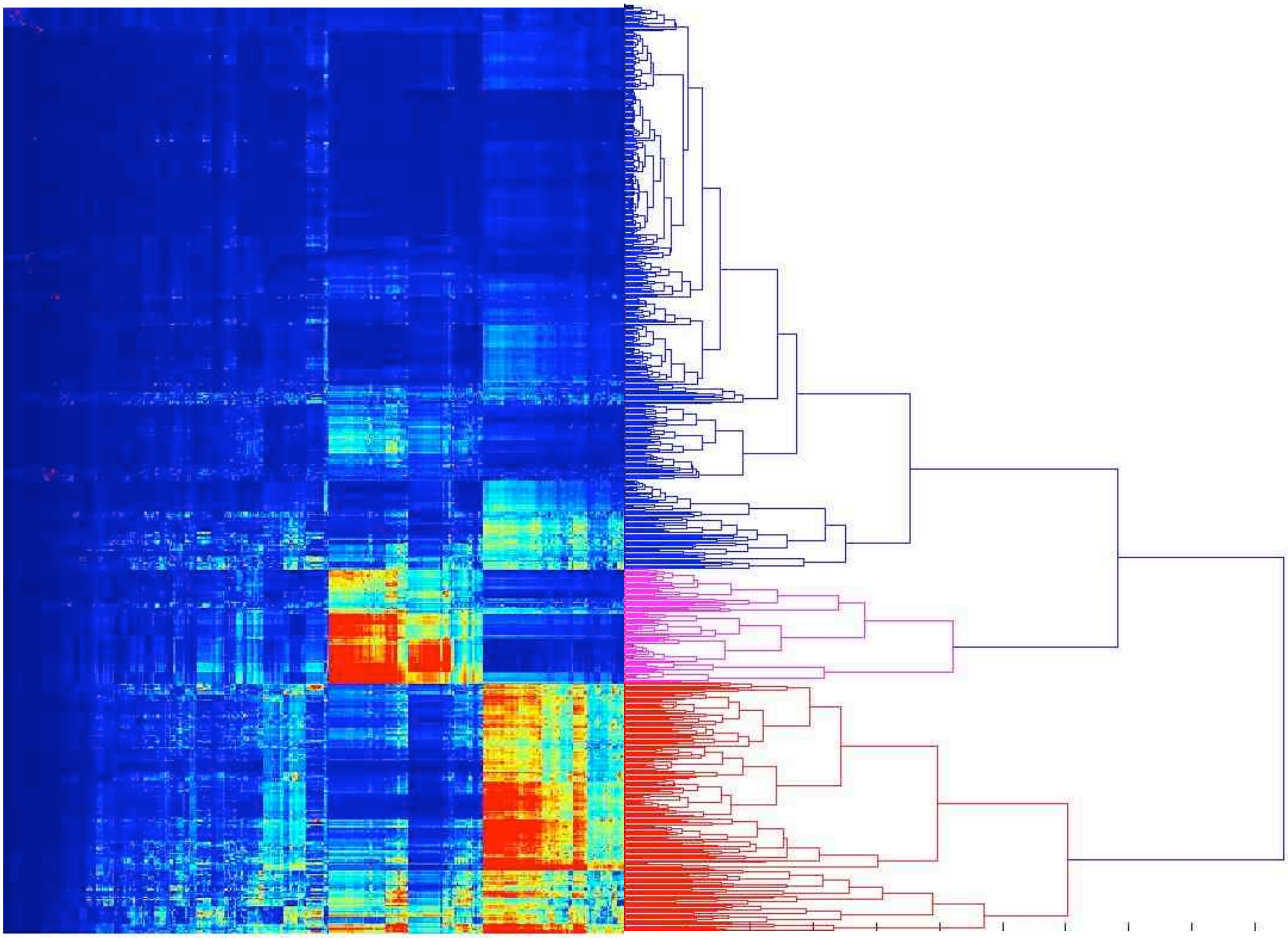

Thrombin, chain B b.

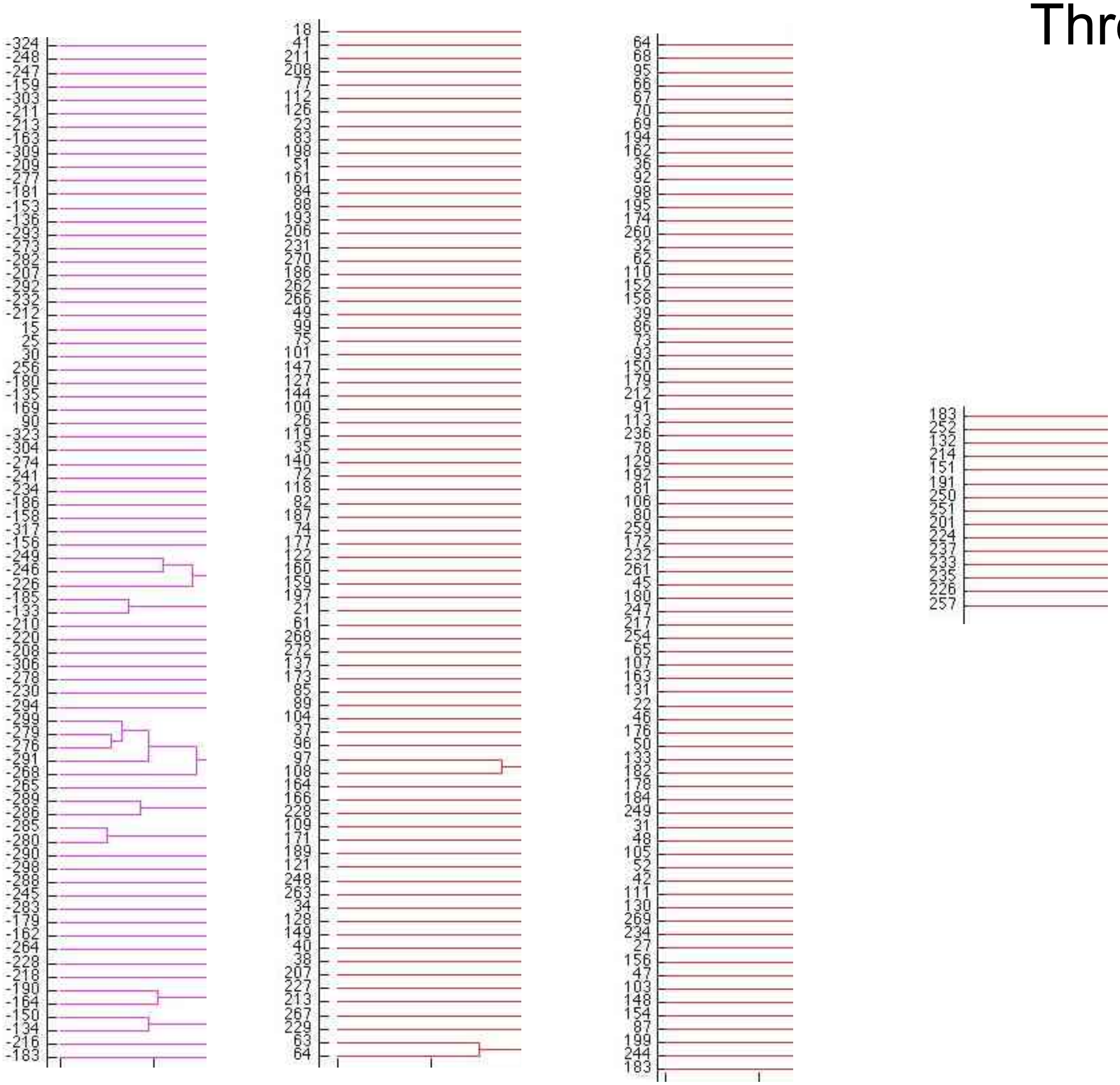

Figure S4. SCA data. Results for SCA are present for each protein from the training and independent data sets, except for myosin II where we relied on the previously published analysis by Yu et al. [F1]. a. Hierarchically clustered matrix of $\Delta\Delta G$ values and dendrogram where terminal branches correspond to residue indices of the protein sequence. Branches of the dendrogram corresponding to regions in the matrix containing clusters of high $\Delta\Delta G$ (regions with high fraction of points greater than or equal to 1.6 kT) are highlighted. The color scale is once displayed for CheY and applies to the subsequent protein systems. b. Magnification of the ends of the highlighted branches to display the residue indices, which are based on the numbering in the corresponding PDB file (except for thrombin, where negative numbers are for residues cleaved from prothrombin chain B and thrombin residues start at 1).

Supplemental References

F1. Yu H, Ma L, Yang Y, Cui Q (2007) Mechanochemical coupling in the myosin motor domain. II. Analysis of critical residues. Plos Computational Biology 3: 214-230.
