# Supplementary material for: Structure-Based Predictive Models for Allosteric Hot Spots
Source: PLoS Comput Biol. 2009 Oct 9;5(10):e1000531. doi: 10.1371/journal.pcbi.1000531 (PMC2748687; doi:10.1371/journal.pcbi.1000531)
Supplement: Table S1 — Training data set. Given are the protein name, the PDB ID of the inactive state, the PDB ID of the active state, the residue that was mutated, the reference(s) where the effect(s) of the mutation is (are) described, and, in the final column, details of the experiment(s) in which the mutation was characterized. In the final column, first the point mutation(s) is (are) given, and this is followed by a brief synopsis of the experimental results. Abbreviations used: wt = wild type; coef. = coefficient; repr. = repression. (0.11 MB RTF) [file pcbi.1000531.s005.rtf]

Table S1.  Training data set.  Given are the protein name, the PDB ID of the inactive state, the PDB ID of the active state, the residue that was mutated, the reference(s) where the effect(s) of the mutation is (are) described, and, in the final column, details of the experiment(s) in which the mutation was characterized.  In the final column, first the point mutation(s) is (are) given, and this is followed by a brief synopsis of the experimental results.  Abbreviations used:  wt=wild type; coef.=coefficient; repr.=repression.
						
HOTSPOTS
						
Protein system	PDB ID of inactive state	PDB ID of active state	Residue mutated	Reference	Experimental details	
						
CheY (monomer)	3chy	1fqw	asp 12	[T1]	D12A; alters beta4-alpha4 loop, which changes conf. upon binding Mg2+ (acts as switch region)	
			asp 13	[T2] 	D13K; constitutively tumbly in vivo (locked in active state)	
			thr 87	[T3]	T87I; smooth swimming, non-chemotactic; tyr 106 restricted to outside position (tyr 106 changes postion upon conf. change)	
			tyr 106	[T3] 	Y106W; more tumbly phenotype and impaired chemotaxis	
						
PurR (functionally dimeric)	1dbq	1wet	trp 147	[T4]	W147A, W147R:  superrepressor (in vivo repression assay based on lacZ reporter expression, 31.3, 33.6 fold repr. over control vs. 13.9 in wt)	
			glu 70 	[T5]	E70A:  superrepressor (in vivo assay based on expression of lacZ reporter; 51.8+/-3.5 fold repr. over purR- vs. 18.5+/-0.9 in wt); adopts conformation of holorepressor in absence of corepressor (circular dichroism)	
			arg 115	[T5] 	R115A:  repression reduced (lacZ reporter expression 1.5+/-0.2 fold repr. vs. 18.5+/-0.9 in wt)	
			ser 46	[T5] 	S46G:  repression reduced (lacZ reporter expression 1.5+/-0.3 fold repr. vs. 18.5+/-0.9 in wt)	
						
tet repressor (functionally dimeric)	2trt	1qpi	arg 49	[T6] 	R49G, R49Q:  in vivo inducibility 8.9+/-1.2 & 8.8+/-0.4, respectively(based on expression of lacZ reporter in presence of tetracycline (tc); percentages of control)	
			asp 53	[T6] 	D53G, D53Y:   in vivo inducibility 4.9+/-1.0, 9.0+/-0.9, respectively(based on expression of lacZ reporter in presence of tc;  percentages of control)	
			ala 56	[T6] 	A56V:   in vivo inducibility 8.2+/-0.7 (based on expression of lacZ reporter in presence of tc; percentage of control)	
			tyr 110	[T6] 	Y110C:   in vivo inducibility 8.1+/-0.9 (based on expression of lacZ reporter in presence of tc; percentage of control)	
			glu 114	[T6] 	E114G:   in vivo inducibility 5.8+/-0.9 (based on expression of lacZ reporter in presence of tc; percentage of control)	
			gly 143	[T6] 	G143D:   in vivo inducibility 5.7+/-0.8 (based on expression of lacZ reporter in presence of tc; percentage of control)	
			leu 146	[T6] 	L146F:   in vivo inducibility 2.1+/-0.2 (based on expression of lacZ reporter in presence of tc; percentage of control)	
			his 151	[T6] 	H151R:   in vivo inducibility 7.2+/-1.1 (based on expression of lacZ reporter in presence of tc; percentage of control)	
			arg 158 	[T6] 	R158G:   in vivo inducibility 5.5+/-0.5 (based on expression of lacZ reporter in presence of tc; percentage of control)	
			gly 138 	[T6] 	G138R:   in vivo inducibility 0.2+/-0.1 (based on expression of lacZ reporter in presence of tc; percentage of control); in vivo inducibility of 7.8+/-1.6 in absence of tc	
						
hemoglobin A (tetramer)	4hhb	1hho	beta asn 102	[T7]	N102A, N102Q, N102T:  reduced Hill coefficient w.r.t. O2 binding 1.06-1.1 in mutants vs. 2.8 in wt	
			beta cys 93	[T8]	C93A, C93G, C93M, C93L:  reduced Hill coefficient w.r.t. O2 binding (at physiological pH, 1.5-2.5 vs. 3.0 for wt	
			beta gln 131	[T9]	slightly lower cooperativity	
			alpha leu 29	[T10] 	L29F: reduced Hill coeff. (2.4 vs. 3.0 in wt at pH 7.4)	
			beta trp 37	[T11] 	reduced Hill coeff. for all mutants (Gill Cell O2 binding exp) W37Y: 2.4; W37A:  1.4; W37G:  1.2; W37E:  1.1; 3.2 in wt	
						
d-3-phosphoglycerate dehydrogenase (functionally tetrameric)	1psd	1yba	gly 336	[T12];  [T13]	G336V, G336L:  increased IC-50 for serine from 10 in wt to 142 and 110, respectively;  G336V:  Hill coef. Of 1.82+/-0.9 vs. 2.02+/-0.07 in wt	
			arg 405	[T14]	reduced Hill coeff.:  1.42 +/- 0.09 vs. 2.02 +/- 0.07 in wt.	
			arg 407	[T14]	reduced Hill coeff.:  1.21 +/- 0.08 vs. 2.02 +/- 0.07 in wt.	
						
phosphofructokinase (functionally tetrameric)	6pfk	4pfk	arg 152 	[T15]	R152A:  increased Hill coeff. 5.6 +/- 0.5 vs. 3.7 +/- 0.3 in wt.;  R152K increased Hill coeff. 7.5 +/- 0.5 vs. 3.7 +/- 0.3 in wt. 	
			leu 178	[T16] 	L178W:  abolition of response to allosteric effectors PEP or GDP (based on equilibrium binding curves 	
						
FBPase-1 (functionally tetrameric)	1eyj	1eyi	ala 54	[T17]	A54L:  50-fold increase in IC-50 for AMP (62+/-1 vs. 1.23+/-0.04 in wt), but Hill coeff. intact	
			phe 6	[T18] 	F6W:  IC-50 for AMP increased over wt (2.87+/-0.09 vs. 1.61+/-0.05 in wt)	
			ile 10	[T18]	I10A:  IC-50 for allosteric effector AMP is increased ~1500 fold over wt	
			lys 50	[T19]	K50P:  lower Hill coeff. for Mg2+ (1.45+/-0.08 vs. 1.9+/-0.1 in wt), increased IC-50 for AMP (13400+/-20 vs. 1.61+/-0.05 in wt)	
			ala 51	[T19]	A51P:  increased IC-50 for AMP (460+/-7 vs. 1.61+/-0.05 in wt)	
			asn 64	[T20]	N64A:  abolished cooperativity for AMP (n=1 vs. n=2 in wt.)	
			arg 49	[T21]	R49C, R49D, R49L:  abolished cooperativity for AMP (n=1 vs. n=2 in wt.)	
			lys 42	[T22]	K42T:  abolished cooperativity for AMP (n=1 vs. n=2 in wt.)	
			ile 190	[T22]	I190T:  abolished cooperativity for AMP (n=1 vs. n=2 in wt.)	
			gly 191	[T22]	G191A:  abolished cooperativity for AMP (n=1 vs. n=2 in wt.)	
			arg 22	[T23]	R22M:  abolished cooperativity for AMP (n=1 vs. n=2 in wt.)	
						
aspartate transcarbamoylase (functionally dodecameric)	1rac	1d09	glu 239 (catalytic subunit)	[T24]	E239Q:  Hill coeff. for aspartate of 1.0 vs. 2.2 in wt.	
			asp 236 (catalytic chain)	[T25]	D236A:  Hill coeff. for aspartate of 1.0 vs. 2.6+/-0.5 in wt	
			asp 162 (catalytic subunit)	[T26]	D162A:  Hill coeff for aspartate of 1.0 vs. 2.3 in wt	
						
glycogen phosphorylase 
(T: dimer; 
R: tetramer)	1gpb	7gpb	asn 270	[T27]	N270A:  Hill coef. for AMP of 1.27+/-0.06 vs. 1.57+/-0.04 in wt	
			arg 277	[T27]	R277A:  Hill coef. for AMP of 1.27+/-0.07 vs. 1.57+/-0.04 in wt	
						


NON-HOTSPOTS
						
Protein system	PDB ID of inactive state	PDB ID of active state	Residue mutated	Reference	Experimental details	
						
tet repressor (functionally dimeric)	2trt	1qpi	his 139	[T6]	H139P:  80+/-0.3% of wt inducibility (in vivo; based on lacZ reporter)	
			ala 173	[T6]	A173V:  87.4+/-1.4% of wt inducibility (in vivo; based on lacZ reporter)	
			ala 154	[T6]	A154T, A154V, A154Y:  80.7+/-1.6, 71.3+/-0.1, 75.9+/-2.0% of wt inducibility, respectively (in vivo; based on lacZ reporter)	
			gly 189	[T6]	G189S:  98.9+/-4.1% of wt inducibility (in vivo; based on lacZ reporter)	
						
d-3-phosphoglycerate dehydrogenase (functionally tetrameric)	1psd	1yba	glu 387	[T14]	E387A:  no significant effect on Hill coeff. (2.26 +/- 0.11 vs. 2.02 +/- 0.07 wt)	
			gly 337	[T12]; [T13]	G337A, G337V, G337L:  IC-50 for serine of 12, 15, 8 vs. 10 in wt; G337V did not have reduced Hill coef. 2.24 +/- 0.14 vs. 2.02+/-0.07 in wt	
						
FBPase-1 (functionally tetrameric)	1eyj	1eyi	arg 15	[T23]	R15A:  Hill coeff. for AMP is 2 (as in wt.), Hill coeff. for Mg2+ 1.90+/-0.04 vs. 1.97+/-0.12 in wt.	
			glu 19	[T23]	E19Q:  Hill coeff. for AMP is 2 (as in wt.), Hill coeff. for Mg2+ 1.78+/-0.08 vs. 1.97+/-0.12 in wt.	
			gln 32	[T22]	Q32L:  Hill coeff. for AMP =2 as in wt	
						
phosphofructokinase (functionally tetrameric)	6pfk	4pfk	val 246	[T28]	V246T:  Hill coeff. for F6P of 3.2+/-0.1 vs. 4.0 in wt 	
			arg 63	[T28]	R63S:  Hill coeff. for F6P of 3.4+/-0.4 vs. 4.0 in wt	
						
rho A (monomer)	1ftn	1a2b	tyr 42	[T29]	Y42K:  no change in sensitivity to the GEF (guanine nucleotide exchange factor) Lbc based on [T3H]GDP/GTP exchange assay	
			glu 64	[T29]	E64K:  no change in sensitivity to the GEF (guanine nucleotide exchange factor) Lbc based on [T3H]GDP/GTP exchange assay	
			asp 67	[T29]	D67K:  no change in sensitivity to the GEF (guanine nucleotide exchange factor) Lbc based on [T3H]GDP/GTP exchange assay	
			arg 70	[T29]	R70A:  no change in sensitivity to the GEF (guanine nucleotide exchange factor) Lbc based on [T3H]GDP/GTP exchange assay	
			ser 73	[T29]	S73A:  no change in sensitivity to the GEF (guanine nucleotide exchange factor) Lbc based on [T3H]GDP/GTP exchange assay	
			tyr 74	[T29]	Y74A:  no change in sensitivity to the GEF (guanine nucleotide exchange factor) Lbc based on [T3H]GDP/GTP exchange assay	
			asp 78	[T29]	D78K:  no change in sensitivity to the GEF (guanine nucleotide exchange factor) Lbc based on [T3H]GDP/GTP exchange assay	
			ile 80	[T29]	I80L:  no change in sensitivity to the GEF (guanine nucleotide exchange factor) Lbc based on [T3H]GDP/GTP exchange assay	
			asp 28	[T29]	D28N:   no change in sensitivity to the GEF (guanine nucleotide exchange factor) Lbc based on [T3H]GDP/GTP exchange assay	
			met 82	[T29]	M82V:   no change in sensitivity to the GEF (guanine nucleotide exchange factor) Lbc based on [T3H]GDP/GTP exchange assay	
			thr 37	[T29]	T37A:  no change in sensitivity to the GEF (guanine nucleotide exchange factor) Dbl based on [T3H]GDP/GTP exchange assay	
			lys 27	[T29]	K27T:  no change in sensitivity to the GEF (guanine nucleotide exchange factor) Dbl based on [T3H]GDP/GTP exchange assay	
			asp 76	[T29]	D76Q:  no change in sensitivity to the GEF (guanine nucleotide exchange factor) Dbl based on [T3H]GDP/GTP exchange assay	
			phe 39	[T29]	F39E:  no change in sensitivity to the GEF (guanine nucleotide exchange factor) Dbl based on [T3H]GDP/GTP exchange assay	
						
cdc 42 (monomer)	1an0	1nf3	tyr 40	[T29]	Y40K:  no change in sensitivity to the GEF (guanine nucleotide exchange factor) cdc-24 based on [T3H]GDP/GTP exchange assay	
			gln 61 (here leu)	[T29]	Q61L:   no change in sensitivity to the GEF (guanine nucleotide exchange factor) cdc-24 based on [T3H]GDP/GTP exchange assay	
			asp 63	[T29]	D63H:   no change in sensitivity to the GEF (guanine nucleotide exchange factor) cdc-24 based on [T3H]GDP/GTP exchange assay	
			asp 65	[T29]	D65K:   no change in sensitivity to the GEF (guanine nucleotide exchange factor) cdc-24 based on [T3H]GDP/GTP exchange assay	
			arg 66	[T29]	R66D:   no change in sensitivity to the GEF (guanine nucleotide exchange factor) cdc-24 based on [T3H]GDP/GTP exchange assay	
			arg 68	[T29]	R68A:   no change in sensitivity to the GEF (guanine nucleotide exchange factor) cdc-24 based on [T3H]GDP/GTP exchange assay	
			pro 69	[T29]	P69A:   no change in sensitivity to the GEF (guanine nucleotide exchange factor) cdc-24 based on [T3H]GDP/GTP exchange assay	
			phe 78	[T29]	F78L:   no change in sensitivity to the GEF (guanine nucleotide exchange factor) cdc-24 based on [T3H]GDP/GTP exchange assay	
			thr 25	[T29]	T25K:   no change in sensitivity to the GEF (guanine nucleotide exchange factor) cdc-24 based on [T3H]GDP/GTP exchange assay	
			asn 26	[T29]	N26D:  no change in sensitivity to the GEF (guanine nucleotide exchange factor) cdc-24 based on [T3H]GDP/GTP exchange assay	
			val 85	[T29]	V85D:  no change in sensitivity to the GEF (guanine nucleotide exchange factor) cdc-24 based on [T3H]GDP/GTP exchange assay	
			ser 88	[T29]	S88D:  no change in sensitivity to the GEF (guanine nucleotide exchange factor) cdc-24 based on [T3H]GDP/GTP exchange assay	
			phe 90	[T29]	F90L:  no change in sensitivity to the GEF (guanine nucleotide exchange factor) cdc-24 based on [T3H]GDP/GTP exchange assay	
			val 98	[T29]	V98E:  no change in sensitivity to the GEF (guanine nucleotide exchange factor) cdc-24 based on [T3H]GDP/GTP exchange assay	
						
hemoglobin A (tetramer)	4hhb	1hho	beta tyr 35	[T30]	Y35F, Y35A:  Hill coef. of 2.4+/-0.2, 2.7+/-0.2 vs. 2.8+/-0.2	
			alpha thr 67	[T31]	T67V:  Hill coef. of 2.6-3.0 vs. 3.0-3.2 in wt.	
			beta ser 72	[T31]	S72A:  Hill coef. of 2.6-3.0 vs. 3.0-3.2 in wt.	
			beta leu 105	[T32]	L105W:  Hill coeff. of 2.3 vs. 2.7 in wt	
			beta phe 41	[T33]	F41Y:  Hill coeff. for O2 of 2.1 at half saturation vs. 2.3 in wt.	
			beta asn 108	[T34]	N108Q:  Hill coef. of 3.10 vs. 3.28 in wt	
						
						
glycogen phosphorylase 
(T: dimer,
R: tetramer)	1gpb	7gpb	lys 11	[T35]	K11E, K11A:  Hill coef. for the effector AMP of 1.49+/-0.01 and 1.45+/-0.045, respectively vs. Hill coef. of 1.39+/-0.16 in wt.	
			glu 501	[T35]	E501A:  Hill coef. for AMP of 1.55 vs. 1.39+/-0.16 in wt.	
						
purR repressor (functionally dimeric)	1dbq	1wet	gln 113	[T5]	Q113G:  no significant effect on repressor function (18.5+/-4.2 fold repr. vs. 18.5+/-0.9 in wt)	
			ala 49	[T5]	A49G:  no significant effect on repressor function (16.2+/-3.1 fold repr. vs. 18.5+/-0.9 in wt)	

Supplemental References

T1. Sola M, Lopez-Hernandez E, Cronet P, Lacroix E, Serrano L, et al. (2000) Towards understanding a molecular switch mechanism: Thermodynamic and crystallographic studies of the signal transduction protein CheY. Journal of Molecular Biology 303: 213-225.
T2. Jiang MY, Bourret RB, Simon MI, Volz K (1997) Uncoupled phosphorylation and activation in bacterial chemotaxis - The 2.3 angstrom structure of an aspartate to lysine mutant at position 13 of CheY. Journal of Biological Chemistry 272: 11850-11855.
T3. Zhu XY, Rebello J, Matsumura P, Volz K (1997) Crystal structures of CheY mutants Y106W and T871/Y106W - CheY activation correlates with movement of residue 106. Journal of Biological Chemistry 272: 5000-5006.
T4. Huffman JL, Lu F, Zalkin H, Brennan RG (2002) Role of residue 147 in the gene regulatory function of the Escherichia coli purine repressor. Biochemistry 41: 511-520.
T5. Lu F, Brennan RG, Zalkin H (1998) Escherichia coli purine repressor: Key residues for the allosteric transition between active and inactive conformations and for interdomain signaling. Biochemistry 37: 15680-15690.
T6. Muller G, Hecht B, Helbl V, Hinrichs W, Saenger W, et al. (1995) Characterization of Noninducible Tet Repressor Mutants Suggests Conformational-Changes Necessary for Induction. Nature Structural Biology 2: 693-703.
T7. Kwiatkowski LD, Hui HL, Karasik E, Colby JE, Noble RW (2007) Mutations of the beta N102 residue of HbA not only inhibit the ligand-linked T to R-e state transition, but also profoundly affect the properties of the T state itself. Biochemistry 46: 2037-2049.
T8. Cheng Y, Shen TJ, Simplaceanu V, Ho C (2002) Ligand binding properties and structural studies of recombinant and chemically modified hemoglobins altered at beta 93 cysteine. Biochemistry 41: 11901-11913.
T9. Chang CK, Simplaceanu V, Ho C (2002) Effects of amino acid substitutions at beta 131 on the structure and properties of hemoglobin: Evidence for communication between alpha(1)beta(1)- and alpha(1)beta(2)-subunit interfaces. Biochemistry 41: 5644-5655.
T10. Jeong ST, Ho NT, Hendrich MP, Ho C (1999) Recombinant hemoglobin(alpha 29leucine -> phenylalanine, alpha 96valine -> tryptophan, beta 108asparagine -> lysine) exhibits low oxygen affinity and high cooperativity combined with resistance to autoxidation. Biochemistry 38: 13433-13442.
T11. Kiger L, Klinger AL, Kwiatkowski LD, De Young A, Doyle ML, et al. (1998) Thermodynamic studies on the equilibrium properties of a series of recombinant beta W37 hemoglobin mutants. Biochemistry 37: 4336-4345.
T12. Grant GA, Xu XL, Hu ZQ (2000) Role of an interdomain Gly-Gly sequence at the regulatory-substrate domain interface in the regulation of Escherichia coli. D-3-phosphoglycerate dehydrogenase. Biochemistry 39: 7316-7319.
T13. Grant GA, Hu ZQ, Xu XL (2001) Amino acid residue mutations uncouple cooperative effects in Escherichia coli D-3-phosphoglycerate dehydrogenase. Journal of Biological Chemistry 276: 17844-17850.
T14. Grant GA, Hu ZQ, Xu XL (2001) Specific interactions at the regulatory domain-substrate binding domain interface influence the cooperativity of inhibition and effector binding in Escherichia coli D-3-phosphoglycerate dehydrogenase. Journal of Biological Chemistry 276: 1078-1083.
T15. Auzat I, Lebras G, Garel JR (1995) Hypercooperativity Induced by Interface Mutations in the Phosphofructokinase from Escherichia-Coli. Journal of Molecular Biology 246: 248-253.
T16. Serre MC, Teschner W, Garel JR (1990) Specific Suppression of Heterotropic Interactions in Phosphofructokinase by the Mutation of Leucine-178 into Tryptophan. Journal of Biological Chemistry 265: 12146-12148.
T17. Iancu CV, Mukund S, Fromm HJ, Honzatko RB (2005) R-state AMP complex reveals initial steps of the quaternary transition of fructose-1,6-bisphosphatase. Journal of Biological Chemistry 280: 19737-19745.
T18. Nelson SW, Kurbanov FT, Honzatko RB, Fromm HJ (2001) The N-terminal segment of recombinant porcine fructose-1,6-bisphosphatase participates in the allosteric regulation of catalysis. Journal of Biological Chemistry 276: 6119-6124.
T19. Nelson SW, Choe JY, Honzatko RB, Fromm HJ (2000) Mutations in the hinge of a dynamic loop broadly influence functional properties of fructose-1,6-bisphosphatase. Journal of Biological Chemistry 275: 29986-29992.
T20. Kurbanov FT, Choe JY, Honzatko RB, Fromm HJ (1998) Directed mutations in the poorly defined region of porcine liver fructose 1,6-bispbosphatase significantly affect catalysis and the mechanism of AMP inhibition. Journal of Biological Chemistry 273: 17511-17516.
T21. Shyur LF, Poland BW, Honzatko RB, Fromm HJ (1997) Major changes in the kinetic mechanism of AMP inhibition and AMP cooperativity attend the mutation of Arg(49) in fructose-1,6-bisphosphatase. Journal of Biological Chemistry 272: 26295-26299.
T22. Shyur LF, Aleshin AE, Honzatko RB, Fromm HJ (1996) Biochemical properties of mutant and wild-type fructose-1,6-bisphosphatases are consistent with the coupling of intra- and intersubunit conformational changes in the T- and R-state transition. Journal of Biological Chemistry 271: 33301-33307.
T23. Shyur LF, Aleshin AE, Honzatko RB, Fromm HJ (1996) Site-directed mutagenesis of residues at subunit interfaces of porcine fructose-1,6-bisphosphatase. Journal of Biological Chemistry 271: 3005-3010.
T24. Ladjimi MM, Kantrowitz ER (1988) A Possible Model for the Concerted Allosteric Transition in Escherichia-Coli Aspartate-Transcarbamylase as Deduced from Site-Directed Mutagenesis Studies. Biochemistry 27: 276-283.
T25. Chan RS, Sakash JB, Macol CP, West JA, Tsuruta H, et al. (2002) The role of intersubunit interactions for the stabilization of the T state of Escherichia coli aspartate transcarbamoylase. Journal of Biological Chemistry 277: 49755-49760.
T26. Fetler L, Tauc P, Baker DP, Macol CP, Kantrowitz ER, et al. (2002) Replacement of Asp-162 by Ala prevents the cooperative transition by the substrates while enhancing the effect of the allosteric activator ATP on E-coli aspartate transcarbamoylase. Protein Science 11: 1074-1081.
T27. Buchbinder JL, Guinovart JJ, Fletterick RJ (1995) Mutations in Paired Alpha-Helices at the Subunit Interface of Glycogen-Phosphorylase Alter Homotropic and Heterotropic Cooperativity. Biochemistry 34: 6423-6432.
T28. Kundrot CE, Evans PR (1991) Designing an Allosterically Locked Phosphofructokinase. Biochemistry 30: 1478-1484.
T29. Li R, Zheng Y (1997) Residues of the Rho family GTPases Rho and Cdc42 that specify sensitivity to Dbl-like guanine nucleotide exchange factors. Journal of Biological Chemistry 272: 4671-4679.
T30. Kavanaugh JS, Weydert JA, Rogers PH, Arnone A, Hui HL, et al. (2001) Site-directed mutations of human hemoglobin at residue 35 beta: A residue at the intersection of the alpha 1 beta 1, alpha 1 beta 2, and at alpha 1 alpha 2 interfaces. Protein Science 10: 1847-1855.
T31. Tsai CH, Simplaceanu V, Ho NT, Shen TJ, Wang DJ, et al. (2003) Site mutations disrupt inter-helical H-bonds (alpha 14W-alpha 67T and beta 15W-beta 72S) involved in kinetic steps in the hemoglobin R -> T transition without altering the free energies of oxygenation. Biophysical Chemistry 100: 131-142.
T32. Fang TY, Simplaceanu V, Tsai CH, Ho NT, Ho C (2000) An additional H-bond in the alpha(1)beta(2) interface as the structural basis for the low oxygen affinity and high cooperativity of a novel recombinant hemoglobin (beta L105W). Biochemistry 39: 13708-13718.
T33. Baudin-Creuza V, Vasseur-Godbillon C, Griffon N, Kister J, Kiger L, et al. (1999) Additive effects of beta chain mutations in low oxygen affinity hemoglobin beta F41Y,K66T. Journal of Biological Chemistry 274: 25550-25554.
T34. Tsai CH, Fang TY, Ho NT, Ho C (2000) Novel recombinant hemoglobin, rHb (beta N108Q), with low oxygen affinity, high cooperativity, and stability against autoxidation. Biochemistry 39: 13719-13729.
T35. Biorn AC, Graves DJ (2001) The amino-terminal tail of glycogen phosphorylase is a switch for controlling phosphorylase conformation, activation, and response to ligands. Biochemistry 40: 5181-5189.
T36. Fang J, Hsu BYL, MacMullen CM, Poncz M, Smith TJ, et al. (2002) Expression, purification and characterization of human glutamate dehydrogenase (GDH) allosteric regulatory mutations. Biochemical Journal 363: 81-87.
T37. Stanley CA, Fang J, Kutyna K, Hsu BYL, Ming JE, et al. (2000) Molecular basis and characterization of the hyperinsulinism/hyperammonemia syndrome - Predominance of mutations in exons 11 and 12 of the glutamate dehydrogenase gene. Diabetes 49: 667-673.
T38. De Lonlay P, Benelli C, Fouque F, Ganguly A, Aral B, et al. (2001) Hyperinsulinism and hyperammonemia syndrome: report of twelve unrelated patients. Pediatr Res 50: 353-357.
T39. Zaganas I, Plaitakis A (2002) Single amino acid substitution (G456A) in the vicinity of the GTP binding domain of human housekeeping glutamate dehydrogenase markedly attenuates GTP inhibition and abolishes the cooperative behavior of the enzyme. J Biol Chem 277: 26422-26428.
T40. Zhang J, Li C, Chen K, Zhu W, Shen X, et al. (2006) Conformational transition pathway in the allosteric process of human glucokinase. Proc Natl Acad Sci U S A 103: 13368-13373.
T41. Moukil MA, Veiga-da-Cunha M, Van Schaftingen E (2000) Study of the regulatory properties of glucokinase by site-directed mutagenesis: conversion of glucokinase to an enzyme with high affinity for glucose. Diabetes 49: 195-201.
T42. Davis EA, Cuesta-Munoz A, Raoul M, Buettger C, Sweet I, et al. (1999) Mutants of glucokinase cause hypoglycaemia- and hyperglycaemia syndromes and their analysis illuminates fundamental quantitative concepts of glucose homeostasis. Diabetologia 42: 1175-1186.
T43. Mahalingam B, Cuesta-Munoz A, Davis EA, Matschinsky FM, Harrison RW, et al. (1999) Structural model of human glucokinase in complex with glucose and ATP: implications for the mutants that cause hypo- and hyperglycemia. Diabetes 48: 1698-1705.
T44. Markiewicz P, Kleina LG, Cruz C, Ehret S, Miller JH (1994) Genetic studies of the lac repressor. XIV. Analysis of 4000 altered Escherichia coli lac repressors reveals essential and non-essential residues, as well as "spacers" which do not require a specific sequence. J Mol Biol 240: 421-433.
T45. Suckow J, Markiewicz P, Kleina LG, Miller J, Kisters-Woike B, et al. (1996) Genetic studies of the Lac repressor. XV: 4000 single amino acid substitutions and analysis of the resulting phenotypes on the basis of the protein structure. J Mol Biol 261: 509-523.
T46. Sasaki N, Ohkura R, Sutoh K (2003) Dictyostelium myosin II mutations that uncouple the converter swing and ATP hydrolysis cycle. Biochemistry 42: 90-95.
T47. Tsiavaliaris G, Fujita-Becker S, Batra R, Levitsky DI, Kull FJ, et al. (2002) Mutations in the relay loop region result in dominant-negative inhibition of myosin II function in Dictyostelium. EMBO Rep 3: 1099-1105.
T48. Suzuki Y, Ohkura R, Sugiura S, Yasuda R, Kinoshita K, Jr., et al. (1997) Modulation of actin filament sliding by mutations of the SH2 cysteine in Dictyostelium myosin II. Biochem Biophys Res Commun 234: 701-706.
T49. Ruppel KM, Spudich JA (1996) Structure-function studies of the myosin motor domain: importance of the 50-kDa cleft. Mol Biol Cell 7: 1123-1136.
T50. Pineda AO, Carrell CJ, Bush LA, Prasad S, Caccia S, et al. (2004) Molecular dissection of Na+ binding to thrombin. J Biol Chem 279: 31842-31853.
T51. Miller SP, Anand GR, Karschnia EJ, Bell GI, LaPorte DC, et al. (1999) Characterization of glucokinase mutations associated with maturity-onset diabetes of the young type 2 (MODY-2): different glucokinase defects lead to a common phenotype. Diabetes 48: 1645-1651.
T52. Guinto ER, Vindigni A, Ayala YM, Dang QD, Di Cera E (1995) Identification of residues linked to the slow-->fast transition of thrombin. Proc Natl Acad Sci U S A 92: 11185-11189.
