# Supplementary material for: Structure-Based Predictive Models for Allosteric Hot Spots
Source: PLoS Comput Biol. 2009 Oct 9;5(10):e1000531. doi: 10.1371/journal.pcbi.1000531 (PMC2748687; doi:10.1371/journal.pcbi.1000531)
Supplement: Table S2 — Independent data set. Given are the protein name, the PDB ID of the inactive state, the PDB ID of the active state, the residue that was mutated, the reference(s) where the effect(s) of the mutation is (are) described, and, in the final column, details of the experiment(s) in which the mutation was characterized. In the final column, first the point mutation(s) is (are) given, and this is followed by a brief synopsis of the experimental results, except for lac repressor where at least 12 amino acid substitutions were made for each residue (The reader may refer to Markiewicz et al. [T44] and Suckow et al. [T45] for details.). Abbreviations used: wt = wild type; coef. = coefficient; repr. = repression; Is = not responsive to inducer (allolactose or isopropyl–D-thiogalactoside); I- = abolished DNA binding or misfolded. (0.09 MB RTF) [file pcbi.1000531.s006.rtf]

Table S2.  Independent data set.  Given are the protein name, the PDB ID of the inactive state, the PDB ID of the active state, the residue that was mutated, the reference(s) where the effect(s) of the mutation is (are) described, and, in the final column, details of the experiment(s) in which the mutation was characterized.  In the final column, first the point mutation(s) is (are) given, and this is followed by a brief synopsis of the experimental results, except for lac repressor where at least 12 amino acid substitutions were made for each residue (The reader may refer to Markiewicz et al. [T44] and Suckow et al. [T45] for details.).   Abbreviations used:  wt=wild type; coef.=coefficient; repr.=repression; Is=not responsive to inducer (allolactose or isopropyl--D-thiogalactoside);        I- = abolished DNA binding or misfolded.
						
HOTSPOTS						
						
Protein system	PDB ID of inactive state	PDB ID of active state	Residue mutated	Reference	Experimental details	
						
glutamate dehydrogense	1nr7	1hwz	ser 448 (bovine:  444)	[T36]; [T37]	S448P; significantly increased IC-50 for GTP	
			phe 440 (bovine: 436)	[T37]	P440L; significantly increased IC-50 for GTP	
			gln 441 (bovine: 437)	[T37]	Q441R; significantly increased IC-50 for GTP	
			ser 445 (bovine: 441)	[T37]; [T38]	S445L; significantly increased IC-50 for GTP	
			gly 446 (bovine: 442)	[T37]; [T38]	G446R and G446D; G446A; both had significantly increased IC-50 for GTP; sig. increased IC-50 for GTP	
			gly 456 (bovine: 452)	[T39]	G456A; significantly reduced Hill coeff. For GTP; significantly increased IC-50 for GTP 	
						
glucokinase	1v4t	1v4s	ile 159	[T40]	I159A; Hill coef. for gluc of 0.96+/-0.04 vs. 1.40+/-0.12 in wt	
			ala 201	[T40]	A201R; Hill coef. for gluc of 0.76+/-0.06 vs. 1.40+/-0.12 in wt	
			val 203	[T40] 	V203E; Hill coef. for gluc of 0.58+/-0.04 vs. 1.40+/-0.12 in wt	
			val 452	[T40] 	V452S ;Hill coef. for gluc of 0.96+/-0.02 vs. 1.40+/-0.12 in wt	
			tyr 214	[T41]	Y214A; Hill coef. for gluc of 1.20 vs. 1.75 in wt 	
			met 210	[T42]; [T43] 	M210T; M210T; Hill coef. for gluc of 1.36+/-0.11 vs. 1.80+/-0.04 in wt; Hill coef. of 1.4 vs. 1.8 in wt	
			val 455	[T42] 	V455M; Hill coef. for gluc of 1.66+/-0.06 vs. 1.80+/-0.04 in wt	
						
lac repressor	1tlf	1efa	lys 84	[T44]; [T45] 	mutations caused Is phenotype and none caused strong I-; mutations caused Is phenotype	
			asp 88	[T44]; [T45] 	mutations caused Is phenotype and none caused strong I-; mutations caused Is phenotype	
			ala 92	[T44]; [T45] 	mutations caused Is phenotype and none caused strong I-; mutations caused Is phenotype	
			val 95	[T44]; [T45] 	mutations caused Is phenotype and none caused strong I-; mutations caused Is phenotype	
			val 96	[T44]; [T45] 	mutations caused Is phenotype and none caused strong I-; mutations caused Is phenotype	
			ser 97	[T44]; [T45] 	mutations caused Is phenotype and none caused strong I-; mutations caused Is phenotype	
						
myosin II	1vom	1fmw	ile 499	[T46] 	I499A; Loss of motility w/ preserved ATPase activity. Uncoupling of converter domain motion from ATPase, remains in post-stroke conformation in presence of excess ATP	
			phe 692	[T46] 	F692A; Loss of motility w/ preserved ATPase activity. Uncoupling of converter domain motion from ATPase, remains in post-stroke conformation in presence of excess ATP	
			phe 487	[T47] 	F487A; reduced coupling between ATP-binding site and actin-binding site, prolonged attachment to actin	
			phe 506	[T47] 	F506A;  reduced coupling between ATP-binding site and actin-binding site, prolonged attachment to actin	
			cys 678	[T48] 	C678S, C678A, C678T, C678G; normal ATPase activity but reduced sliding velocity along actin	
			ser 465	[T49] 	S465V; moved actin filaments at one-tenth speed of wt	
			thr 474	[T49] 	T474P: 5-fold lower actin-activated ATPase than wt; 20-fold slower actin-activated ATPase than wt	
			glu 476	[T49] 	E476Q: 40-fold slower sliding velocity along actin than wt.    E476K:   ATP-dependent binding to actin preserved, but ATPase abolished  	
			asn 464	[T49] 	N464K:  ATP-dependent binding to actin preserved, but ATPase abolished	
						
thrombin	1sgi	1sg8	pro 60c	[T50]	P60cA:  >3-fold change in specificity ratio	
			asp 189	[T50]	D189A:  >3-fold change in specificity ratio	
			ser 214	[T50]	S214A:  >3-fold change in specificity ratio	
			asp 221	[T50]	D221A:  >3-fold change in specificity ratio	
			gly 223	[T50]	G223A:  >3-fold change in specificity ratio	
			val 163	[T50]	V163A:  >3-fold change in specificity ratio	
			thr 172	[T50]	T172A:  >3-fold change in specificity ratio	
			glu 186b	[T50]	E186bA:  >3-fold change in specificity ratio	
			glu 217	[T50]	E217A:  >3-fold change in specificity ratio	


NON-HOTSPOTS
						
Protein system	PDB ID of inactive state	PDB ID of active state	Residue mutated	Reference	Experimental details	
						
glutamate dehydrogenase	1nr7	1hwz	arg 470 (bovine: 466)	[T39] 	R470H; Hill coeff. for GTP and IC-50 for GTP not sig. different from wt.	
			asn 498 (bovine: 494)	[T39] 	N498S; Hill coeff. for GTP and IC-50 for GTP not sig. different from wt.	
						
glucokinase	1v4t	1v4s	tyr 61	[T40] 	Y61S; Hill coef. for gluc of 1.23+/-0.10 vs. 1.40+/-0.12 in wt	
			lys 161 	[T41] 	K161Q; Hill coef. for gluc of 1.72 vs. 1.75 in wt	
			ala 53	[T42]; [T51] 	A53S; A53S; Hill coef. for gluc of 1.80+/-0.03 vs. 1.80+/-0.04 in wt; Hill coef. for gluc of 1.61+/-0.06 vs. 1.57+/-0.05 in wt	
			glu 70	[T42] 	E70K; Hill coef. for gluc of 1.78+/-0.07 vs. 1.80+/-0.04 in wt	
			his 137	[T42] 	H137R; Hill coef. for gluc of 1.80+/-0.03 vs. 1.80+/-0.04 in wt	
			asp 158	[T42] 	D158A; Hill coef. for gluc of 1.70+/-0.03 vs. 1.80+/-0.04 in wt	
			gly 175	[T42] 	G175R; Hill coef. for gluc of 1.74+/-0.01 vs. 1.80+/-0.04 in wt	
			val 182	[T42]; [T43] 	V182M; V182M; Hill coef. for gluc of 1.70+/-0.05 vs. 1.80+/-0.04 in wt; Hill coef. for gluc of 1.7 vs. 1.8 in wt	
			cys 213	[T42]; [T43] 	C213R; C213R; Hill coef. for gluc of 1.76+/-0.04 vs. 1.80+/-0.04 in wt; Hill coef. for gluc of 1.8 vs. 1.8 in wt	
			glu 300	[T42] 	E300K; Hill coef. for gluc of 1.85+/-0.02 vs. 1.80+/-0.04 in wt	
			val 367	[T42]; [T51] 	V367M; Hill coef. for gluc of 1.80+/-0.06 vs. 1.80+/-0.04 in wt; Hill coef. for gluc of 1.55+/-0.03 vs. 1.57+/-0.05 in wt	
						
lac repressor	1tlf 	1efa	gly 103	[T44]; [T45] 	no substitutions caused Is or I- phenotype; tolerant to substitutions/ at least 12 amino acid substitutions tolerated	
			val 104	[T44]; [T45] 	no substitutions caused Is or I- phenotype; tolerant to substitutions/ at least 12 amino acid substitutions tolerated	
			glu 105	[T44]; [T45] 	no substitutions caused Is or I- phenotype; tolerant to substitutions/ at least 12 amino acid substitutions tolerated	
			ala 106	[T44]; [T45] 	no substitutions caused Is or I- phenotype; tolerant to substitutions/ at least 12 amino acid substitutions tolerated	
			lys 108	[T44]; [T45] 	no substitutions caused Is or I- phenotype; tolerant to substitutions/ at least 12 amino acid substitutions tolerated	
			asn 234	[T44]; [T45] 	no substitutions caused Is or I- phenotype; tolerant to substitutions/ at least 12 amino acid substitutions tolerated	
			gly 236	[T44]; [T45] 	no substitutions caused Is or I- phenotype; tolerant to substitutions/ at least 12 amino acid substitutions tolerated	
			ile 237	[T44]; [T45] 	no substitutions caused Is or I- phenotype; tolerant to substitutions/ at least 12 amino acid substitutions tolerated	
			val 238	[T44]; [T45] 	no substitutions caused Is or I- phenotype; tolerant to substitutions/ at least 12 amino acid substitutions tolerated	
			gln 153	[T44]; [T45] 	no substitutions caused Is or I- phenotype; tolerant to substitutions/ at least 12 amino acid substitutions tolerated	
			thr 154	[T44]; [T45] 	no substitutions caused Is or I- phenotype; tolerant to substitutions/ at least 12 amino acid substitutions tolerated	
			pro 155	[T44]; [T45] 	no substitutions caused Is or I- phenotype; tolerant to substitutions/ at least 12 amino acid substitutions tolerated	
			ser 158	[T44]; [T45] 	no substitutions caused Is or I- phenotype; tolerant to substitutions/ at least 12 amino acid substitutions tolerated	
			thr 206	[T44]; [T45] 	no substitutions caused Is or I- phenotype; tolerant to substitutions/ at least 12 amino acid substitutions tolerated	
			arg 207	[T44]; [T45] 	no substitutions caused Is or I- phenotype; tolerant to substitutions/ at least 12 amino acid substitutions tolerated	
			asn 208	[T44]; [T45] 	no substitutions caused Is or I- phenotype; tolerant to substitutions/ at least 12 amino acid substitutions tolerated	
			gln 209	[T44]; [T45] 	no substitutions caused Is or I- phenotype; tolerant to substitutions/ at least 12 amino acid substitutions tolerated	
			gln 211	[T44]; [T45] 	no substitutions caused Is or I- phenotype; tolerant to substitutions/ at least 12 amino acid substitutions tolerated	
			gln 212	[T44]; [T45] 	no substitutions caused Is or I- phenotype; tolerant to substitutions/ at least 12 amino acid substitutions tolerated	
			gln 311	[T44]; [T45] 	no substitutions caused Is or I- phenotype; tolerant to substitutions/ at least 12 amino acid substitutions tolerated	
			ala 312	[T44]; [T45] 	no substitutions caused Is or I- phenotype; tolerant to substitutions/ at least 12 amino acid substitutions tolerated	
			val 313	[T44]; [T45] 	no substitutions caused Is or I- phenotype; tolerant to substitutions/ at least 12 amino acid substitutions tolerated	
			lys 314	[T44]; [T45] 	no substitutions caused Is or I- phenotype; tolerant to substitutions/ at least 12 amino acid substitutions tolerated	
			gly 315	[T44]; [T45] 	no substitutions caused Is or I- phenotype; tolerant to substitutions/ at least 12 amino acid substitutions tolerated	
			asn 316	[T44]; [T45] 	no substitutions caused Is or I- phenotype; tolerant to substitutions/ at least 12 amino acid substitutions tolerated	
			gln 317	[T44]; [T45] 	no substitutions caused Is or I- phenotype; tolerant to substitutions/ at least 12 amino acid substitutions tolerated	
			leu 318	[T44]; [T45] 	no substitutions caused Is or I- phenotype; tolerant to substitutions/ at least 12 amino acid substitutions tolerated	
						
thrombin	1sgi	1sg8	asp 60e	[T50]	D60eA:  >3-fold change in specificity ratio	
			lys 60f	[T50]	K60fA:  >3-fold change in specificity ratio	
			asn 60g	[T50]	N60gA:  >3-fold change in specificity ratio	
			his 71	[T50]	H71A:  >3-fold change in specificity ratio	
			thr 74	[T50]	T74A:  >3-fold change in specificity ratio	
			trp 96	[T50]; [T52]	W96A; W96A: minimal change in transition state coupling free energy;  >3-fold change in specificity ratio	
			arg 97	[T50]	R97A:  >3-fold change in specificity ratio	
			glu 97a	[T50]	E97aA:  >3-fold change in specificity ratio	
			arg 175	[T50]	R175A:  >3-fold change in specificity ratio	
			trp 245	[T50]	W245A:  >3-fold change in specificity ratio	


Supplemental References
T1. Sola M, Lopez-Hernandez E, Cronet P, Lacroix E, Serrano L, et al. (2000) Towards understanding a molecular switch mechanism: Thermodynamic and crystallographic studies of the signal transduction protein CheY. Journal of Molecular Biology 303: 213-225.
T2. Jiang MY, Bourret RB, Simon MI, Volz K (1997) Uncoupled phosphorylation and activation in bacterial chemotaxis - The 2.3 angstrom structure of an aspartate to lysine mutant at position 13 of CheY. Journal of Biological Chemistry 272: 11850-11855.
T3. Zhu XY, Rebello J, Matsumura P, Volz K (1997) Crystal structures of CheY mutants Y106W and T871/Y106W - CheY activation correlates with movement of residue 106. Journal of Biological Chemistry 272: 5000-5006.
T4. Huffman JL, Lu F, Zalkin H, Brennan RG (2002) Role of residue 147 in the gene regulatory function of the Escherichia coli purine repressor. Biochemistry 41: 511-520.
T5. Lu F, Brennan RG, Zalkin H (1998) Escherichia coli purine repressor: Key residues for the allosteric transition between active and inactive conformations and for interdomain signaling. Biochemistry 37: 15680-15690.
T6. Muller G, Hecht B, Helbl V, Hinrichs W, Saenger W, et al. (1995) Characterization of Noninducible Tet Repressor Mutants Suggests Conformational-Changes Necessary for Induction. Nature Structural Biology 2: 693-703.
T7. Kwiatkowski LD, Hui HL, Karasik E, Colby JE, Noble RW (2007) Mutations of the beta N102 residue of HbA not only inhibit the ligand-linked T to R-e state transition, but also profoundly affect the properties of the T state itself. Biochemistry 46: 2037-2049.
T8. Cheng Y, Shen TJ, Simplaceanu V, Ho C (2002) Ligand binding properties and structural studies of recombinant and chemically modified hemoglobins altered at beta 93 cysteine. Biochemistry 41: 11901-11913.
T9. Chang CK, Simplaceanu V, Ho C (2002) Effects of amino acid substitutions at beta 131 on the structure and properties of hemoglobin: Evidence for communication between alpha(1)beta(1)- and alpha(1)beta(2)-subunit interfaces. Biochemistry 41: 5644-5655.
T10. Jeong ST, Ho NT, Hendrich MP, Ho C (1999) Recombinant hemoglobin(alpha 29leucine -> phenylalanine, alpha 96valine -> tryptophan, beta 108asparagine -> lysine) exhibits low oxygen affinity and high cooperativity combined with resistance to autoxidation. Biochemistry 38: 13433-13442.
T11. Kiger L, Klinger AL, Kwiatkowski LD, De Young A, Doyle ML, et al. (1998) Thermodynamic studies on the equilibrium properties of a series of recombinant beta W37 hemoglobin mutants. Biochemistry 37: 4336-4345.
T12. Grant GA, Xu XL, Hu ZQ (2000) Role of an interdomain Gly-Gly sequence at the regulatory-substrate domain interface in the regulation of Escherichia coli. D-3-phosphoglycerate dehydrogenase. Biochemistry 39: 7316-7319.
T13. Grant GA, Hu ZQ, Xu XL (2001) Amino acid residue mutations uncouple cooperative effects in Escherichia coli D-3-phosphoglycerate dehydrogenase. Journal of Biological Chemistry 276: 17844-17850.
T14. Grant GA, Hu ZQ, Xu XL (2001) Specific interactions at the regulatory domain-substrate binding domain interface influence the cooperativity of inhibition and effector binding in Escherichia coli D-3-phosphoglycerate dehydrogenase. Journal of Biological Chemistry 276: 1078-1083.
T15. Auzat I, Lebras G, Garel JR (1995) Hypercooperativity Induced by Interface Mutations in the Phosphofructokinase from Escherichia-Coli. Journal of Molecular Biology 246: 248-253.
T16. Serre MC, Teschner W, Garel JR (1990) Specific Suppression of Heterotropic Interactions in Phosphofructokinase by the Mutation of Leucine-178 into Tryptophan. Journal of Biological Chemistry 265: 12146-12148.
T17. Iancu CV, Mukund S, Fromm HJ, Honzatko RB (2005) R-state AMP complex reveals initial steps of the quaternary transition of fructose-1,6-bisphosphatase. Journal of Biological Chemistry 280: 19737-19745.
T18. Nelson SW, Kurbanov FT, Honzatko RB, Fromm HJ (2001) The N-terminal segment of recombinant porcine fructose-1,6-bisphosphatase participates in the allosteric regulation of catalysis. Journal of Biological Chemistry 276: 6119-6124.
T19. Nelson SW, Choe JY, Honzatko RB, Fromm HJ (2000) Mutations in the hinge of a dynamic loop broadly influence functional properties of fructose-1,6-bisphosphatase. Journal of Biological Chemistry 275: 29986-29992.
T20. Kurbanov FT, Choe JY, Honzatko RB, Fromm HJ (1998) Directed mutations in the poorly defined region of porcine liver fructose 1,6-bispbosphatase significantly affect catalysis and the mechanism of AMP inhibition. Journal of Biological Chemistry 273: 17511-17516.
T21. Shyur LF, Poland BW, Honzatko RB, Fromm HJ (1997) Major changes in the kinetic mechanism of AMP inhibition and AMP cooperativity attend the mutation of Arg(49) in fructose-1,6-bisphosphatase. Journal of Biological Chemistry 272: 26295-26299.
T22. Shyur LF, Aleshin AE, Honzatko RB, Fromm HJ (1996) Biochemical properties of mutant and wild-type fructose-1,6-bisphosphatases are consistent with the coupling of intra- and intersubunit conformational changes in the T- and R-state transition. Journal of Biological Chemistry 271: 33301-33307.
T23. Shyur LF, Aleshin AE, Honzatko RB, Fromm HJ (1996) Site-directed mutagenesis of residues at subunit interfaces of porcine fructose-1,6-bisphosphatase. Journal of Biological Chemistry 271: 3005-3010.
T24. Ladjimi MM, Kantrowitz ER (1988) A Possible Model for the Concerted Allosteric Transition in Escherichia-Coli Aspartate-Transcarbamylase as Deduced from Site-Directed Mutagenesis Studies. Biochemistry 27: 276-283.
T25. Chan RS, Sakash JB, Macol CP, West JA, Tsuruta H, et al. (2002) The role of intersubunit interactions for the stabilization of the T state of Escherichia coli aspartate transcarbamoylase. Journal of Biological Chemistry 277: 49755-49760.
T26. Fetler L, Tauc P, Baker DP, Macol CP, Kantrowitz ER, et al. (2002) Replacement of Asp-162 by Ala prevents the cooperative transition by the substrates while enhancing the effect of the allosteric activator ATP on E-coli aspartate transcarbamoylase. Protein Science 11: 1074-1081.
T27. Buchbinder JL, Guinovart JJ, Fletterick RJ (1995) Mutations in Paired Alpha-Helices at the Subunit Interface of Glycogen-Phosphorylase Alter Homotropic and Heterotropic Cooperativity. Biochemistry 34: 6423-6432.
T28. Kundrot CE, Evans PR (1991) Designing an Allosterically Locked Phosphofructokinase. Biochemistry 30: 1478-1484.
T29. Li R, Zheng Y (1997) Residues of the Rho family GTPases Rho and Cdc42 that specify sensitivity to Dbl-like guanine nucleotide exchange factors. Journal of Biological Chemistry 272: 4671-4679.
T30. Kavanaugh JS, Weydert JA, Rogers PH, Arnone A, Hui HL, et al. (2001) Site-directed mutations of human hemoglobin at residue 35 beta: A residue at the intersection of the alpha 1 beta 1, alpha 1 beta 2, and at alpha 1 alpha 2 interfaces. Protein Science 10: 1847-1855.
T31. Tsai CH, Simplaceanu V, Ho NT, Shen TJ, Wang DJ, et al. (2003) Site mutations disrupt inter-helical H-bonds (alpha 14W-alpha 67T and beta 15W-beta 72S) involved in kinetic steps in the hemoglobin R -> T transition without altering the free energies of oxygenation. Biophysical Chemistry 100: 131-142.
T32. Fang TY, Simplaceanu V, Tsai CH, Ho NT, Ho C (2000) An additional H-bond in the alpha(1)beta(2) interface as the structural basis for the low oxygen affinity and high cooperativity of a novel recombinant hemoglobin (beta L105W). Biochemistry 39: 13708-13718.
T33. Baudin-Creuza V, Vasseur-Godbillon C, Griffon N, Kister J, Kiger L, et al. (1999) Additive effects of beta chain mutations in low oxygen affinity hemoglobin beta F41Y,K66T. Journal of Biological Chemistry 274: 25550-25554.
T34. Tsai CH, Fang TY, Ho NT, Ho C (2000) Novel recombinant hemoglobin, rHb (beta N108Q), with low oxygen affinity, high cooperativity, and stability against autoxidation. Biochemistry 39: 13719-13729.
T35. Biorn AC, Graves DJ (2001) The amino-terminal tail of glycogen phosphorylase is a switch for controlling phosphorylase conformation, activation, and response to ligands. Biochemistry 40: 5181-5189.
T36. Fang J, Hsu BYL, MacMullen CM, Poncz M, Smith TJ, et al. (2002) Expression, purification and characterization of human glutamate dehydrogenase (GDH) allosteric regulatory mutations. Biochemical Journal 363: 81-87.
T37. Stanley CA, Fang J, Kutyna K, Hsu BYL, Ming JE, et al. (2000) Molecular basis and characterization of the hyperinsulinism/hyperammonemia syndrome - Predominance of mutations in exons 11 and 12 of the glutamate dehydrogenase gene. Diabetes 49: 667-673.
T38. De Lonlay P, Benelli C, Fouque F, Ganguly A, Aral B, et al. (2001) Hyperinsulinism and hyperammonemia syndrome: report of twelve unrelated patients. Pediatr Res 50: 353-357.
T39. Zaganas I, Plaitakis A (2002) Single amino acid substitution (G456A) in the vicinity of the GTP binding domain of human housekeeping glutamate dehydrogenase markedly attenuates GTP inhibition and abolishes the cooperative behavior of the enzyme. J Biol Chem 277: 26422-26428.
T40. Zhang J, Li C, Chen K, Zhu W, Shen X, et al. (2006) Conformational transition pathway in the allosteric process of human glucokinase. Proc Natl Acad Sci U S A 103: 13368-13373.
T41. Moukil MA, Veiga-da-Cunha M, Van Schaftingen E (2000) Study of the regulatory properties of glucokinase by site-directed mutagenesis: conversion of glucokinase to an enzyme with high affinity for glucose. Diabetes 49: 195-201.
T42. Davis EA, Cuesta-Munoz A, Raoul M, Buettger C, Sweet I, et al. (1999) Mutants of glucokinase cause hypoglycaemia- and hyperglycaemia syndromes and their analysis illuminates fundamental quantitative concepts of glucose homeostasis. Diabetologia 42: 1175-1186.
T43. Mahalingam B, Cuesta-Munoz A, Davis EA, Matschinsky FM, Harrison RW, et al. (1999) Structural model of human glucokinase in complex with glucose and ATP: implications for the mutants that cause hypo- and hyperglycemia. Diabetes 48: 1698-1705.
T44. Markiewicz P, Kleina LG, Cruz C, Ehret S, Miller JH (1994) Genetic studies of the lac repressor. XIV. Analysis of 4000 altered Escherichia coli lac repressors reveals essential and non-essential residues, as well as "spacers" which do not require a specific sequence. J Mol Biol 240: 421-433.
T45. Suckow J, Markiewicz P, Kleina LG, Miller J, Kisters-Woike B, et al. (1996) Genetic studies of the Lac repressor. XV: 4000 single amino acid substitutions and analysis of the resulting phenotypes on the basis of the protein structure. J Mol Biol 261: 509-523.
T46. Sasaki N, Ohkura R, Sutoh K (2003) Dictyostelium myosin II mutations that uncouple the converter swing and ATP hydrolysis cycle. Biochemistry 42: 90-95.
T47. Tsiavaliaris G, Fujita-Becker S, Batra R, Levitsky DI, Kull FJ, et al. (2002) Mutations in the relay loop region result in dominant-negative inhibition of myosin II function in Dictyostelium. EMBO Rep 3: 1099-1105.
T48. Suzuki Y, Ohkura R, Sugiura S, Yasuda R, Kinoshita K, Jr., et al. (1997) Modulation of actin filament sliding by mutations of the SH2 cysteine in Dictyostelium myosin II. Biochem Biophys Res Commun 234: 701-706.
T49. Ruppel KM, Spudich JA (1996) Structure-function studies of the myosin motor domain: importance of the 50-kDa cleft. Mol Biol Cell 7: 1123-1136.
T50. Pineda AO, Carrell CJ, Bush LA, Prasad S, Caccia S, et al. (2004) Molecular dissection of Na+ binding to thrombin. J Biol Chem 279: 31842-31853.
T51. Miller SP, Anand GR, Karschnia EJ, Bell GI, LaPorte DC, et al. (1999) Characterization of glucokinase mutations associated with maturity-onset diabetes of the young type 2 (MODY-2): different glucokinase defects lead to a common phenotype. Diabetes 48: 1645-1651.
T52. Guinto ER, Vindigni A, Ayala YM, Dang QD, Di Cera E (1995) Identification of residues linked to the slow-->fast transition of thrombin. Proc Natl Acad Sci U S A 92: 11185-11189.
