# Supplementary material for: Structure-Based Predictive Models for Allosteric Hot Spots
Source: PLoS Comput Biol. 2009 Oct 9;5(10):e1000531. doi: 10.1371/journal.pcbi.1000531 (PMC2748687; doi:10.1371/journal.pcbi.1000531)
Supplement: Table S3 — Classification of residues in the independent data set according to the voting scheme of the top 9 highest-precision Hybrid Feature Set models that was used in Structural Analysis of Predicted Hotspots. The numbers in the columns to the right of the true classification are the number of models out of the nine that predicted a hotspot for each residue. *hotspot = residues that perturb allostery for certain mutations, but did not meet our criteria for inclusion as hotspots in the independent data set. NA = residues not included in the independent data set but have structural properties relevant to allostery. (0.16 MB RTF) [file pcbi.1000531.s007.rtf]

Table S3.  Classification of residues in the independent data set according to the voting scheme of the top 9 highest-precision Hybrid Feature Set models that was used in Structural Analysis of Predicted Hotspots.  The numbers in the columns to the right of the true classification are the number of models out of the nine that predicted a hotspot for each residue.   *hotspot = residues that perturb allostery for certain mutations, but did not meet our criteria for inclusion as hotspots in the independent data set.  NA = residues not included in the independent data set but have structural properties relevant to allostery.
Protein system	PDB ID of inactive state	PDB ID of active state	Residue mutated	True class	No. of models predicting hotspot	
					chain A	chain B	chain C	chain D	chain E	chain F	
glutamate 	1nr7	1hwz	ser 444	hotspot	5	5	5	4	5	5	
dehydrogense			phe 436	hotspot	0	0	0	0	0	0	
			gln 437	hotspot	0	0	0	0	0	0	
			ser 441	hotspot	0	2	0	0	0	0	
			gly 442	hotspot	0	3	3	3	1	3	
			gly 452	hotspot	5	5	5	5	5	5	
			arg 466	non-hotspot	0	0	0	0	0	0	
			asn 494	non-hotspot	9	6	7	8	9	7	
											
					chain A 						
glucokinase	1v4t	1v4s	ala 201	hotspot	9						
			val 203	hotspot	9						
			val 452	hotspot	9						
			tyr 214	hotspot	9						
			met 210	hotspot	9						
			val 455	hotspot	8						
			tyr 61	non-hotspot	4						
			ala 53	non-hotspot	3						
			glu 70	non-hotspot	6						
			his 137	non-hotspot	4						
			val 182	non-hotspot	1						
			cys 213	non-hotspot	9						
			glu 300	non-hotspot	9						
			val 367	non-hotspot	9						
			arg 63	NA	8						
			tyr 215	NA	9						
											


					chain A	chain B	chain C 	chain D			
lac repressor	1tlf	1efa	lys 84	hotspot	8	4	9	0			
			asp 88	hotspot	0	0	9	1			
			ala 92	hotspot	0	4	0	5			
			val 95	hotspot	9	9	9	9			
			val 96	hotspot	9	9	9	8			
			ser 97	hotspot	9	9	5	9			
			gly 103	non-hotspot	3	1	4	3			
			val 104	non-hotspot	0	1	1	1			
			glu 105	non-hotspot	3	0	0	0			
			ala 106	non-hotspot	3	1	3	1			
			lys 108	non-hotspot	0	0	1	0			
			asn 234	non-hotspot	0	1	1	0			
			gly 236	non-hotspot	0	0	0	0			
			ile 237	non-hotspot	0	0	0	0			
			val 238	non-hotspot	0	0	0	0			
			gln 153	non-hotspot	0	0	0	0			
			thr 154	non-hotspot	1	4	1	5			
			pro 155	non-hotspot	0	0	0	0			
			ser 158	non-hotspot	6	2	6	2			
			thr 206	non-hotspot	0	0	0	0			
			arg 207	non-hotspot	0	0	0	0			
			asn 208	non-hotspot	0	0	0	1			
			gln 209	non-hotspot	0	0	0	0			
			gln 211	non-hotspot	5	0	0	0			
			gln 212	non-hotspot	0	0	0	0			
			gln 311	non-hotspot	0	0	0	0			
			ala 312	non-hotspot	0	0	2	0			
			val 313	non-hotspot	0	1	0	0			
			lys 314	non-hotspot	0	0	0	0			
			gly 315	non-hotspot	3	0	0	0			
			asn 316	non-hotspot	0	0	2	0			
			gln 317	non-hotspot	3	0	6	1			
			leu 318	non-hotspot	7	2	2	7			
			his 74	*hotspot	6	6	9	9			
			asp 278	*hotspot	9	9	9	9			
											


					chain A						
myosin II	1vom	1fmw	ile 499	hotspot	6						
			phe 692	hotspot	3						
			phe 487	hotspot	3						
			phe 506	hotspot	9						
			cys 678	hotspot	9						
			ser 465	hotspot	9						
			thr 474	hotspot	9						
			glu 476	hotspot	9						
			asn 464	hotspot	9						
			asp 454	NA	9						
			ile 455	NA	9						
			ser 456	NA	9						
			gly 457	NA	9						
			phe 458	NA	9						
			glu 459	NA	9						
											
					chain B						
thrombin	1sgi	1sg8	pro 60C	hotspot	4						
			asp 189	hotspot	7						
			ser 214	hotspot	9						
			asp 221	hotspot	4						
			gly 223	hotspot	0						
			val 163	hotspot	9						
			thr 172	hotspot	6						
			glu 186B	hotspot	0						
			glu 217	hotspot	0						
			asp 60E	non-hotspot	0						
			lys 60F	non-hotspot	1						
			asn 60G	non-hotspot	0						
			his 71	non-hotspot	0						
			thr 74	non-hotspot	0						
			trp 96	non-hotspot	0						
			arg 97	non-hotspot	0						
			glu 97A	non-hotspot	0						
			arg 175	non-hotspot	0						
			trp 245	non-hotspot	0						
			tyr 225	*hotspot	9						
			tyr 184A	*hotspot	6						
