# Supplementary material for: Structure-Based Predictive Models for Allosteric Hot Spots
Source: PLoS Comput Biol. 2009 Oct 9;5(10):e1000531. doi: 10.1371/journal.pcbi.1000531 (PMC2748687; doi:10.1371/journal.pcbi.1000531)
Supplement: Table S4 — Classification of residues whose mutation caused the IS phenotype in at least one residue substitution. The voting scheme of the top 9 highest-precision Hybrid Feature Set models that was used in Structural Analysis of Predicted Hotspots was used for this classification. The numbers in the columns to the right of the residue index are the number of models out of the nine that predicted a hotspot for each residue. (0.07 MB RTF) [file pcbi.1000531.s008.rtf]

Table S4. Classification of residues whose mutation caused the IS phenotype in at least one residue substitution.  The voting scheme of the top 9 highest-precision Hybrid Feature Set models that was used in Structural Analysis of Predicted Hotspots was used for this classification.  The numbers in the columns to the right of the residue index are the number of models out of the nine that predicted a hotspot for each residue.
Residue Index	chain A	chain B	chain C	chain D	
63	9	9	9	6	
64	9	3	0	9	
66	9	9	5	0	
67	9	9	0	9	
68	9	9	2	6	
69	9	9	9	8	
70	9	9	9	9	
71	3	0	5	9	
72	4	0	9	9	
73	4	0	9	9	
74	6	6	9	9	
75	9	5	5	9	
76	9	3	9	9	
77	9	9	9	9	
78	9	9	9	9	
79	9	5	9	0	
80	9	5	9	3	
81	9	9	9	0	
82	9	9	9	3	
83	9	2	9	0	
84	8	4	9	0	
85	7	8	6	2	
87	0	0	3	3	
88	0	0	9	1	
89	2	0	0	0	
92	0	4	0	5	
93	9	9	0	1	
94	9	9	5	9	
95	9	9	9	9	
96	9	9	9	8	
97	9	9	5	9	
98	9	6	9	9	
99	2	9	0	9	
107	0	8	8	6	
110	9	2	9	6	
113	8	0	9	9	
114	9	9	1	7	
119	2	9	6	4	
120	3	4	0	6	
125	9	9	9	9	
127	9	9	6	0	
128	0	6	0	4	
129	0	0	0	0	
136	0	0	0	3	
139	0	0	0	0	
140	0	1	0	2	
141	0	0	0	0	
142	0	0	0	0	
143	0	0	3	0	
148	9	0	5	0	
149	9	0	9	5	
150	5	5	8	5	
151	2	0	9	9	
152	0	0	2	8	
156	0	0	0	0	
159	5	3	7	8	
160	9	2	2	8	
161	9	2	9	9	
163	3	9	9	7	
166	9	0	9	2	
169	0	0	0	0	
170	0	2	1	5	
173	0	0	0	0	
187	5	5	8	9	
188	3	0	3	7	
191	9	4	0	9	
192	4	4	2	8	
193	9	9	9	0	
194	4	2	8	0	
195	2	8	6	0	
196	5	9	3	3	
197	9	9	9	6	
198	0	0	3	0	
200	0	0	0	0	
220	6	9	4	2	
219	5	9	3	9	
222	5	9	5	3	
243	6	6	2	6	
244	9	9	9	8	
245	9	9	9	4	
246	9	8	9	1	
248	9	1	9	0	
249	9	9	9	9	
273	9	6	0	2	
274	9	9	0	5	
275	9	9	8	9	
276	1	9	1	9	
278	9	9	9	9	
279	9	1	6	9	
283	4	0	0	0	
289	4	0	9	9	
290	9	9	9	9	
291	6	1	6	9	
292	9	9	9	2	
293	2	5	3	9	
295	7	3	3	1	
296	9	0	0	5	
297	6	5	9	9	
298	0	0	0	4	
299	5	6	5	0	
300	9	4	4	0	
304	0	0	2	0	
305	0	4	0	6	
319	0	0	9	6	
321	2	8	0	3	
328	4	0	9	0	
329	1	3	1	0	
