# Supplementary material for: Structure-Based Predictive Models for Allosteric Hot Spots
Source: PLoS Comput Biol. 2009 Oct 9;5(10):e1000531. doi: 10.1371/journal.pcbi.1000531 (PMC2748687; doi:10.1371/journal.pcbi.1000531)
Supplement: Table S5 — Average values of features of interest for hotspots and non-hotspots, along with the p-value (unpaired Student's T-test) signifying the statistical significance of the difference in the average value of each feature between hotspots and non-hotspots. Values with a strongly statistically significant difference (p<0.05) between the two classes are indicated by ** and in bold, and those with a moderate statistical significance are indicated by * and in bold italic. For Feature Set 1, dotted lines separate features that are based on dynamic structural features, local contact geometry, network-based features and conservation. (0.02 MB RTF) [file pcbi.1000531.s009.rtf]

Table S5.  Average values of features of interest for hotspots and non-hotspots, along with the p-value (unpaired Student's T-test) signifying the statistical significance of the difference in the average value of each feature between hotspots and non-hotspots.   Values with a strongly statistically significant difference (p<0.05) between the two classes are indicated by ** and in bold, and those with a moderate statistical significance are indicated by * and in bold italic.  For Feature Set 1, dotted lines separate features that are based on dynamic structural features, local contact geometry, network-based features and conservation.
Feature	Average Value for Hotspots	Average Value for Non-hotspots	Statistical Significance of Difference	
def-energ-i	6.11	5.53	p=0.060	
def-energ-a	5.80	5.09	p=0.069	
diff-def-energ	-0.21	-0.57	p=0.23	
msf-i	4.06	5.10	p=0.031**	
msf-a	4.50	5.30	p=0.062	
diff-msf	0.33	0.13	p=0.23	
bfac-i	4.87	5.47	p=0.12	
bfac-a	5.75	5.57	p=0.38	
diff-bfac	0.84	0.071	p=0.056*	
hbond-a	2.00	1.77	p=0.23	
hbond-i	2.18	1.83	p=0.12	
diff-hbond	1.11	0.68	p=0.031**	
at-dens-i	2.17	2.1	p=0.028**	
at-dens-a	2.17	2.11	p=0.054*	
diff-at-dens	0.080	0.079	p=0.48	
node-deg-i	11.59	10.81	p=0.095	
pert-clust-coef-i	-0.078	-0.084	p=0.40	
cons	6.75	6.1	p=0.11	
lse	1.37	1.41	p=0.032**	
Ca-disp	2.36	1.02	p=0.044**	
asa1	20.64	31.64	p=0.019**	
asa2	17.70	35.10	p=0.00039**	
asaavg	19.17	33.37	p=0.0019**	
asasc1	24.07	36.93	p=0.022**	
asasc2	21.07	40.77	p=0.00091**	
asascavg	22.56	38.86	p=0.0031**	
asabb1	10.58	15.93	p=0.11	
asabbavg	9.87	16.73	p=0.039**	
